# The state of population health research performance in the Middle East and North Africa: a meta-research study

**DOI:** 10.1186/s13643-020-01552-x

**Published:** 2021-01-02

**Authors:** Karima Chaabna, Sohaila Cheema, Amit Abraham, Patrick Maisonneuve, Albert B. Lowenfels, Ravinder Mamtani

**Affiliations:** 1 Institute for Population Health, Weill Cornell Medicine—Qatar, Doha, Qatar; 2 Division of Epidemiology and Biostatistics, IEO European Institute of Oncology IRCCS, Milan, Italy; 3 Department of Surgery and Department of Family Medicine, New York Medical College, Valhalla, NY USA

**Keywords:** Research capacity, Middle East and North Africa, Gulf Cooperation Council, Population health, North Africa, Middle East, Systematic review, Bibliometric

## Abstract

**Background:**

Population health (PH) research capacity and performance are essential pillars of evidence-based practice to help address health inequalities. Best evidence is provided by systematic reviews (SRs). None of the published bibliometric analysis specifically assess the production of SRs on PH in the Middle East and North Africa (MENA). The aim of our study is to investigate publication patterns and time trends of SRs reporting PH in the MENA region to evaluate the state of PH research performance in the region.

**Method:**

The study protocol was developed a priori (protocol registration number: CRD42017076736). PubMed was searched. Two independent reviewers screened 5747 identified articles. We investigated author affiliation and collaboration, yearly citations of the SRs and journal information. Joinpoint regression was used to explore these characteristics overtime.

**Results:**

Our meta-research included 387 SRs published between 2008 and 2016 which reported data on PH in 20 MENA countries. Publication of SRs increased over time in journals with impact factor < 4 and in the categories of yearly number of citations < 50 (*p* values ≤ 0.0024). Authors belonging to the region published increasingly (*p* value = 0.0001) over time. Thirty percent of the SRs were from authors solely from the region, while an additional 30% were from the region collaborating with Western country authors. Of these collaborative reviews, 79% were led by authors from the region. However, collaboration in the region (with the exclusion of collaboration with Western country authors) was rare (0.8%). These authors from the region published more in open-access journals while authors from Western countries collaborating or not with authors from the region published more in hybrid or non-open-access journals (*p* value < 0.0001). Collaboration between authors from MENA and Western countries led to published SRs in journals with impact factor ≥ 10. Systematic reviews with global coverage were published more by authors from Western countries, while SRs with country-level coverage were published by authors from the region (*p* value < 0.0001).

**Conclusion:**

The incremental trend of PH SR publications on MENA likely reflects the ongoing improvement in research performance in the region. Authors from the region appear to be taking a lead role in conducting and disseminating MENA PH research. Open-access journals are a major contributor in facilitating MENA research dissemination.

**Systematic review registration:**

PROSPERO registration number CRD42017076736

## Background

Evidence-based practice is not only critical for ensuring the highest quality in patient care but also for policy-making and implementing public health interventions at both local and national levels. Best evidence is generated by well-conducted systematic reviews (SRs), since they critically appraise and synthesize data produced by primary studies addressing a specific research question [1]. The capability of a country to generate detailed knowledge and understanding of its health challenges is essential for developing and implementing preventative strategies to address health problems through evidence-based decision-making [2]. This is typically undertaken through published primary studies and thereafter critically synthesized in SRs. Researchers, policymakers, and clinicians should be informed with the best evidence available pertaining to their country’s population health (PH) in order to identify context-appropriate solutions.

Existing bibliometric analyses evaluate research performance at global and country-levels [3–6]. However, none of them specifically assess the production of SRs on PH in the Middle East and North Africa (MENA). The aim of our study is to investigate publication patterns and time trends of SRs reporting on PH topics in the MENA region to evaluate the state of PH research performance in the region. The objectives of our study are to (1) assess publication patterns and time trends of SRs on PH in MENA, (2) evaluate collaboration in published SRs, (3) identify common topics explored in PH research pertaining to MENA, and (4) explore the role of journals’ access policies in research dissemination from MENA.

## Methods

Our meta-research study is part of the Population Health Publications Assessment Project aiming to assess the methodological quality and the use of gray literature in published SRs on population health in MENA [7–10]. An a priori protocol was registered with the International Prospective Register of Systematic Reviews (PROSPERO registration number CRD42017076736) [11] and published (https://systematicreviewsjournal.biomedcentral.com/articles/10.1186/s13643-018-0751-4) [7]. We primarily conducted a SR of SRs to identify the SRs reporting data on PH in MENA published between January 2008 and December 2016 (2008 was chosen as the point of initiation since this was the publication year of the first version of the Cochrane Handbook for Systematic Reviews of Interventions) [12]. We first published the quality analysis of the literature search conducted in the identified cohort of SRs [8]. Thereafter, bibliometric analysis, as a statistical evaluation of published scientific articles [13], was applied to this cohort of SRs.

### Inclusion and exclusion criteria

We included SRs reporting data on any PH topic within 20 MENA countries, namely Algeria, Bahrain, Djibouti, Egypt, Iraq, Jordan, Kuwait, Lebanon, Libya, Morocco, Oman, Pakistan, Palestine, Qatar, Saudi Arabia, Sudan, Syria, Tunisia, United Arab Emirates, and Yemen. Selection details of the 20 countries have been published [7]. Based on PRISMA-P terminology [14], we defined a SR as a review of primary studies reporting a search strategy for at least one electronic database along with eligibility criteria, which were applied during a multi-stage process of study selection. PH was defined as “the health outcomes of a group of individuals, including the distribution of such outcomes within the group” [15]. Narrative review or systematic analysis synthetizing primary studies which were not identified and selected following a systematic process were excluded.

### Literature search and data management

The literature search was conducted on PubMed [16], the first public digital archives [17] including MEDLINE database, which is also included in Embase [18] and Scopus [19]. We utilized search criteria combining MENA countries, regions and populations’ names and limited to reviews, systematic reviews, and meta-analyses. By searching PubMed, we likely included most of the SRs on PH in MENA published in peer-reviewed journals during 2008–2016. Details of the search strategy have been published [7]. Similar search criteria have been utilized for SRs on PH in MENA and published in peer-reviewed journals [10, 20–23].

A total of 5747 articles were identified. After two independent multi-stage screenings including title/abstract and full-text screening, 387 SRs were included in the analysis (PRISMA flowchart and characteristics of included SRs are in additional files 3 and 4). Data extraction was conducted by two reviewers and cross-checked for accuracy. From each SR the research topic, year of publication, journal’s name, MENA country/countries covered by the SR, and authors’ country of affiliation(s) were extracted.

SR authors were categorized based on the country of their institutional affiliation. Authors were categorized as: belonging to the MENA countries if one of their institutional affiliations was in one of the 20 selected countries; to be from neighboring countries if their institutional affiliations were in a country not included in the 20 selected MENA countries but from other Middle East countries or South Asia or Africa; and from non-MENA and non-neighboring (NMNN) countries if their institutional affiliations were not from the 20 selected MENA countries, other Middle East countries, South Asia, or Africa. Due to the geographic proximity of the neighboring countries, which often share socio-economic and cultural aspects with some of the 20 selected countries, we differentiated those authors affiliated to institutions located in these countries from authors from NMNN countries located, for instance, in Europe, Australia, or the North American continent. The rationale for this is that a few of the neighboring countries such as Iran [24–27], Turkey [27], and Somalia [25, 26] are sometimes included in the definition of the MENA region.

We discussed the health topics addressed by the included SRs according to the disease burden in MENA. Research topics studied by the included SRs were categorized following Global Burden of Disease (GBD) study classification [27]. The three main categories were communicable, maternal, neonatal, and nutritional diseases (GBD code: A); non-communicable diseases (NCDs; GBD code: B); and injuries (GBD code: C). A fourth category “other” was used when the topic did not match any of GBD categories. The GBD disability adjusted life years (DALYs) estimates in the MENA region between 2008 and 2016 for these topics were retrieved from the Institute for Health Metrics and Evaluation website [27].

The journal’s impact factor (JIF) during the year of publication was obtained from the Institute of Scientific Information’s Journal Citation Report (ISI-JCR, ISI http://www.scijournal.org/list-of-impact-factor-journal_I.shtml). Since citations measured by Google Scholar might more appropriately reflect the impact of a publication in the scientific community and in the society (for instance, in dissertations, websites, non-indexed journals, non-English sources, book chapters), the number of citations were collected on Google Scholar [28]. Number of citations per year for each SR was calculated as the total number of citations divided by the number of years since publication. A period of at least 1 year was given between the date of publication of a SR (up to December 31, 2016) and the date of the collection of the number of citations (on January 17, 2018). From the journals’ websites, we collected information on the publisher’s name, open access status, and potential affiliation to a society, association, or university.

### Analysis

Publication pattern and time trends analyses were conducted on the basis of author institutional affiliation as per the following categories: (1) “Inside”—authors affiliated to institutions located in MENA and/or neighboring countries, (2) “Outside”—authors affiliated to institutions located in NMNN countries, and (3) “North-South collaboration”—authors affiliated to institutions located in MENA and/or neighboring countries collaborating with authors from NMNN countries. The time trend for yearly number of publications was assessed using linear regression in unstratified and stratified data. We assessed significance of the association using the *F* test and the goodness-of-fit of the models using adjusted R-squared statistics. We also conducted analyses by yearly number of citations and JIF. Fisher’s exact test was utilized to assess the differences between the number of publications between SRs and journals characteristics. Authors’ affiliation in SRs was used as a proxy for research capacity [4]. Association statistical significance threshold was at 0.01. We used R-3.3.1 software and SPSS V.25 for our analyses.

## Results

### Time trends

A linear statistically significant increase in the annual number of published SRs was identified (from 16 in 2008 to 81 in 2016, 406% increase, Table 1 and Additional file 1). Systematic reviews were increasingly published by (i) “Inside” authors (two to 29, + 1350% increase), (ii) “Outside” authors (eight to 26, + 225% increase), and (iii) by authors from the “North-South collaboration” category (six to 26, + 333% increase). Similarly, a linear statistically significant increase in the annual number of published SRs at country-level coverage was also identified (Adj. *R*^2^ = 0.71, *p* value = 0.0024), from five in 2008 to 18 in 2016 (+ 260% increase).

**Table 1:** Univariate regression analysis of systematic review production time trend by publication characteristics

Publication characteristics	Beta	SE	Adjusted *R*^2^	*p* value*	Time trend
Authors					
North-South collaboration	2.6	0.4	0.83	0.0004	+
Outside MENA	2.1	0.4	0.79	0.0008	+
Inside MENA and/or neighboring countries	3.4	0.5	0.88	0.0001	+
Journal impact factor (JIF)					
0 ≤ JIF < 2	3.3	0.7	0.72	0.0024	+
2 ≤ JIF < 4	3.0	0.6	0.72	0.0021	+
4 ≤ JIF < 6	0.9	0.3	0.44	0.0304	.
JIF ≥ 6	0.8	0.3	0.44	0.0303	.
Yearly number of citations (*n*)					
*n* < 10	5.5	0.7	0.87	0.0015	+
10 ≤ *n* < 50	2.2	0.4	0.78	0.0009	+
50 ≤ *n* < 100	0.3	0.1	0.28	0.0826	.
*n* ≥ 100	0.0	0.1	− 0.1	0.8887	.
Overall^a^	8.1	0.7	0.94	*p* < 0.0001	+

*p value of the *F* test. Association statistical significance threshold was at 0.01. ^a^ The time trend for yearly number of publications was assessed using linear regression in unstratified data. “+”: statistically significant increasing trend. “.”: trend not significant

Between 2008 and 2016, a statistically significant linear increase in the number of published SRs was also demonstrated in the categories 0 ≤ JIF < 2 and 2 ≤ JIF < 4, which represents 90% of the journals tracked by ISI-JCR [29] and for the yearly number of citations *n* < 10 and 10 ≤ *n* < 50. However, in the JIF categories 4 ≤ JIF < 6 and JIF ≥ 6 (i.e*.*, the top 10% and 5% of journals tracked by ISI-JCR, respectively [29]), the increase was not significant, and in the yearly citations categories 50 ≤ *n* < 100 and *n* ≥ 100, only a couple of SRs were published each year. When we excluded studies with global and multi-region coverage, there was also a linear statistically significant increase of published SRs in the JIF < 4 categories (Adj. *R*^2^ = [0.74–0.83], *p* values < 0.0020). Regarding the yearly number of citations, when we excluded SRs with global and multi-region coverage, the increase in the number of published SRs was statistically significant for the category *n* < 10 (from eight in 2008 to 26 in 2016; + 225% increase, Adj. *R*^2^ = 0.87, *p* value = 0.0001) and for the category 10 ≤ *n* < 50 (from one in 2008 to 23 in 2016; + 2200% increase, Adj. *R*^2^ = 0.64, *p* value = 0.0058).

### Yearly number of citations

Nevertheless, we observed a significant increase of yearly number of citations along with the increase of JIF (*p* value < 0.0001, Table 2). In the SRs published in journals with a JIF < 2, 79% (139/176) were cited less than 10 times a year. Conversely, the 11 SRs with over 100 yearly citations were all published in journals with a JIF > 7. Therefore, SRs published in high JIF journals are cited more frequently than SRs published in journals with a low JIF.

**Table 2:** Journal impact factors analysis

		Journal impact factor (JIF)			Total	*p* value*
		JIF < 2	2 ≤ JIF < 4	JIF ≥ 4		
Total	–	176 (100%)	132 (100%)	79 (100%)	387 (100%)	–
Yearly number of citations (*n*)	*n* < 10	139 (79.0%)	68 (51.5%)	14 (17.7%)	221 (57.1%)	< 0.0001
	10 ≤ *n* < 50	37 (21.0%)	55 (41.7%)	47 (59.5%)	139 (35.9%)	
	50 ≤ *n* < 100	0 (0.0%)	9 (6.8%)	7 (8.9%)	16 (4.1%)	
	n ≥ 100	0 (0.0%)	0 (0.0%)	11 (13.9%)	11 (2.8%)	
Journal access	Open	108 (61.4%)	59 (44.7%)	21 (26.6%)	188 (48.6%)	< 0.0001
	Hybrid	59 (33.5%)	71 (53.8%)	45 (57.0%)	175 (45.2%)	
	Non-open	9 (5.1%)	2 (1.5%)	13 (16.5%)	24 (6.2%)	
Publisher	Non-commercial	114 (64.8%)	54 (40.9%)	41 (51.9%)	209 (54.0%)	< 0.0001
	Commercial	62 (35.2%)	78 (59.1%)	38 (48.1%)	178 (46.0%)	
Systematic review geographical coverage	Country-specific	62 (35.2%)	21 (15.9%)	8 (10.1%)	91 (23.5%)	< 0.0001
	Global/multiple regions	31 (17.6%)	42 (31.8%)	44 (55.7%)	117 (30.2%)	
	Middle East and North Africa/ Arab world	35 (19.9%)	31 (23.5%)	7 (8.9%)	73 (18.9%)	
	Middle East/ Asia/ Gulf Cooperation Council	37 (21.0%)	25 (18.9%)	7 (8.9%)	69 (17.8%)	
	North Africa/ East Africa/ Africa	11 (6.3%)	13 (9.8%)	13 (16.5%)	37 (9.6%)	
Systematic review health topic	Alcohol, substance, and nicotine abuses	12 (6.8%)	5 (3.8%)	1 (1.3%)	18 (4.7%)	NS
	Cardiovascular disease	8 (4.5%)	8 (6.1%)	1 (1.3%)	17 (4.4%)	
	Diabetes	6 (3.4%)	12 (9.1%)	3 (3.8%)	21 (5.4%)	
	Other metabolic syndromes	8 (4.5%)	3 (2.3%)	4 (5.1%)	15 (3.9%)	
	Genetics	2 (1.1%)	3 (2.3%)	4 (5.1%)	9 (2.3%)	
	Infectious disease	48 (27.3%)	49 (37.1%)	36 (45.6%)	133 (34.4%)	
	Mental health	18 (10.2%)	8 (6.1%)	2 (2.5%)	28 (7.2%)	
	Nutrition	7 (4.0%)	4 (3.0%)	3 (3.8%)	14 (3.6%)	
	Cancer	19 (10.8%)	5 (3.8%)	4 (5.1%)	28 (7.2%)	
	Other	38 (21.6%)	31 (23.5%)	19 (24.1%)	88 (22.7%)	
	Trauma/Injury/Violence	10 (5.7%)	4 (3.0%)	2 (2.5%)	16 (4.1%)	

*p value of the Fisher’s exact test. Association statistical significance threshold at 0.05 was corrected using Bonferroni method to address multiple testing problem. As we conducted 29 tests, significance threshold was at 0.0017. *NS* not significant

### Authors’ affiliations

**Fig. 1:**
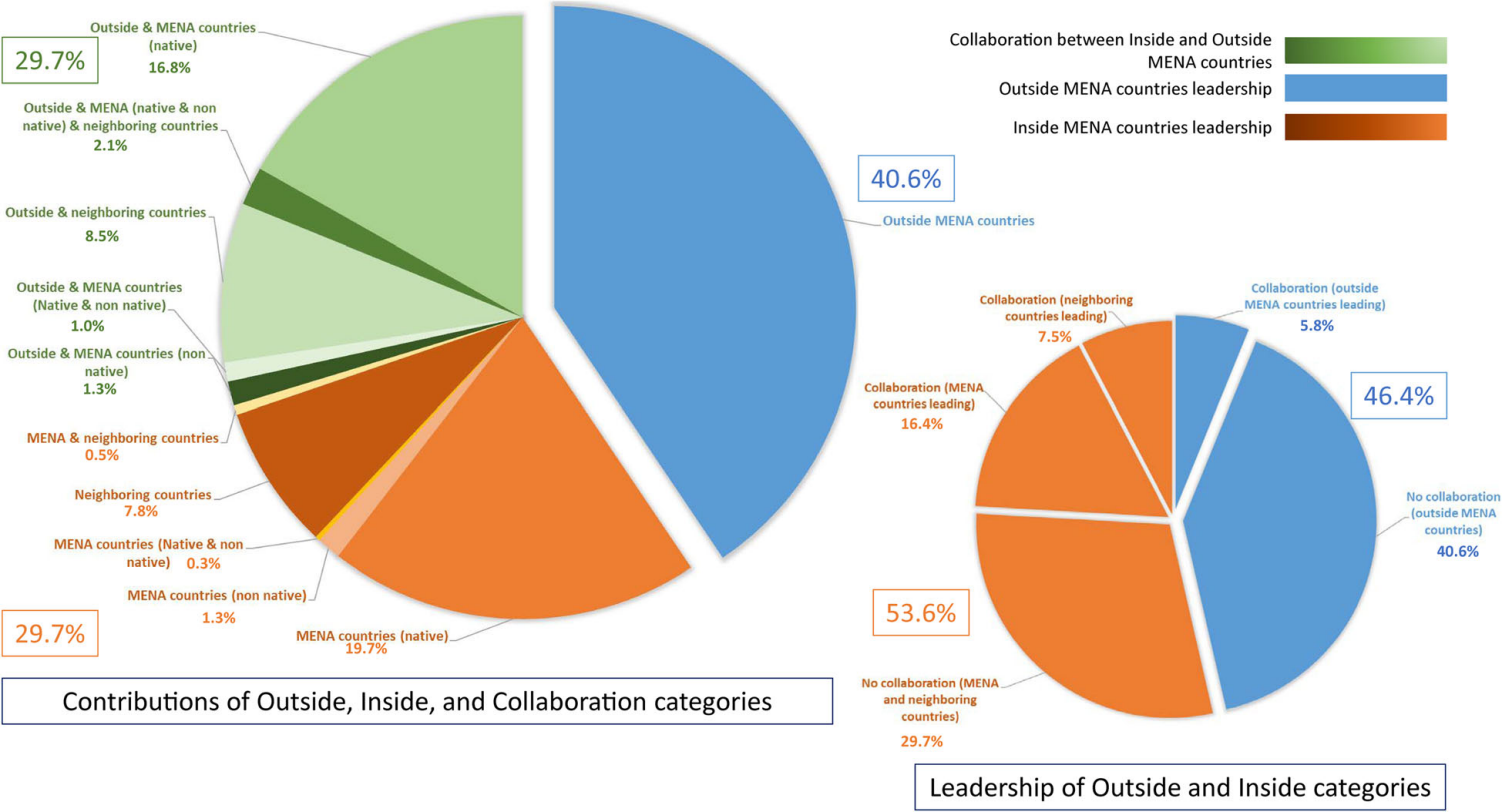
Contribution, collaborations and leadership by author affiliations during 2008–2016. MENA countries (native): authors affiliated to institutions located in MENA countries covered by the systematic review. MENA countries (non-native): authors affiliated to institutions located in MENA countries not covered by the systematic review

Authors from MENA and/or neighboring countries published 30% (“Inside” category: 115/387) of the SRs and collaborated with authors from NMNN countries in an additional 30% (“North-South collaboration” category: 115/387) of the published SRs (Table 3). We identified one SR with authors affiliated solely to institutions located in different MENA countries (Fig. 1). Similarly, South-South collaboration between authors affiliated solely to institutions located in MENA and institutions located in neighboring countries were rare (0.5%; 2/387). Authors from MENA and/or neighboring countries collaborating with authors from NMNN countries were leading the research (first and/or last author position) in 79% of the North-South collaborations (53%; 61/115 SRs from MENA and 26%; 30/115 SRs from neighboring countries). In total, authors from MENA and/or neighboring countries were leading the research in 54% (203/387 SRs) of the published SRs on PH in MENA countries (Fig. 1).

**Table 3:** Analysis of author’s country of affiliation and collaboration productivity

		Authors’ affiliation			Total	*p* value*
		Outside	Collaboration	Inside		
Total	–	157 (100%)	115 (100%)	115 (100%)	387 (100%)	–
Journal impact factor (JIF)	JIF < 2	52 (33.1%)	45 (39.1%)	79 (68.7%)	176 (45.5%)	< 0.0001
	2 ≤ JIF < 4	62 (39.5%)	44 (38.3%)	26 (22.6%)	132 (34.1%)	
	JIF ≥ 4	43 (27.4%)	26 (22.6%)	10 (8.7%)	79 (20.4%)	
Yearly number of citations (*n*)	*n* < 10	64 (40.8%)	74 (64.3%)	83 (72.2%)	221 (57.1%)	< 0.0001
	10 ≤ *n* < 50	72 (45.9%)	35 (30.4%)	32 (27.8%)	139 (35.9%)	
	50 ≤ *n* < 100	12 (7.6%)	4 (3.5%)	0 (0.0%)	16 (4.1%)	
	*n* ≥ 100	9 (5.7%)	2 (1.7%)	0 (0.0%)	11 (2.8%)	
Journal access	Open	59 (37.6%)	55 (47.8%)	74 (64.3%)	188 (48.6%)	< 0.0001
	Hybrid	80 (51.0%)	56 (48.7%)	39 (33.9%)	175 (45.2%)	
	Non-open	18 (11.5%)	4 (3.5%)	2 (1.7%)	24 (6.2%)	
Systematic review geographical coverage	Country-specific	18 (11.5%)	30 (26.1%)	43 (37.4%)	91 (23.5%)	< 0.0001
	Global/multiple regions	85 (54.1%)	19 (16.5%)	13 (11.3%)	117 (30.2%)	
	Middle East and North Africa/ Arab world	21 (13.4%)	23 (20.0%)	29 (25.2%)	73 (18.9%)	
	Middle East/ Asia/ Gulf Cooperation Council	24 (15.3%)	22 (19.1%)	23 (20.0%)	69 (17.8%)	
	North Africa/ East Africa/ Africa	9 (5.7%)	21 (18.3%)	7 (6.1%)	37 (9.6%)	
Systematic review health topic	Alcohol, substance, and nicotine abuses	11 (7.0%)	2 (1.7%)	5 (4.3%)	18 (4.7%)	NS
	Cardiovascular disease	7 (4.5%)	6 (5.2%)	4 (3.5%)	17 (4.4%)	
	Diabetes	7 (4.5%)	8 (7.0%)	6 (5.2%)	21 (5.4%)	
	Other metabolic syndromes	5 (3.2%)	7 (6.1%)	3 (2.6%)	15 (3.9%)	
	Genetics	1 (0.6%)	3 (2.6%)	5 (4.3%)	9 (2.3%)	
	Infectious disease	46 (29.3%)	46 (40.0%)	41 (35.7%)	133 (34.4%)	
	Mental health	8 (5.1%)	12 (10.4%)	8 (7.0%)	28 (7.2%)	
	Nutrition	8 (5.1%)	1 (0.9%)	5 (4.3%)	14 (3.6%)	
	Cancer	12 (7.6%)	4 (3.5%)	12 (10.4%)	28 (7.2%)	
	Other	41 (26.1%)	24 (20.9%)	23 (20.0%)	88 (22.7%)	
	Trauma/Injury/Violence	11 (7.0%)	2 (1.7%)	3 (2.6%)	16 (4.1%)	

*p-value of the Fisher’s exact test. Association statistical significance threshold at 0.05 was corrected using Bonferroni method to address multiple testing problem. As we conducted 29 tests, significance threshold was at 0.0017. NS: not significant

Nevertheless, published SRs reporting data on MENA led by authors from MENA and/or neighboring countries is a recent phenomenon. In 2008–2010, the number of published SRs by “Inside” authors (12%, 7/59) was 2.9 and 4.6 times lower than in the two other categories (“North-South collaboration”: 37%, 22/59 and “Outside”: 51%, 30/59, respectively), while in 2014–2016, “Inside” authors published 34% (70/206) of the SRs, while “North-South collaboration” authors published 32% (66/206) and “Outside” authors 34% (71/206).

### Publisher and journal profiles

The vast majority of the top 10 publishers (out of 62 publishers) with the highest average number of published SRs per journals were non-commercial [30] (90%) and local (70%, data not shown). A wide range of journals (*N* = 243) published SRs reporting data on PH in MENA. Among them, 65 journals (17%, 65/243) published SRs reporting data on PH in MENA exclusively when these SRs had a global and multi-region coverage (85 published SRs out of 387, 22%). Most of the journals (87%, 213/243) published one or two SRs covering at least one of the MENA countries. Eighteen journals (7%, 18/243) published at least four SRs reporting data on PH in MENA (publishing 27% of SRs, 106/387). A large proportion of them were open-access journals (72%, 13/18) and 39% of them were specialized in infectious disease.

### Author affiliation versus journal profile

A significant difference in the type of journals where SRs were published was observed according to author affiliation (Table 3). Most of the SRs by “Inside” authors were published in open-access journals (64%, 74/115), while most of the SRs by “Outside” authors were published in hybrid or non-open-access journals (62%, 98/157). We also observed that journals with an open-access policy have significantly lower JIFs than non-open access journals (Table 2). The number of yearly citations of published SRs varies significantly according to journal access, with lower citation frequencies for open and hybrid-access journals than for non-open access journals (*p* value < 0.0001, Additional file 2).

Among the journals publishing at least four SRs, local journals (28%, 5/18)—which were all open access and with a JIF<2—published the highest number of SRs by “Inside” authors (67%, 22/33 SRs by “Inside” authors), and none published global or multi-regional SRs. Two journals were international with open access, focusing on publishing methodologically sound research regardless of the perceived impact the research may have on their JIF (22%, 23/106 SRs, data not shown). These journals published the second highest number of SRs by “Inside” authors (24%, 8/33 SRs). Additionally, a large proportion of the SRs (61%, 14/23 SRs) published in these journals did not have a global or multi-region coverage.

We also assessed journals with very high impact factors (JIF ≥ 10). Twenty-three SRs (6%, 23/387) were published in journals with a JIF ≥ 10 (data not shown). “Outside” authors published 70% (16/23) of these SRs and the remaining 30% were published by the “North-South collaboration” category. Among the SRs obtaining over 50 citations per year, 78% (21/27 publications) were by “Outside” authors and the remaining 22% were a North-South collaboration. Research collaboration with authors from Western countries appears to be one of the key factors for authors from MENA and/or neighboring countries to be able to publish in high impact factor journals, and thereafter for the articles to be highly cited, likely as a result of being published in such journals.

### Systematic reviews’ geographical coverages

Systematic reviews with country-level or MENA subregion coverage were published significantly more in journals with a JIF < 2, the exception being SRs covering the African areas (Table 2). While SRs with global or multi-regions coverage were published significantly more in journals with a JIF ≥ 4. Consequently, the yearly number of citations was significantly highest for those SRs, while country-level and MENA sub-regional SRs—including those covering the African areas—were predominantly receiving less than 10 citations per year (additional file 2). Systematic reviews with global or multi-region coverage were published significantly more by “Outside” authors (Table 3). While country-level and MENA sub-regional SRs were mainly published by “Inside” authors, the exception being the SRs covering the African area, which were more published by authors in the “North-South collaboration” category.

### Systematic reviews’ health topics

**Fig. 2:**
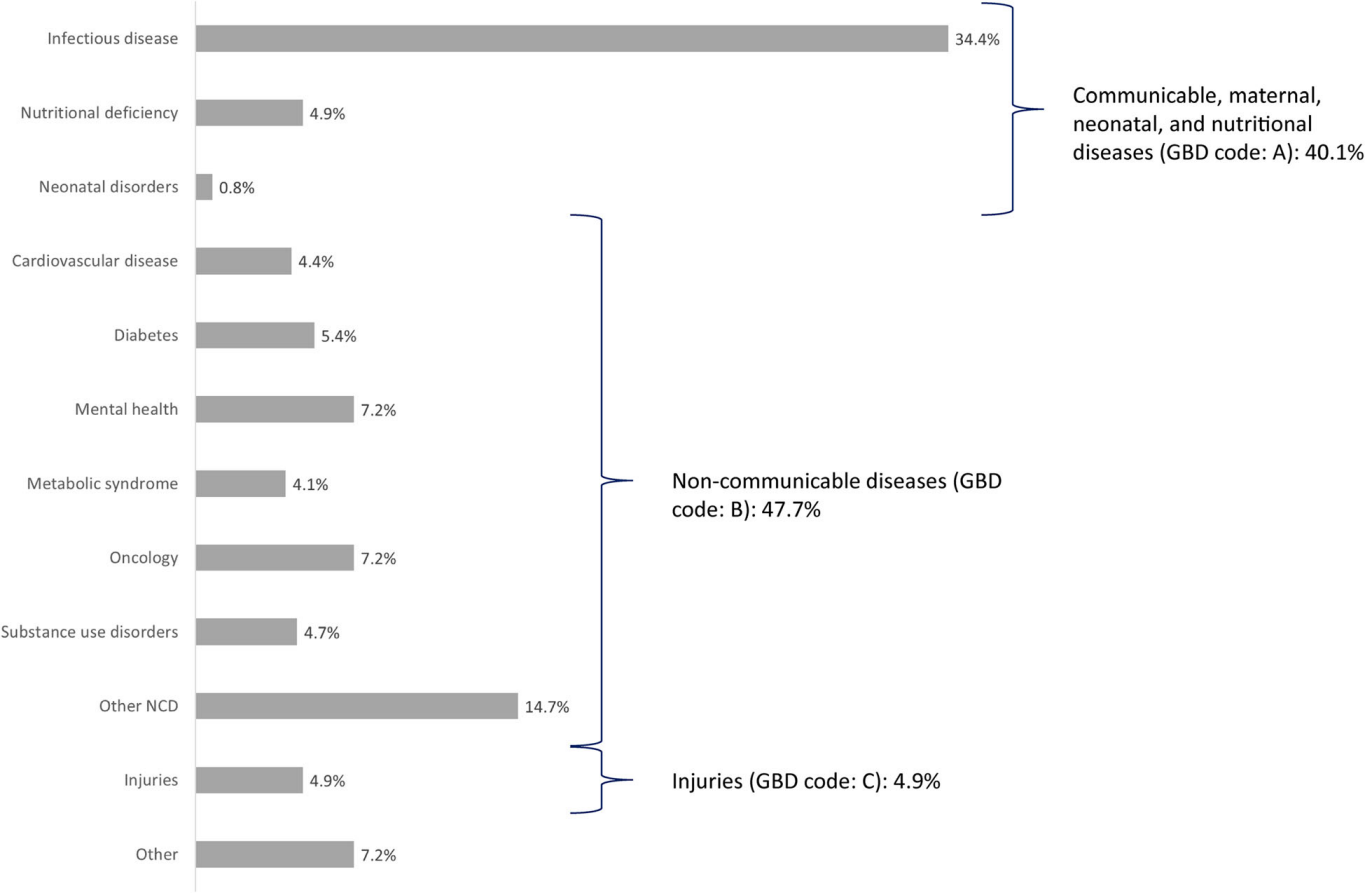
Population health topics covered by the published systematic reviews reporting data on MENA in 2008–2016

**Fig. 3:**
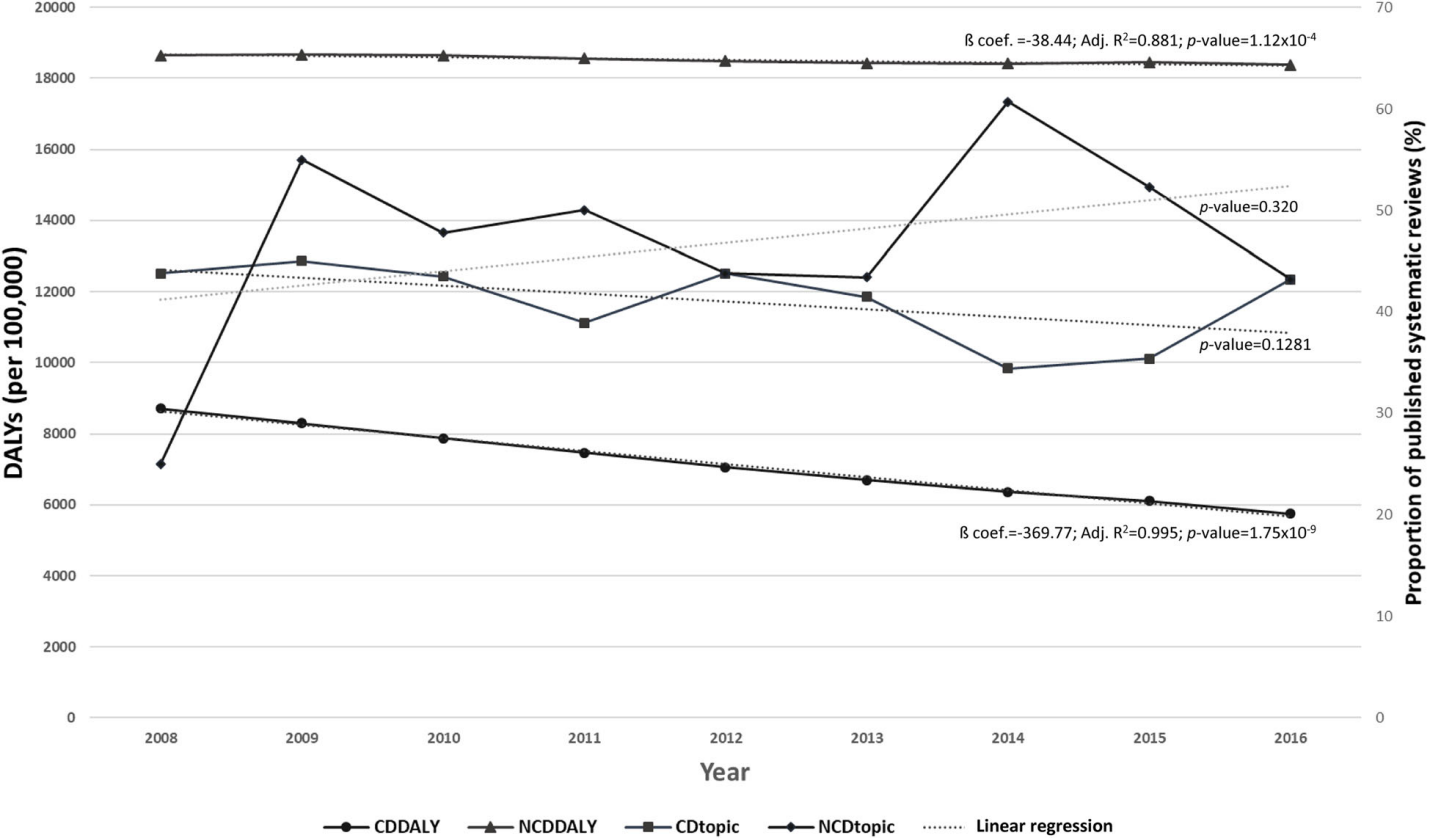
Time trends of DALYs and proportion of published systematic reviews. *CD* communicable, maternal, neonatal, and nutritional diseases (GBD code: A). *NCD* non-communicable diseases (NCD, GBD code: B)

Between 2008 and 2016, the most common topic addressed in the SRs reporting data on the MENA region was NCDs (48%, 185/387, Fig. 2). Among the NCDs, cancer, mental health, metabolic syndrome including diabetes, and cardiovascular disease were commonly researched topics. In 2008, the most common topic addressed in the SRs was infectious disease, including foodborne, blood-borne, and sexually transmitted infections and anti-microbial resistance (44%, 7/16). Between 2008 and 2016, the 18 journals that published at least four SRs reporting data on PH in MENA countries published 41% (54/133) of the SRs on infectious disease. Among these journals, seven specialized in infectious disease topics (39%). Over the years, the proportion of SRs addressing infectious disease topics decreased to 36% (29/81, 2016). Between 2008 and 2016, 86% (133/155) of the SRs addressing the health topic communicable, maternal, neonatal, and nutritional diseases were on infectious disease. No SR was published on maternal disorders. In 2016, three SRs on metabolic syndrome, six on diabetes, three on cardiovascular disease, two on substance use abuses, two on neonatal disorders, and four on nutritional deficiency were published, while in 2008, no SRs on these topics were published. While the ratio between the burden of NCDs and communicable, maternal, neonatal, and nutritional diseases—measured with DALYs—was increasing over time (2.1 in 2008 to 3.2 in 2016), the proportion of published SRs addressing these topics did not change significantly over time and was not proportional to the disease burden (Fig. 3).

## Discussion

The increase in SRs published annually is likely due to an increase in the publication of primary studies conducted in MENA countries. Systematic reviews by authors from the MENA region also increased—both those collaborating with authors from Western countries as well as those not. Our findings suggest ongoing research capacity building and performance improvement in the region. Development of PH research in the region will enhance PH [31] via evidence-based policy-making and interventions [4].

International research collaborations, which have been facilitated by globalization [32], play a major role in the production of population health data and the development of evidence-based solutions pertaining to global health problems [31]. Collaboration between authors from MENA and/or neighboring countries with authors from Western countries has increased for SRs. North-South research collaboration dynamics have been usually reported as being between unequal partners institutions with power remaining with the North [33–36], which raises ethical concerns in the “social world” of global health [37, 38]. Our findings suggest that while published SRs on PH in MENA were previously also dominated by authors affiliated in Western countries, researchers from MENA appear to be taking now the lead by increasingly publishing SRs on topics of importance for the region and collaborating with authors from Western countries. For published SRs, North-South collaboration dynamics appear to be changing in the region with partners from the South identifying their PH research gaps and research capacity needs and addressing them by seeking specific expertise from the North. South-led published SRs are probably not affected by the barriers faced by South-led basic science research, i.e., lack of resources and research skills [39]. Conducting SRs requires minimal equipment as compared to basic science research conducted in wet laboratories, where more sophisticated and expensive equipment maybe required. Additionally, handbooks and guidelines to support researchers in rigorously conducting and reporting SRs are freely available online since the last two decades [12, 40, 41].

While we observed South-South research partnerships, collaboration between authors from MENA and neighboring countries was rare if the collaboration did not include authors from Western countries. Researchers from MENA appear to seek for peers’ expertise mainly from researchers located in Western countries. However, we believe that MENA and neighboring countries experience similar health challenges and could benefit from future cooperation [42]. In the context of globalization, some universities and colleges from North America and Europe established campuses within the region and this has contributed to build in the region research capacity at high levels [43]. Barriers to South-led research previously identified such as donor influence for the choice of research topics and lack of resources and research skills [39] could be addressed by South-South collaborations established with such imported branches of Western institutions, Regional, and country-level collaboration between researchers from MENA and/or neighboring countries can promote PH development by strengthening research capacity and performance via common objectives and shared local expertise.

In addition to making international research collaborations simpler [32], globalization has also facilitated communication of scholarly peer reviewed research by allowing accessibility and usefulness of research through open-access [32]. Authors from the region mainly published SRs that focused on MENA countries or subregions in journals with a JIF < 2 and predominantly in local and international open-access journals. This may be because open-access journals consider methodologically sound research regardless of the perceived impact the research topic may have on their JIF. Journals which are free from JIF dictate play a major role in the global dissemination [44] of the SRs conducted by authors affiliated to institutions located in MENA reporting epidemiological primary studies conducted in MENA. The peer-review process of these journals appear to show less bias against authors affiliated to institutions located in MENA [45–47] and/or non-native English speakers [48]. These journals are therefore contributing to the field of global health by supporting ethic of equity [37, 38] and encouraging research performance improvement and enabling research culture in the region by giving a voice to authors from MENA countries and highlighting health issues pertinent to the region [49]. This enhances PH knowledge pertaining to MENA, which may ultimately lead to better-informed policies contributing to national healthcare policy development and implementation of informed evidence-based healthcare practice [50].

However, while open-access journals enable free reader access to published articles [51], most require a fee for publishing. This may be a barrier for researchers from MENA without appropriate funding and contribute to unbalanced North-South partnership [34]. Notably, some international publishers are addressing this issue by waiving fees for authors who are unable to afford the charge [44]. Publishers located in MENA countries should consider waiving fees for eligible authors to attract additional manuscript submission and to promote wider research dissemination.

Historically, reviews on diseases not prevalent in the “Western world” were unlikely to be of interest to researchers from these countries. This may in turn have resulted in minimal citations of reviews on diseases not prevalent in these countries. Such reviews may then lead to a negative impact on the JIF of a journal if published [52]. However, our review now identifies an increase in published SRs reporting data on MENA PH with yearly number of citations between 10 and 50. This suggests that there were probably both an increase in the interest of journal readers toward PH in MENA as well as in researchers reuse of data on MENA countries reported by these SRs. The progress and increase in global health research projects by researchers from Western countries studying health issues of low- and middle-income countries may have diminished the bias of international journals against the “diseases of poverty,” which are rare or nonexistent in high-income countries [52, 53].

Non-communicable disease is a major PH issue in MENA. The DALYs for NCDs between 2008 and 2016 was at least twice as high as communicable, maternal, neonatal, and nutritional diseases, combined [54]. In 2017, the death rate from non-communicable diseases was five-times higher than death rate from infectious disease [55]. Five of the 10 countries with the highest diabetes prevalence worldwide are in MENA [56]. Additionally, obesity prevalence in MENA countries often exceeds the estimated global average (13%) [57] and in some countries exceeds the prevalence in the USA (e.g., 41% in Qatar in 2012 [58] versus 36% in the USA between 2011 and 2014 [59]). Nevertheless, the proportions of SRs addressing NCDs and communicable, maternal, neonatal, and nutritional diseases topics were similar. This may reflect the development of global health research studying infectious diseases [30] and funding influence on the selection of research topics [39]. A substantial proportion of the SRs appearing in the 18 journals that published at least four SRs reporting data on MENA investigated infectious diseases. The dissemination of this data appears to be facilitated by the interest of journals in specific topics. The topics addressed in the SRs reporting population health in MENA do not appear matching the disease burden in the region. Systematic reviews reporting on NCDs and their risk factors would enable the characterization, synthesis, and further understanding the epidemiology of such diseases in the MENA region, and facilitate evidence-based practice in policy-making and public health interventions.

In 2006, a study identified five factors explaining why research from developing countries was underrepresented in international health literature [53]. Four of these were related to poor research capacity in these countries, while the fifth highlighted the bias of international medical journals. In 2001, under-representation of content on PH in low-income countries in general medical journals with a JIF ≥ 10 was revealed [52]. Eighteen years later, our overview demonstrates that publication bias on SRs from MENA authors continues to exist in high impact factor (JIF > 10), non-open (or hybrid) access journals pertaining to topics reporting on MENA countries. One explanation for the low acceptance rate of SRs from the region may be the influence the research may have on the JIF [60]. Our recommendation to journals that publish manuscripts according to the perceived significance of the research work is to ensure double-blind peer review to avoid any discrimination that could arise because of the names and/or the affiliations of the publication authors [47].

We believe this is the first SR of SRs that investigates research capacity and performance in MENA pertaining to SR publication and explores the role of journals in disseminating SRs on and from MENA. The strength of our report is the large and representative sample of published SRs reporting on all PH topics in MENA. Overall, this study provides objective findings about research performance to researchers, clinicians, and policymakers. Comprehending research performance, its enablers and barriers in MENA may help rationalize staffing decisions and direct appropriate funding from national and international entities [61] which support knowledge acquisition and research capacity building [4]. Our findings will help in decision-making and strategy development regarding the publication of research on PH in MENA, and from MENA researchers. Nevertheless, including articles indexed on PubMed between 2008 and 2016 is technically different from those published between 2008 and 2016 [62]. Therefore, we might have missed some SRs, which were published in 2016 but their indexation was delayed. Additionally, we might have also missed some SRs published in journals not indexed on PubMed. However, the exclusion of those articles would unlikely affect our overall findings and conclusions.

## Conclusion

An increasing number of SRs reporting epidemiological data from MENA countries are being published, reflecting the ongoing research capacity building and performance improvement in the region. Authors affiliated to institutions located in the region appear to be taking the lead in conducting research and disseminating information related to topics of relevance to PH in MENA. Local and international open-access journals, which give due consideration to methodologically sound research regardless of its impact on the journal impact factor are playing an important role in providing a platform to researchers from the region. Future meta-research assessing the productivity of primary studies in MENA countries is required to better understand PH research capacity and performance in the region.

## Supplementary Information

**Additional file 1.** Time trend of annual numbers of systematic reviews on population health in MENA between 2008 and 2016. a: overall trend (unstratified data); b: trends according to author affiliation; c: trends according to journal impact factor (IF); d: trends according to number of yearly citations.

**Additional file 2.** Number of yearly citations analysis.

**Additional file 3.** PRISMA flowchart of searches, screening, and inclusion and exclusion of systematic reviews.

**Additional file 4.** Characteristics of the included systematic reviews on population health in the Middle East and North Africa, 2008-2016.

## Data Availability

All data generated or analyzed during this study are included in this published article and its supplementary information files.

## Abbreviations

AMSTAR: Assessment of Multiple Systematic Reviews
ISI-JCR: Institute of Scientific Information–Journal Citation Report
JIF: Journal impact factor
MENA: Middle East and North Africa region
NCDs: Non-communicable diseases
NMNN: Non-MENA and non-neighboring countries
PH: Population health
PRISMA: Preferred Reporting Items for Systematic Reviews and Meta-Analyses
PRISMA-P: Preferred Reporting Items for Systematic Reviews and Meta-Analysis Protocols
SR: Systematic review
