# Supplementary material for: The state of population health research performance in the Middle East and North Africa: a meta-research study
Source: Syst Rev. 2021 Jan 2;10:1. doi: 10.1186/s13643-020-01552-x (PMC7777412; doi:10.1186/s13643-020-01552-x)
Supplement: Supplementary file 2 — Additional file 2. Number of yearly citations analysis. [file 13643_2020_1552_MOESM2_ESM.docx]

# **The state of population health research performance in the Middle East and North Africa: a systematic review of reviews**

Karima Chaabna^1^*, Sohaila Cheema^1^, Amit Abraham^1^, Patrick Maisonneuve^2^, Albert B Lowenfels^3^, Ravinder Mamtani^1^

1 Institute for Population Health, Weill Cornell Medicine-Qatar, Doha, Qatar

2 Division of Epidemiology and Biostatistics, IEO European Institute of Oncology IRCCS, Milan, Italy

3 Department of Surgery and Department of Family Medicine, New York Medical College, Valhalla, NY, USA

##

## Additional file 2: Number of yearly citations analysis

|  |  | **Number of yearly citations (n)** | | | |  |  |
| --- | --- | --- | --- | --- | --- | --- | --- |
|  |  | **n<10** | **10≤n<50** | **50≤n<100** | **n≥100** | **Total** | ***p*-value*** |
| Total | - | 221 (100%) | 139 (100%) | 16 (100%) | 11 (100%) | 387 (100%) | - |
| Journal access | Open | 109 (49.3%) | 75 (54.0%) | 4 (25.0%) | 0 ( 0.0%) | 188 (48.6%) | <0.0001 |
|  | Hybrid | 103 (46.6%) | 58 (41.7%) | 10 (62.5%) | 4 (36.4%) | 175 (45.2%) |  |
|  | Non-open | 9 ( 4.1%) | 6 ( 4.3%) | 2 (12.5%) | 7 (63.6%) | 24 ( 6.2%) |  |
| Systematic review geographical coverage | Country-specific | 75 (33.9%) | 15 (10.8%) | 1 ( 6.3%) | 0 ( 0.0%) | 91 (23.5%) | <0.0001 |
|  | Global/Multiple regions | 36 (16.3%) | 55 (39.6%) | 15 (93.8%) | 11 (100.0%) | 117 (30.2%) |  |
|  | Middle East and North Africa/ Arab world | 53 (24.0%) | 20 (14.4%) | 0 ( 0.0%) | 0 ( 0.0%) | 73 (18.9%) |  |
|  | Middle East/ Asia/ Gulf Cooperation Council | 37 (16.7%) | 32 (23.0%) | 0 ( 0.0%) | 0 ( 0.0%) | 69 (17.8%) |  |
|  | North Africa/ East Africa/ Africa | 20 ( 9.0%) | 17 (12.2%) | 0 ( 0.0%) | 0 ( 0.0%) | 37 ( 9.6%) |  |
| Systematic review health topic | Alcohol, substance, and nicotine abuses | 13 ( 5.9%) | 5 ( 3.6%) | 0 ( 0.0%) | 0 ( 0.0%) | 18 ( 4.7%) | NS |
|  | Cardiovascular disease | 11 ( 5.0%) | 6 ( 4.3%) | 0 ( 0.0%) | 0 ( 0.0%) | 17 ( 4.4%) |  |
|  | Diabetes | 14 ( 6.3%) | 5 ( 3.6%) | 0 ( 0.0%) | 2 (18.2%) | 21 ( 5.4%) |  |
|  | Other metabolic syndromes | 8 ( 3.6%) | 5 ( 3.6%) | 1 ( 6.3%) | 1 ( 9.1%) | 15 ( 3.9%) |  |
|  | Genetics | 8 ( 3.6%) | 1 ( 0.7%) | 0 ( 0.0%) | 0 ( 0.0%) | 9 ( 2.3%) |  |
|  | Infectious disease | 62 (28.1%) | 56 (40.3%) | 8 (50.0%) | 7 (63.6%) | 133 (34.4%) |  |
|  | Mental health | 22 (10.0%) | 6 ( 4.3%) | 0 ( 0.0%) | 0 ( 0.0%) | 28 ( 7.2%) |  |
|  | Nutrition | 7 ( 3.2%) | 6 ( 4.3%) | 1 ( 6.3%) | 0 ( 0.0%) | 14 ( 3.6%) |  |
|  | Cancer | 17 ( 7.7%) | 11 ( 7.9%) | 0 ( 0.0%) | 0 ( 0.0%) | 28 ( 7.2%) |  |
|  | Other | 50 (22.6%) | 32 (23.0%) | 5 (31.3%) | 1 ( 9.1%) | 88 (22.7%) |  |
|  | Trauma/Injury/Violence | 9 ( 4.1%) | 6 ( 4.3%) | 1 ( 6.3%) | 0 ( 0.0%) | 16 ( 4.1%) |  |

**p*-value of the Fisher’s exact test. Association statistical significance threshold at 0.05 was corrected using Bonferroni method to address multiple testing problem. As we conducted 29 tests, significance threshold was at 0.0017. n: yearly number of citations; NS: not significant
