# Supplementary material for: The state of population health research performance in the Middle East and North Africa: a meta-research study
Source: Syst Rev. 2021 Jan 2;10:1. doi: 10.1186/s13643-020-01552-x (PMC7777412; doi:10.1186/s13643-020-01552-x)

# **The state of population health research performance in the Middle East and North Africa: a systematic review of reviews**

Karima Chaabna^1^*, Sohaila Cheema^1^, Amit Abraham^1^, Patrick Maisonneuve^2^, Albert B Lowenfels^3^, Ravinder Mamtani^1^

1 Institute for Population Health, Weill Cornell Medicine-Qatar, Doha, Qatar

2 Division of Epidemiology and Biostatistics, IEO European Institute of Oncology IRCCS, Milan, Italy

3 Department of Surgery and Department of Family Medicine, New York Medical College, Valhalla, NY, USA

## Additional file 3: PRISMA flowchart of searches, screening, and inclusion and exclusion of systematic reviews


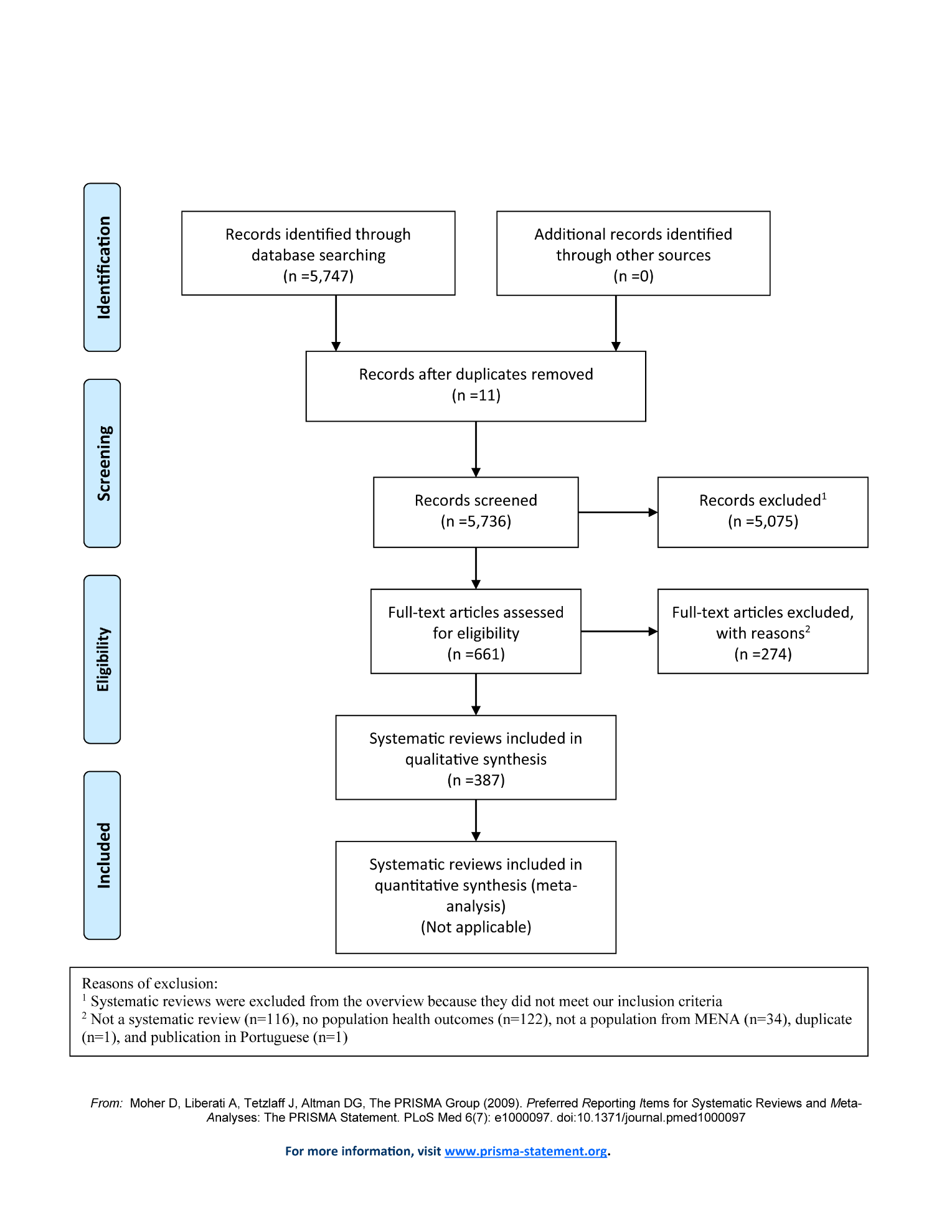

Supplement: Supplementary file 3 — Additional file 3. PRISMA flowchart of searches, screening, and inclusion and exclusion of systematic reviews. [file 13643_2020_1552_MOESM3_ESM.docx]
