# Supplementary material for: The state of population health research performance in the Middle East and North Africa: a meta-research study
Source: Syst Rev. 2021 Jan 2;10:1. doi: 10.1186/s13643-020-01552-x (PMC7777412; doi:10.1186/s13643-020-01552-x)
Supplement: Supplementary file 4 — Additional file 4. Characteristics of the included systematic reviews on population health in the Middle East and North Africa, 2008-2016. [file 13643_2020_1552_MOESM4_ESM.docx]

# **The state of population health research performance in the Middle East and North Africa: a systematic review of reviews**

Karima Chaabna^1^*, Sohaila Cheema^1^, Amit Abraham^1^, Patrick Maisonneuve^2^, Albert B Lowenfels^3^, Ravinder Mamtani^1^

1 Institute for Population Health, Weill Cornell Medicine-Qatar, Doha, Qatar

2 Division of Epidemiology and Biostatistics, IEO European Institute of Oncology IRCCS, Milan, Italy

3 Department of Surgery and Department of Family Medicine, New York Medical College, Valhalla, NY, USA

## Additional file 4: Characteristics of the included systematic reviews on population health in the Middle East and North Africa, 2008-2016

| Reference | Impact Factor^1^ | Google Scholar Citations^2^ | Geographical coverage | Author affiliation | Journal access | Publisher | Population health topic |
| --- | --- | --- | --- | --- | --- | --- | --- |
| NCD Risk Factor Collaboration, 2016 [48] | 47.831 | 504 | Global | Collaboration | Non-Open | Commercial | Oral Medicine |
| Schweizter, 2015 [141] | 44.002 | 546 | Global | Outside | Non-Open | Commercial | Toxicology |
| Danaei, 2011 [259] | 38.278 | 784 | Global | Outside | Non-Open | Commercial | Alcohol/Substance/Nicotine abuse |
| Danaei, 2011 [53] | 38.278 | 2947 | Global | Outside | Non-Open | Commercial | Infection (viral, sexually transmitted) |
| Farzadfar, 2011 [270] | 38.278 | 407 | Global | Outside | Non-Open | Commercial | Mental health |
| Nelson, 2011 [150] | 38.278 | 908 | Global | Outside | Non-Open | Commercial | Infection (viral, sexually transmitted) |
| Mathers, 2010 [197] | 33.633 | 588 | Global | Collaboration | Non-Open | Commercial | Infection (viral, sexually transmitted) |
| Mathers, 2008 [196] | 28.409 | 1194 | Global | Collaboration | Non-Open | Commercial | Neoplasm and genetics |
| Romani, 2015 [123] | 21.372 | 74 | Global | Outside | Non-Open | Commercial | Genetics |
| Tate, 2012 [188] | 19.966 | 862 | Global | Outside | Non-Open | Commercial | Infection (bacterial, sexually transmitted) infection and gynecology |
| Lai, 2016 [192] | 19.864 | 31 | Global | Outside | Non-Open | Commercial | Environment and health status |
| Sperber, 2016 [72] | 16.658 | 43 | Global | Collaboration | Hybrid | Non-commercial | Cardiovascular disease and nutrition |
| Mumtaz, 2010 [199] | 15.617 | 87 | MENA | Collaboration | Open | Commercial | Infection (viral, bloodborne) |
| Steer, 2009 [101] | 15.583 | 328 | Global | Outside | Non-Open | Commercial | Musculoskeletal disorder |
| Eng, 2014 [292] | 14.429 | 35 | Africa | Outside | Open | Commercial | Infection (viral, bloodborne) |
| Mumtaz, 2014 [204] | 14.429 | 59 | MENA | Collaboration | Open | Commercial | Infection (viral, respiratory) |
| Moldecky, 2012 [70] | 12.821 | 2349 | Global | Outside | Hybrid | Non-commercial | Mental health |
| Horton, 2016 [108] | 11.862 | 15 | Global | Collaboration | Open | Commercial | Rheumatology |
| de Martel, 2015 [180] | 11.711 | 118 | Global | Outside | Hybrid | Non-commercial | Genetics |
| Gower, 2014 [169] | 11.336 | 879 | Global | Outside | Hybrid | Non-commercial | Neoplasm and social context |
| Inhorn, 2015 [84] | 11.194 | 137 | Global | Outside | Hybrid | Non-commercial | Mental health |
| Mohd Hanafiah, 2013 [165] | 11.19 | 1930 | Global | Outside | Hybrid | Non-commercial | Neoplasm |
| Stark, 2016 [337] | 10.583 | 45 | Global | Outside | Hybrid | Commercial | Neoplasm |
| Mandil, 2013 [85] | 9.197 | 19 | MENA | Inside | Hybrid | Non-commercial | Alcohol/Substance/Nicotine abuse |
| Gilbert, 2016 [347] | 8.204 | 4 | Multiple regions | Outside | Hybrid | Non-commercial | Neoplasm and social context |
| Marin, 2016 [310] | 7.738 | 23 | Global | Outside | Hybrid | Non-commercial | Neoplasm |
| Alsolamy, 2015 [194] | 7.422 | 23 | Middle East | Inside | Hybrid | Non-commercial | Neoplasm and genetics |
| de Menthon, 2009 [75] | 7.332 | 232 | Global | Outside | Hybrid | Non-commercial | Alcohol/Substance/Nicotine abuse |
| Ng, 2011 [326] | 7.038 | 283 | GCC | Collaboration | Hybrid | Non-commercial | Neoplasm |
| Usher-Smith, 2012 [52] | 6.487 | 99 | Country-level | Outside | Hybrid | Non-commercial | Health status and social context |
| Webb, 2015 [315] | 6.431 | 20 | Global | Outside | Open | Commercial | Alcohol/Substance/Nicotine abuse |
| Nielsen, 2011 [331] | 6.414 | 4 | Country-level | Outside | Hybrid | Non-commercial | Genetics and hemoglobinopathies |
| Abu-Raddad, 2010 [198] | 6.348 | 104 | MENA | Collaboration | Hybrid | Non-commercial | Infection (viral, vector-borne) |
| Wells, 2016 [240] | 6.347 | 9 | Country-level | Collaboration | Hybrid | Non-commercial | Infection (viral, foodborne) |
| Mahdieh, 2016 [81] | 6.342 | 3 | Middle East | Inside | Hybrid | Commercial | Infection (viral, bloodborne) |
| Azevedo, 2016 [140] | 6.296 | 3 | Africa | Collaboration | Open | Non-commercial | Infection (viral, bloodborne) |
| Bruni, 2010 [211] | 6.288 | 786 | Global | Outside | Hybrid | Non-commercial | Trauma/Injury/Violence |
| Stevens, 2013 [343] | 6.17 | 211 | Global | Outside | Hybrid | Non-commercial | Infection (viral, bloodborne) |
| Akhtar, 2016 [333] | 6.077 | 8 | Asia | Inside | Hybrid | Commercial | Mental health |
| Parashar, 2009 [184] | 5.865 | 576 | Global | Outside | Hybrid | Non-commercial | Alcohol/Substance/Nicotine abuse |
| Mabry, 2010 [357] | 5.862 | 87 | GCC | Collaboration | Hybrid | Non-commercial | Diabetes |
| Lip, 2012 [24] | 5.854 | 206 | Global | Outside | Hybrid | Non-commercial | Diabetes |
| Neupane, 2014 [261] | 5.723 | 46 | Asia | Collaboration | Open | Commercial | Physical activity |
| Berrazeg, 2014 [118] | 5.722 | 85 | Global | Collaboration | Open | Commercial | Mental health |
| Hillis, 2016 [387] | 5.705 | 71 | Global | Outside | Hybrid | Non-commercial | Trauma/Injury/Violence |
| Farrokhyar, 2015 [254] | 5.579 | 54 | Global | Outside | Hybrid | Commercial | Infection (bacterial) and antimicrobial resistance |
| Sisson, 2008 [356] | 5.569 | 157 | Global | Outside | Hybrid | Non-commercial | Metabolic syndrome |
| Baandrup, 2014 [298] | 5.417 | 29 | Global | Outside | Hybrid | Non-commercial | Nutrition |
| Corbex, 2014 [278] | 5.417 | 40 | North Africa | Collaboration | Hybrid | Non-commercial | Metabolic disease |
| Tansarli, 2014 [110] | 5.313 | 38 | Africa | Outside | Hybrid | Non-commercial | Ophtalmology |
| Manenzhe, 2015 [99] | 4.919 | 36 | Africa | Inside | Hybrid | Non-commercial | Mental health |
| Bamimore, 2015 [271] | 4.906 | 17 | MENA | Collaboration | Hybrid | Non-commercial | Neoplasm and genetics |
| Katelaris, 2012 [19] | 4.789 | 101 | Multiple regions | Collaboration | Hybrid | Non-commercial | Neoplasm |
| Degenhardt, 2014 [7] | 4.738 | 84 | Global | Outside | Hybrid | Non-commercial | Infection (bacterial) |
| Lehman, 2009 [149] | 4.722 | 147 | Country-level | Outside | Hybrid | Non-commercial | Cardiovascular disease |
| Cheng, 2011 [376] | 4.58 | 122 | Global | Outside | Hybrid | Non-commercial | Mental health |
| van de Meer, 2011 [249] | 4.58 | 123 | Multiple regions | Outside | Hybrid | Non-commercial | Neoplasm |
| Alqahtani, 2015 [136] | 4.575 | 20 | Country-level | Outside | Hybrid | Non-commercial | Anatomy |
| Dean, 2012 [104] | 4.569 | 190 | Global | Outside | Open | Commercial | Allergy |
| Al-Daghri, 2016 [255] | 4.561 | 8 | Country-level | Inside | Hybrid | Commercial | Infection (viral and bacterial, respiratory) and vaccine |
| van de Sande, 2013 [122] | 4.489 | 100 | Global | Outside | Open | Commercial | Metabolic syndrome and mental health |
| El-Ghitany, 2015 [170] | 4.47 | 23 | Global | Inside | Hybrid | Non-commercial | Rheumatology |
| Vanderburg, 2014 [100] | 4.446 | 49 | Africa | Collaboration | Open | Commercial | Cardiovascular disease |
| Ezzikouri, 2013 [144] | 4.412 | 19 | North Africa | Collaboration | Hybrid | Non-commercial | Infection (bacterial) and antimicrobial resistance |
| Chemaitelly, 2015 [171] | 4.411 | 24 | Middle East | Inside | Open | Commercial | Trauma/Injury/Violence |
| Fadlalla, 2015 [172] | 4.411 | 26 | North Africa | Inside | Open | Commercial | Mental health |
| Ogembo, 2015 [213] | 4.411 | 35 | Africa | Outside | Open | Commercial | Infection (viral, respiratory) |
| Olusanya, 2015 [69] | 4.411 | 38 | Multiple regions | Collaboration | Open | Commercial | Nutrition |
| Prasad, 2015 [91] | 4.411 | 26 | Global | Outside | Open | Commercial | Metabolic syndrome |
| Usenbo, 2015 [364] | 4.411 | 27 | Africa | Collaboration | Open | Commercial | Mental health |
| Romdhane, 2012 [76] | 4.315 | 35 | Country-level | Collaboration | Open | Commercial | Metabolic disease |
| Doss, 2016 [77] | 4.259 | 6 | Arab world | Collaboration | Open | Commercial | Metabolic syndrome |
| Hassan, 2016 [238] | 4.246 | 46 | Multiple regions | Outside | Hybrid | Commercial | Diabetes and metabolic syndrome |
| Abdulgader, 2015 [113] | 4.165 | 54 | Africa | Collaboration | Open | Commercial | Diabetes and genetics |
| Riou, 2016 [173] | 4.122 | 25 | Africa | Collaboration | Hybrid | Commercial | Diabetes |
| Dubey, 2016 [283] | 4.111 | 21 | Country-level | Inside | Open | Non-commercial | Diabetes |
| Lihana, 2012 [207] | 4.075 | 114 | Africa | Collaboration | Hybrid | Non-commercial | Neoplasm and genetics |
| Benamer, 2009 [311] | 4.052 | 72 | Arab world | Outside | Hybrid | Non-commercial | Alcohol/Substance/Nicotine abuse |
| Faust, 2009 [308] | 4.016 | 123 | Global | Outside | Non-Open | Non-commercial | Infection (viral, respiratory) |
| Behrouz, 2016 [29] | 3.988 | 11 | Middle East | Outside | Hybrid | Non-commercial | Non-communicable disease and medication adherence |
| Kenyon, 2013 [98] | 3.973 | 136 | Global | Collaboration | Hybrid | Commercial | Infection (viral, bloodborne) |
| Poteat, 2016 [205] | 3.935 | 24 | Global | Outside | Hybrid | Commercial | Trauma/Injury/Violence |
| Bruggmann, 2014 [168] | 3.909 | 185 | Multiple regions | Collaboration | Hybrid | Commercial | Alcohol/Substance/Nicotine abuse |
| Benamer, 2008 [321] | 3.898 | 26 | Arab world | Collaboration | Hybrid | Non-commercial | Metabolic syndrome |
| Humphrey, 2016 [218] | 3.834 | 3 | MENA | Collaboration | Open | Commercial | Mental health |
| Yamamoto, 2014 [31] | 3.829 | 30 | Asia | Collaboration | Hybrid | Non-commercial | Infection (viral, sexually transmitted) |
| Sievert, 2011 [159] | 3.824 | 433 | Multiple regions | Collaboration | Hybrid | Non-commercial | Infection (bacterial) and antimicrobial resistance |
| Pappas, 2009 [128] | 3.819 | 484 | Global | Outside | Hybrid | Commercial | Infection (bacterial, foodborne) |
| Nazeri, 2015 [329] | 3.784 | 10 | Global | Inside | Hybrid | Non-commercial | Mental health |
| Sung, 2014 [288] | 3.774 | 31 | Global | Outside | Hybrid | Commercial | Neurology |
| Stoffaneller, 2015 [339] | 3.759 | 39 | Multiple regions | Outside | Open | Non-commercial | Mental health and neurology |
| Alhyas, 2012 [54] | 3.73 | 60 | GCC | Outside | Open | Commercial | Neoplasm and infection (sexually transmitted) |
| Deckers, 2012 [43] | 3.73 | 177 | Global | Outside | Open | Commercial | Neoplasm |
| Ranasinghe, 2013 [358] | 3.675 | 41 | Asia | Inside | Open | Non-commercial | Diabetes, metabolic syndrome, and Alcohol/Substance/Nicotine abuse |
| Yako, 2016 [58] | 3.639 | 6 | Africa | Collaboration | Hybrid | Non-commercial | Cardiovascular disease |
| Palacios, 2014 [253] | 3.628 | 232 | Global | Outside | Hybrid | Commercial | Metabolic syndrome |
| Itani, 2014 [182] | 3.624 | 4 | MENA | Outside | Hybrid | Commercial | Diabetes and fast |
| Jacobsen, 2010 [181] | 3.572 | 321 | Global | Outside | Hybrid | Commercial | Infection (bacterial) and antimicrobial resistance |
| Adeloye, 2015 [362] | 3.559 | 122 | Global | Outside | Open | Non-commercial | Mental health |
| Azmatullah, 2015 [96] | 3.559 | 18 | Global | Collaboration | Open | Non-commercial | Trauma/Injury/Violence |
| Jiang, 2013 [74] | 3.534 | 87 | Global | Outside | Open | Commercial | Nutrition |
| Kawai, 2012 [187] | 3.492 | 119 | Asia | Outside | Hybrid | Commercial | Cardiovascular disease and nutrition |
| Miles, 2012 [186] | 3.492 | 38 | Country-level | Collaboration | Hybrid | Commercial | Infection (bacterial) and antimicrobial resistance |
| Chanda, 2013 [133] | 3.489 | 9 | Country-level | Collaboration | Open | Commercial | Neoplasm, infection, and smoking |
| D'Acremont, 2010 [125] | 3.489 | 167 | Africa | Outside | Open | Commercial | Gastroenterology |
| Ghandour, 2016 [3] | 3.479 | 7 | Arab world | Inside | Hybrid | Commercial | Population health status |
| Hilger, 2014 [252] | 3.453 | 292 | Global | Outside | Hybrid | Non-commercial | Non-communicable disease |
| Singh, 2016 [67] | 3.452 | 12 | Asia | Collaboration | Hybrid | Non-commercial | Diabetes |
| Midha, 2015 [293] | 3.425 | 89 | Global | Outside | Open | Commercial | Gastroenterology |
| McGrogan, 2011 [307] | 3.396 | 160 | Global | Outside | Hybrid | Non-commercial | Neoplasm |
| Degenhardt, 2011 [6] | 3.383 | 96 | Global | Outside | Hybrid | Non-commercial | Alcohol/Substance/Nicotine abuse |
| Alkhateeb, 2016 [225] | 3.378 | 1 | Arab world | Inside | Hybrid | Non-commercial | Neoplasm and quality of life |
| Stolwijk, 2016 [366] | 3.319 | 33 | Global | Outside | Hybrid | Non-commercial | Infection (viral, bloodborne) |
| Correia Guedes, 2010 [323] | 3.245 | 72 | Global | Outside | Hybrid | Non-commercial | Infection (viral, bloodborne) and neoplasm |
| Benova, 2014 [87] | 3.234 | 14 | Country-level | Outside | Open | Commercial | Infection (viral, bloodborne) |
| Cheng, 2014 [345] | 3.234 | 38 | Asia | Outside | Open | Commercial | Infection (viral, bloodborne) |
| Coleman, 2014 [127] | 3.234 | 18 | Country-level | Collaboration | Open | Commercial | Infection (viral, bloodborne) |
| Jaiswal, 2014 [115] | 3.234 | 18 | Asia | Collaboration | Open | Commercial | Infection (viral, bloodborne) |
| Mathur, 2014 [191] | 3.234 | 16 | Global | Outside | Open | Commercial | Infection (viral, bloodborne) and neoplasm |
| Aljefree, 2015 [38] | 3.226 | 20 | MENA | Outside | Open | Non-commercial | Infection (viral, bloodborne) |
| Singh, 2014 [203] | 3.191 | 10 | Country-level | Collaboration | Hybrid | Commercial | Diabetes and genetics |
| Ali, 2015 [378] | 3.191 | 11 | Country-level | Outside | Non-Open | Commercial | Gynecology |
| Ayer, 2015 [239] | 3.191 | 3 | Country-level | Outside | Non-Open | Commercial | Infection (viral and bacterial, respiratory) and vaccine |
| Al-Khudairy, 2013 [62] | 3.148 | 20 | Middle East | Collaboration | Open | Non-commercial | Neoplasm and infection |
| Khoury, 2011 [185] | 3.118 | 80 | MENA | Outside | Open | Commercial | Infection (viral, bloodborne) |
| Rahimi, 2014 [126] | 3.109 | 51 | Global | Outside | Open | Commercial | Infection (viral, foodborne) |
| Al-Salem, 2016 [130] | 3.08 | 10 | Eastern Africa | Collaboration | Open | Commercial | Infection (viral, respiratory) |
| Mumtaz, 2013 [202] | 3.078 | 22 | Country-level | Collaboration | Hybrid | Non-commercial | Nutrition and social context |
| Gautret, 2016 [195] | 3.055 | 17 | Country-level | Collaboration | Hybrid | Commercial | Oral Medicine |
| Mabry, 2010 [264] | 3.036 | 115 | GCC | Collaboration | Hybrid | Non-commercial | Oral Medicine |
| Shibi, 2009 [105] | 3.032 | 35 | Multiple regions | Collaboration | Hybrid | Non-commercial | Oral Medicine |
| Ott, 2012 [143] | 3.025 | 77 | Global | Outside | Open | Commercial | Infection (bacterial) |
| Khairallah, 2014 [346] | 2.976 | 26 | MENA | Collaboration | Hybrid | Commercial | Physical activity |
| Esan, 2016 [228] | 2.922 | 5 | Africa | Inside | Hybrid | Commercial | Infection (viral, bloodborne) |
| Smith, 2008 [212] | 2.91 | 315 | Global | Outside | Hybrid | Non-commercial | Diabetes |
| Eskandarieh, 2016 [319] | 2.886 | 14 | Asia | Outside | Hybrid | Commercial | Infection (bacterial, sexually transmitted) |
| Mumtaz, 2011 [200] | 2.854 | 33 | MENA | Collaboration | Hybrid | Non-commercial | Alcohol/Substance/Nicotine abuse |
| Popova, 2016 [2] | 2.85 | 2 | Africa | Outside | Hybrid | Non-commercial | Infection (viral, sexually transimitted) |
| Smythe, 2016 [41] | 2.85 | 3 | Multiple regions | Outside | Hybrid | Non-commercial | Alcohol/Substance/Nicotine abuse |
| Bader, 2016 [82] | 2.806 | 2 | MENA | Inside | Open | Commercial | Nutrition |
| Chaabna, 2016 [176] | 2.806 | 11 | Multiple regions | Inside | Open | Commercial | Health Status |
| Gonzalez, 2016 [33] | 2.806 | 1 | Global | Outside | Open | Commercial | Neurology |
| Okpechi, 2016 [306] | 2.806 | 4 | Africa | Collaboration | Open | Commercial | Nutrition |
| Vynnycky, 2016 [42] | 2.806 | 23 | Global | Outside | Open | Commercial | Non-communicable disease and social context |
| Heydarpour, 2015 [318] | 2.784 | 32 | MENA | Inside | Hybrid | Commercial | Neoplasm |
| Furuya-kanamori, 2016 [216] | 2.768 | 50 | Global | Outside | Open | Commercial | Cardiovascular disease and environment |
| Benamer, 2015 [322] | 2.713 | 0 | Arab world | Outside | Hybrid | Commercial | Infection (viral, bloodborne) |
| Agarwal, 2009 [256] | 2.696 | 18 | Global | Inside | Hybrid | Non-commercial | Diabetes and bacterial infection |
| Tanios, 2009 [221] | 2.682 | 38 | Arab world | Collaboration | Hybrid | Commercial | Infection |
| Osio-Salido, 2010 [368] | 2.6 | 77 | Asia | Outside | Hybrid | Non-commercial | Infection (bacterial) |
| Toselli, 2014 [267] | 2.591 | 25 | North Africa | Collaboration | Hybrid | Non-commercial | Infection (viral, bloodborne) |
| Caceres, 2008 [209] | 2.571 | 159 | Global | Outside | Hybrid | Non-commercial | Infection (viral, bloodborne) |
| Mohamoud, 2013 [166] | 2.561 | 269 | Country-level | Collaboration | Open | Commercial | Mental health |
| Huang, 2015 [296] | 2.546 | 12 | Asia | Outside | Open | Non-commercial | Mental health |
| Gasim, 2015 [137] | 2.546 | 7 | Multiple regions | Inside | Open | Commercial | Infection (bacterial) and antimicrobial resistance |
| Alharbi, 2014 [61] | 2.538 | 23 | GCC | Collaboration | Hybrid | Non-commercial | Mental health |
| Zabetian, 2013 [46] | 2.536 | 39 | MENA | Outside | Hybrid | Non-commercial | Alcohol/Substance/Nicotine abuse and neoplasm |
| Gautret, 2016 [90] | 2.532 | 14 | Global | Outside | Open | Non-commercial | Cardiovascular disease and environment |
| Mohamoud, 2016 [177] | 2.532 | 12 | GCC | Inside | Open | Non-commercial | Infection (parasitic, vector-borne) |
| Gu, 2015 [116] | 2.515 | 11 | Multiple regions | Outside | Hybrid | Commercial | Infection (bacterial and viral) and antimicrobial resistance |
| Motlagh, 2009 [64] | 2.511 | 128 | Middle East | Outside | Hybrid | Non-commercial | Infection and vaccine |
| Benamer, 2014 [314] | 2.474 | 9 | MENA | Outside | Hybrid | Non-commercial | Mental health |
| Yazbek, 2016 [183] | 2.468 | 12 | MENA | Inside | Open | Commercial | Alcohol/Substance/Nicotine abuse |
| Chakrabarti, 2011 [121] | 2.457 | 68 | Multiple regions | Inside | Hybrid | Non-commercial | Infection (viral, sexually transmitted) and social context |
| Ameh, 2016 [23] | 2.454 | 1 | Africa | Collaboration | Hybrid | Non-commercial | Infection (viral, sexually transmitted) |
| Bhalla, 2016 [313] | 2.448 | 9 | Arab world | Collaboration | Hybrid | Non-commercial | Neoplasm |
| Gaikwad, 2014 [83] | 2.447 | 26 | Asia | Inside | Hybrid | Non-commercial | Nephrology |
| Ramia, 2012 [161] | 2.44 | 9 | MENA | Inside | Open | Commercial | Infection (bacterial) and antimicrobial resistance |
| Coffin, 2010 [97] | 2.424 | 28 | Global | Outside | Hybrid | Commercial | Infection (viral, sexually transmitted) |
| Devanarayana, 2015 [71] | 2.4 | 10 | Asia | Collaboration | Hybrid | Non-commercial | Nutrition |
| Romdhane, 2011 [78] | 2.391 | 28 | Country-level | Inside | Hybrid | Commercial | Mental health |
| Nansseu, 2016 [263] | 2.369 | 7 | Africa | Collaboration | Open | Commercial | Infection (viral, sexually transmitted) |
| Eshraghian, 2014 [103] | 2.369 | 41 | Middle East | Inside | Open | Commercial | Infection (viral, sexually transmitted) |
| Othman, 2010 [372] | 2.364 | 147 | MENA | Outside | Open | Commercial | Infection (bacterial) |
| Salmon-Rosseau, 2016 [88] | 2.349 | 6 | Country-level | Outside | Hybrid | Non-commercial | Infection (bacterial) and antimicrobial resistance |
| Ali, 2011 [142] | 2.343 | 82 | Country-level | Inside | Open | Commercial | Mental health |
| Attaullah, 2011 [158] | 2.343 | 95 | Country-level | Inside | Open | Commercial | Mental health |
| Riaz, 2011 [153] | 2.343 | 32 | Country-level | Inside | Open | Commercial | Mental health |
| Hung, 2013 [106] | 2.33 | 30 | Asia | Outside | Open | Non-commercial | Nutrition |
| Francis, 2014 [4] | 2.329 | 21 | Eastern Africa | Outside | Hybrid | Non-commercial | Infection (bacterial) and antimicrobial resistance |
| Benamer, 2009 [27] | 2.324 | 78 | Arab world | Outside | Hybrid | Non-commercial | Musculoskeletal disorder |
| Benamer, 2009 [320] | 2.324 | 53 | Arab world | Outside | Hybrid | Non-commercial | Metabolic syndrome and genetics |
| Bos, 2013 [45] | 2.321 | 87 | North Africa | Outside | Open | Commercial | Infection (bacterial, foodborne) |
| Golshan, 2013 [373] | 2.296 | 53 | Asia | Outside | Hybrid | Non-commercial | Infection (viral, bloodborne) |
| Mabry, 2016 [360] | 2.265 | 6 | MENA | Collaboration | Open | Commercial | Mental health |
| Mars, 2014 [244] | 2.264 | 35 | Africa | Outside | Open | Commercial | Alcohol/Substance/Nicotine abuse |
| Al-Khateeb, 2014 [247] | 2.257 | 22 | Arab world | Inside | Hybrid | Commercial | Infection (parasitic) |
| Amara, 2014 [324] | 2.25 | 49 | Multiple regions | Outside | Open | Commercial | Cardiovascular disease |
| Lamri, 2014 [47] | 2.25 | 14 | Country-level | Collaboration | Open | Commercial | Neoplasm and infection |
| Alkabab, 2015 [56] | 2.229 | 8 | Middle East | Collaboration | Open | Non-commercial | Nutrition |
| Ezzikouri, 2013 [164] | 2.217 | 13 | North Africa | Collaboration | Hybrid | Commercial | Gastroenterology |
| Jordans, 2014 [243] | 2.21 | 23 | Asia | Collaboration | Open | Commercial | Respiratory disease |
| Gautret, 2015 [68] | 2.192 | 14 | Country-level | Collaboration | Hybrid | Commercial | Infection (bacterial, foodborne) and antimicrobial resistance |
| Cheng, 2014 [233] | 2.182 | 110 | Global | Outside | Hybrid | Commercial | Infection (bacterial, foodborne) |
| Mehio Sibai, 2010 [39] | 2.173 | 168 | MENA | Collaboration | Hybrid | Commercial | Infection (parasitic) |
| Ali, 2009 [148] | 2.167 | 315 | Country-level | Collaboration | Open | Non-commercial | Infection (bacterial) |
| Tran, 2010 [28] | 2.167 | 78 | MENA | Collaboration | Hybrid | Non-commercial | Neurology |
| DeAntonio, 2016 [135] | 2.157 | 7 | Global | Outside | Hybrid | Commercial | Infection (viral, bloodborne) |
| Haddou Rahou, 2016 [299] | 2.143 | 11 | Arab world | Inside | Open | Commercial | Cardiovascular disease and mental health |
| Zayed, 2016 [51] | 2.14 | 2 | Arab world | Inside | Hybrid | Commercial | Infection (bacterial) and antimicrobial resistance |
| Laraqui, 2015 [290] | 2.137 | 11 | MENA | Collaboration | Open | Commercial | Infection (viral, bloodborne) |
| Kassim, 2015 [13] | 2.134 | 7 | MENA | Outside | Open | Commercial | Infection (viral, sexually transmitted) |
| Tsigga, 2012 [332] | 2.113 | 8 | Country-level | Outside | Open | Non-commercial | Infection (viral, foodborne) |
| Waheed, 2009 [157] | 2.092 | 204 | Country-level | Inside | Open | Commercial | Infection (viral, vector-borne) |
| Jayawardena, 2012 [44] | 2.076 | 150 | Asia | Collaboration | Open | Commercial | Infection (viral, bloodborne and sexually transmitted) |
| Musallam, 2016 [93] | 2.075 | 14 | Middle East | Collaboration | Hybrid | Commercial | Infection (viral, sexually transmitted) |
| Powles, 2013 [340] | 2.063 | 225 | Global | Outside | Open | Commercial | Infection |
| McAlpine, 2016 [382] | 2.048 | 5 | Multiple regions | Outside | Open | Commercial | Infection (parasitic, vector-borne) |
| Hamzeh, 2015 [60] | 2.046 | 9 | Arab world | Inside | Hybrid | Non-commercial | Cardiovascular disease and nutrition |
| Khan, 2013 [30] | 2.039 | 2 | Country-level | Inside | Hybrid | Commercial | Infection (viral, bloodborne) |
| Benamer, 2010 [316] | 2.015 | 30 | Arab world | Collaboration | Open | Non-commercial | Infection (viral, bloodborne) |
| Akl, 2011 [14] | 1.997 | 250 | Global | Collaboration | Open | Commercial | Mental health and Trauma/Injury/Violence |
| Abdullah, 2013 [12] | 1.993 | 41 | Multiple regions | Outside | Open | Non-commercial | Neurology |
| Alkaiyat, 2013 [215] | 1.966 | 13 | MENA | Outside | Hybrid | Non-commercial | Neurology |
| Ghenghesh, 2015 [109] | 1.943 | 0 | Arab world | Inside | Hybrid | Commercial | Neurology |
| Al-Nasser, 2014 [353] | 1.926 | 7 | Arab world | Inside | Hybrid | Non-commercial | Cardiovascular disease |
| Klainin, 2009 [232] | 1.91 | 220 | Asia | Outside | Hybrid | Commercial | Cardiovascular disease |
| Tricco, 2013 [193] | 1.895 | 3 | Multiple regions | Outside | Open | Non-commercial | Metabolic syndrome |
| Akhter, 2011 [363] | 1.885 | 37 | Country-level | Outside | Hybrid | Commercial | Neoplasm |
| Travers, 2013 [230] | 1.87 | 5 | Multiple regions | Outside | Hybrid | Commercial | Oral Medicine |
| Reker, 2014 [167] | 1.859 | 28 | Country-level | Outside | Open | Non-commercial | Ophthalmology |
| Roman, 2013 [379] | 1.842 | 31 | Africa | Inside | Hybrid | Commercial | Infection (viral and bacterial, respiratory) |
| Angalakuditi, 2011 [312] | 1.809 | 16 | Country-level | Outside | Open | Commercial | Infection (viral, bloodborne and foodborne) |
| Cripps, 2011 [386] | 1.805 | 191 | Global | Outside | Hybrid | Non-commercial | Metabolic syndrome and nutrition |
| Abou-Abbass, 2016 [385] | 1.803 | 4 | Country-level | Collaboration | Open | Commercial | Diabetes |
| Creo, 2016 [338] | 1.802 | 4 | Global | Outside | Hybrid | Commercial | Diabetes |
| Amini, 2013 [178] | 1.796 | 25 | MENA | Collaboration | Open | Non-commercial | Infection (parasitic, vector-borne) |
| Mohamed Zaki, 2015 [355] | 1.745 | 2 | Asia | Outside | Non-Open | Non-commercial | Gastroenterology |
| Al-Hashel, 2008 [317] | 1.737 | 27 | MENA | Outside | Hybrid | Commercial | Trauma/Injury/Violence |
| Best, 2010 [336] | 1.727 | 125 | Global | Outside | Hybrid | Non-commercial | Metabolic disease |
| Almutairi, 2014 [10] | 1.716 | 12 | Country-level | Inside | Hybrid | Commercial | Metabolic disease |
| Chippaux, 2008 [371] | 1.707 | 418 | Global | Outside | Hybrid | Commercial | Metabolic disease |
| Raza, 2013 [37] | 1.705 | 13 | Country-level | Outside | Open | Non-commercial | Cardiovascular disease |
| Dimitry, 2012 [235] | 1.7 | 91 | Middle East | Outside | Hybrid | Non-commercial | Diabetes and social context |
| Mustafa, 2014 [367] | 1.696 | 6 | Arab world | Inside | Open | Non-commercial | Metabolic syndrome, nutrition, and physical activity |
| Amawi, 2014 [248] | 1.688 | 12 | Middle East | Collaboration | Hybrid | Commercial | Nephrology |
| Alavian, 2016 [179] | 1.677 | 15 | MENA | Inside | Open | Non-commercial | Infection (viral, bloodborne) |
| Babanejad, 2016 [146] | 1.677 | 9 | MENA | Inside | Open | Non-commercial | Infection (viral, bloodborne) |
| Ghaderi-Zefrehi, 2016 [174] | 1.677 | 11 | Middle East | Inside | Open | Non-commercial | Alcohol/Substance/Nicotine abuse and metabolic syndrome |
| Sadeghi, 2016 [175] | 1.677 | 13 | MENA | Inside | Open | Non-commercial | Metabolic syndrome |
| Marie, 2016 [237] | 1.66 | 8 | Country-level | Collaboration | Open | Commercial | Nephrology |
| Loney, 2013 [361] | 1.646 | 43 | Country-level | Collaboration | Open | Commercial | Toxicology |
| Morovatdar, 2013 [242] | 1.639 | 12 | Middle East | Inside | Hybrid | Non-commercial | Metabolic syndrome |
| Akl, 2013 [15] | 1.594 | 121 | Global | Collaboration | Open | Commercial | Diabetes |
| Boutayeb, 2013 [327] | 1.589 | 51 | Middle East | Inside | Open | Non-commercial | Metabolic syndrome |
| Donnelly, 2015 [35] | 1.579 | 0 | Middle East | Collaboration | Hybrid | Commercial | Infection (viral, sexually transmitted) |
| Mbanya, 2015 [49] | 1.57 | 7 | Africa | Collaboration | Hybrid | Commercial | Infection (viral, sexually transmitted) |
| Mistry, 2015 [268] | 1.566 | 54 | Asia | Collaboration | Hybrid | Non-commercial | Diabetes |
| Ting, 2010 | 1.558 | 47 | Global | Outside | Hybrid | Non-commercial | Infection (viral, bloodborne) |
| Goleg, 2014 [305] | 1.519 | 11 | Country-level | Outside | Hybrid | Commercial | Infection (viral, bloodborne) |
| Donnelly, 2013 [300] | 1.5 | 31 | Arab world | Inside | Open | Non-commercial | Infection (viral, respiratory) |
| John, 2013 [5] | 1.5 | 6 | Middle East | Inside | Open | Non-commercial | Infection (parasitic, skin) |
| Krishna Rao, 2013 [285] | 1.5 | 189 | Asia | Outside | Open | Non-commercial | Infection (bacterial) |
| Boy, 2008 [380] | 1.496 | 105 | MENA | Outside | Non-Open | Commercial | Infection (viral, foodborne) |
| Tosson, 2011 [89] | 1.495 | 16 | Arab world | Collaboration | Hybrid | Non-commercial | Mental health |
| Alhaeli, 2016 [217] | 1.439 | 12 | Country-level | Inside | Open | Non-commercial | Infection/Antimicrobial resistance and social context |
| Sangare, 2015 [112] | 1.422 | 5 | Africa | Collaboration | Hybrid | Non-commercial | Genetics |
| DeNicola, 2015 [66] | 1.417 | 13 | Country-level | Collaboration | Hybrid | Commercial | Infection (viral, bloodborne) |
| Rezaeian, 2010 [241] | 1.383 | 52 | Middle East | Inside | Hybrid | Non-commercial | Infection (viral, bloodborne) |
| Kakde, 2012 [18] | 1.35 | 33 | Asia | Outside | Hybrid | Non-commercial | Infection (viral, bloodborne) |
| AlMarri, 2009 [1] | 1.338 | 59 | GCC | Outside | Hybrid | Non-commercial | Autoimmune disorder |
| Al-Tawfiq, 2015 [120] | 1.333 | 1 | Country-level | Collaboration | Hybrid | Non-commercial | Rheumatology and autoimmune disease |
| Ghenghesh, 2013 [220] | 1.333 | 22 | Country-level | Inside | Open | 2 | Infection (vector-borne) and management |
| Habeb, 2013 [80] | 1.333 | 11 | Country-level | Inside | Open | 2 | Infection (parasitic, vector-borne) |
| Kulczycki, 2011 [377] | 1.328 | 83 | MENA | Outside | Non-Open | Commercial | Infection (parasitic, vector-borne) |
| Shaik, 2015 [295] | 1.312 | 3 | GCC | Inside | Open | Non-commercial | Infection |
| Baddoura, 2011 [274] | 1.295 | 12 | Middle East | Inside | Hybrid | Non-commercial | Infection (bacterial) and antimicrobial resistance |
| Bourne, 2013 [344] | 1.271 | 27 | Global | Collaboration | Hybrid | Non-commercial | Infection (fungal, respiratory) |
| Gasim, 2013 [151] | 1.267 | 36 | Multiple regions | Inside | Open | Commercial | Nephrology |
| Hammad, 2016 [258] | 1.262 | 2 | Country-level | Outside | Non-Open | Non-commercial | Trauma/Injury/Violence |
| Al-Bluwi, 2014 [349] | 1.259 | 9 | Country-level | Inside | Hybrid | Non-commercial | Metabolic syndrome |
| Khan, 2014 [350] | 1.259 | 8 | Arab world | Inside | Hybrid | Non-commercial | Neurology |
| Alavian, 2012 [160] | 1.245 | 13 | MENA | Inside | Open | Non-commercial | Neurology and autoimmune disease |
| Dubey, 2015 [279] | 1.24 | 40 | Country-level | Inside | Open | Non-commercial | Nephrology |
| Irshad, 2015 [294] | 1.24 | 0 | Country-level | Inside | Open | Non-commercial | Neurology |
| Van-Lume, 2013[138] | 1.219 | 18 | Country-level | Outside | Open | Non-commercial | Neurology |
| Seoud, 2012 [214] | 1.207 | 27 | MENA | Inside | Hybrid | Non-commercial | Neurology |
| Kadir, 2008 [370] | 1.204 | 41 | Country-level | Collaboration | Hybrid | Non-commercial | Neurology |
| Al-Harthi, 2013 [354] | 1.195 | 3 | Arab world | Outside | Hybrid | Non-commercial | Mental health |
| Yammine, 2013 [22] | 1.159 | 16 | Global | Inside | Hybrid | Non-commercial | Mental health |
| DeAntonio, 2016 [92] | 1.159 | 6 | Multiple regions | Outside | Hybrid | Non-commercial | Metabolic syndrome |
| Deek, 2015 [26] | 1.122 | 5 | Country-level | Collaboration | Hybrid | Non-commercial | Diabetes and nutrition |
| Tajvar, 2013 [86] | 1.119 | 16 | Middle East | Outside | Hybrid | Non-commercial | Nutrition |
| Sadat-Ali, 2012 [273] | 1.103 | 31 | Country-level | Inside | Hybrid | Commercial | Cardiovascular disease and metabolic syndrome |
| Bakare, 2011 [226] | 1.068 | 71 | Africa | Collaboration | Open | Commercial | Physical activity |
| Tailakh, 2014 [262] | 1.042 | 25 | Arab world | Outside | Hybrid | Commercial | Non-communicable disease and nutrition |
| Puvanachandra, 2012 [384] | 1.042 | 25 | Country-level | Collaboration | Hybrid | Commercial | Physical activity |
| Kouyoumjian, 2013 [201] | 1.037 | 17 | Country-level | Collaboration | Hybrid | Non-commercial | Nutrition |
| Barzangi, 2014 [352] | 1.03 | 4 | Eastern Africa | Outside | Hybrid | Non-commercial | Ophthalmology |
| Fleming, 2009 [63] | 0.953 | 20 | Asia | Outside | Non-Open | Non-commercial | Ophthalmology |
| Almaguer, 2014 [303] | 0.927 | 64 | Global | Outside | Open | Non-commercial | Ophthalmology |
| Yammine, 2016 [359] | 0.919 | 2 | Country-level | Inside | Hybrid | Non-commercial | Genetics |
| Gwer, 2013 [309] | 0.871 | 10 | Multiple regions | Collaboration | Hybrid | Commercial | Trauma/Injury/Violence |
| Dardas, 2016 [231] | 0.867 | 8 | Arab world | Outside | Hybrid | Commercial | Metabolic disease |
| Serbessa, 2016 [210] | 0.861 | 0 | Eastern Africa | Collaboration | Hybrid | Commercial | Nutrition |
| Nasser, 2015 [32] | 0.78 | 12 | Middle East | Inside | Open | Non-commercial | Neurology |
| Al-Jaber, 2016 [287] | 0.709 | 12 | Arab world | Inside | Open | Commercial | Pain management |
| Abozaid, 2013 [163] | 0.705 | 8 | Country-level | Collaboration | Open | Non-commercial | Alcohol/Substance/Nicotine abuse |
| Alhurishi, 2011 [277] | 0.659 | 22 | Middle East | Outside | Open | Non-commercial | Infection (viral, bloodborne) |
| Abubakar, 2016 [190] | 0.628 | 3 | MENA | Collaboration | Open | Non-commercial | Genetics |
| Bashour, 2016 [152] | 0.628 | 0 | Country-level | Inside | Open | Non-commercial | Infection (parasitic, vector-borne) |
| Fehling, 2016 [375] | 0.628 | 4 | MENA | Outside | Open | Non-commercial | Neurology and genetics |
| Ahmad, 2016 [342] | 0.616 | 0 | Country-level | Collaboration | Open | Non-commercial | Trauma/Injury/Violence |
| Mehboob, 2016 [257] | 0.616 | 0 | Country-level | Inside | Open | Non-commercial | Physical activity |
| Meo, 2016 [50] | 0.616 | 5 | Country-level | Inside | Open | Non-commercial | Neoplasm and genetics |
| Meo, 2016 [55] | 0.616 | 0 | Country-level | Inside | Open | Non-commercial | Infection (bacterial) |
| Almoallim, 2014 [365] | 0.588 | 7 | Country-level | Inside | Open | Commercial | Infection (viral, sexually transmitted) |
| Mansuri, 2015 [383] | 0.562 | 19 | Country-level | Inside | Open | Commercial | Infection (viral, sexually transmitted) |
| Umar, 2009 [156] | 0.554 | 12 | Country-level | Inside | Open | Non-commercial | Infection (bacterial) |
| Khan, 2013 [348] | 0.554 | 26 | Country-level | Inside | Open | Commercial | Infection (viral, vector-borne) |
| Alhraiwil, 2015 [224] | 0.541 | 13 | Arab world | Collaboration | Open | Non-commercial | Infection (fungal/bacterial) and dermatology |
| Nasser, 2011 [234] | 0.427 | 5 | Arab world | Inside | Hybrid | Commercial | Infection (bacterial) |
| Ansarie, 2014 [134] | 0.414 | 3 | Country-level | Inside | Open | Non-commercial | Diabetes |
| Mirmiran, 2012 [330] | 0.405 | 17 | Middle East | Collaboration | Open | Non-commercial | Genetics and treatment |
| Bajubair, 2008 [147] | 0.396 | 26 | Country-level | Inside | Open | Commercial | Health status and social context |
| Naseer, 2014 [94] | 0.353 | 4 | Country-level | Inside | Open | Non-commercial | Infection (viral, bloodborne) |
| Zahidie, 2013 [229] | 0.318 | 18 | Country-level | Inside | Open | Non-commercial | Infection (viral, bloodborne) |
| Shah, 2015 [11] | 0.166 | 3 | Country-level | Inside | Open | Commercial | Ophthalmology |
| Gasim, 2013 [145] | 0 | 18 | Arab world | Inside | Open | Commercial | Infection (parasitic, vector-borne) |
| Jroundi, 2015 [189] | 0 | 6 | Country-level | Collaboration | Open | Non-commercial | Dermatology |
| Alananzeh, 2016 [301] | 0 | 0 | Arab world | Outside | Open | Non-commercial | Infection (viral, bloodborne) |
| Kronfol, 2014 [236] | 0 | 7 | GCC | Inside | Hybrid | Commercial | Cardiovascular disease and genetics |
| Alhazzazi, 2016 [281] | 0 | 1 | Country-level | Inside | Open | Non-commercial | Infection (bacterial) and antimicrobial resistance |
| Alsanosy, 2014 [8] | 0 | 22 | Country-level | Inside | Open | Non-commercial | Gastroenterology and vaccine |
| Stern, 2008 [155] | 0 | 28 | Global | Outside | Open | Commercial | Infection (viral, respiratory) |
| Kahsay, 2016 [117] | 0 | 7 | Multiple regions | Inside | Open | Commercial | Infection (viral, sexually transmitted) |
| Cherbal, 2012 [289] | 0 | 3 | North Africa | Inside | Hybrid | Commercial | Nephrology |
| Bhikoo, 2011 [276] | 0 | 30 | Multiple regions | Outside | Open | Commercial | Gastroenterology |
| Khedmat, 2013 [102] | 0 | 27 | Middle East | Inside | Open | Non-commercial | Infection |
| Abou Abbas, 2015 [272] | 0 | 12 | Middle East | Collaboration | Hybrid | Non-commercial | Rheumatology |
| Tapp, 2008 [374] | 0 | 49 | Country-level | Outside | Open | Commercial | Congenital anomaly |
| Hasan, 2014 [260] | 0 | 1 | Country-level | Inside | Hybrid | Commercial | Diabetes |
| Bassil, 2013 [251] | 0 | 73 | MENA | Inside | Open | Commercial | Nutrition |
| Al-Qasem, 2011 [325] | 0 | 23 | Middle East | Outside | Open | Non-commercial | Toxicology |
| Harbour, 2011 [9] | 0 | 5 | Country-level | Outside | Open | Non-commercial | Alcohol/Substance/Nicotine abuse and social context |
| Mirmiran, 2010 [265] | 0 | 70 | MENA | Inside | Open | Non-commercial | Metabolic syndrome |
| Elzubeir, 2010 [222] | 0 | 60 | MENA | Collaboration | Open | Non-commercial | Environment and health status |
| Abu-Raddad, 2010 [208] | 0 | 32 | MENA | Collaboration | Open | Commercial | Infection (viral and parasitic, bloodborne and vector-borne) |
| Hassan, 2014 [57] | 0 | 10 | Multiple regions | Inside | Open | Commercial | Rheumatology |
| Peleteiro, 2008 [302] | 0 | 27 | Global | Outside | Open | Non-commercial | Oral Medicine |
| Hamzeh, 2016 [59] | 0 | 5 | Arab world | Inside | Hybrid | Non-commercial | Anatomy |
| Hussein, 2016 [297] | 0 | 6 | North Africa | Collaboration | Open | Non-commercial | Neoplasm |
| Sharma, 2016 [341] | 0 | 13 | Asia | Outside | Open | Commercial | Anatomy |
| Musaiger, 2011 [334] | 0 | 73 | Arab world | Inside | Open | Non-commercial | Neoplasm and genetics |
| Shahid, 2008 [246] | 0 | 31 | Asia | Collaboration | Hybrid | Non-commercial | Neoplasm |
| Velayati, 2015 [119] | 0 | 11 | Middle East | Collaboration | Hybrid | Non-commercial | Rheumatology |
| El-Zaemey, 2015 [17] | 0 | 9 | Multiple regions | Outside | Open | Non-commercial | Infection (viral, bloodborne) |
| Razavi, 2016 [219] | 0 | 0 | Country-level | Inside | Open | Non-commercial | Oral Medicine |
| Najjar, 2010 [275] | 0 | 96 | Arab world | Outside | Hybrid | Commercial | Trauma/Injury/Violence |
| Mamishi, 2014 [114] | 0 | 11 | Asia | Inside | Open | Non-commercial | Genetics |
| Moradi, 2016 [206] | 0 | 0 | Multiple regions | Collaboration | Open | Non-commercial | Neurology |
| Farah, 2009 [223] | 0 | 50 | Arab world | Collaboration | Hybrid | Non-commercial | Infection (viral, sexually transmitted) |
| Hirani, 2012 [335] | 0 | 19 | Country-level | Inside | Open | Non-commercial | Infection (viral, sexually transmitted) |
| Youssef, 2008 [131] | 0 | 23 | Country-level | Inside | Non-Open | Non-commercial | Infection (viral, sexually transmitted) |
| Alshaikh, 2016 [25] | 0 | 7 | Country-level | Collaboration | Open | Commercial | Mental health |
| Buckle, 2012 [95] | 0 | 241 | Global | Outside | Open | Non-commercial | Trauma/Injury/Violence |
| Roberts, 2011 [132] | 0 | 13 | Global | Outside | Open | Non-commercial | Metabolic disease |
| Fares, 2011 [107] | 0 | 72 | Global | Outside | Open | Non-commercial | Neoplasm and genetics |
| Areeshi, 2014 [111] | 0 | 12 | GCC | Inside | Open | Commercial | Genetics and treatments |
| Araj, 2014 [124] | 0 | 4 | Country-level | Inside | Open | Non-commercial | Nutrition |
| Nasreddine, 2014 [40] | 0 | 12 | Country-level | Inside | Open | Non-commercial | Diabetes and genetics |
| Raja, 2008 [154] | 0 | 182 | Country-level | Collaboration | Open | Non-commercial | Trauma/Injury/Violence |
| Karageorgi, 2013 [266] | 0 | 22 | Country-level | Inside | Open | Commercial | Trauma/Injury/Violence |
| Ahmed, 2013 [284] | 0 | 19 | Country-level | Inside | Open | Non-commercial | Mental health |
| Bosan, 2010 [139] | 0 | 81 | Country-level | Collaboration | Open | Non-commercial | Infection (viral, respiratory) |
| Alsammani, 2016 [129] | 0 | 5 | Multiple regions | Inside | Non-Open | Non-commercial | Gastroenterology |
| Badawi, 2012 [250] | 0 | 23 | Country-level | Collaboration | Open | Commercial | Alcohol/Substance/Nicotine abuse |
| Hassen, 2012 [16] | 0 | 3 | Multiple regions | Outside | Non-Open | Non-commercial | Alcohol/Substance/Nicotine abuse |
| Alhyas, 2011 [269] | 0 | 98 | GCC | Outside | Open | Non-commercial | Congenital anomaly |
| Hassanien, 2012 [304] | 0 | 35 | GCC | Outside | Open | Non-commercial | Trauma/Injury/Violence |
| Rafeey, 2015 [369] | 0 | 3 | Global | Inside | Open | Non-commercial | Infection (viral, foodborne) |
| Fuhr, 2014 [245] | 0 | 23 | Global | Collaboration | Non-Open | Commercial | Infection (viral, foodborne) |
| Salhia, 2014 [227] | 0 | 10 | GCC | Collaboration | Open | Non-commercial | Infection (viral, foodborne) |
| John, 2015 [328] | 0 | 22 | Middle East | Inside | Open | Non-commercial | Infection (viral, foodborne) |
| Karoney, 2013 [162] | 0 | 97 | Africa | Inside | Open | Non-commercial | Cardiovascular disease and metabolic syndrome |
| Sabahelzain, 2014 [79] | 0 | 4 | Country-level | Collaboration | Open | Non-commercial | Trauma/Injury/Violence |
| Khader, 2015 [65] | 0 | 4 | MENA | Inside | Hybrid | Commercial | Trauma/Injury/Violence |
| Al Agili, 2013 [351] | 0 | 74 | Country-level | Inside | Open | Non-commercial | Infection (viral, bloodborne) |
| Aldrees, 2011 [20] | 0 | 9 | Country-level | Inside | Open | Non-commercial | Infection (viral, bloodborne) |
| BenNasir, 2015 [286] | 0 | 2 | North Africa | Collaboration | Open | Non-commercial | Infection (viral and parasitic, bloodborne and vector-borne) |
| Sabbagh, 2012 [21] | 0 | 17 | Country-level | Collaboration | Open | Non-commercial | Infection (viral, bloodborne) |
| Oluwagbemig, 2012 [291] | 0 | 20 | Africa | Inside | Open | Commercial | Cardiovascular disease and medical adherence |
| Majzoub, 2015 [381] | 0 | 5 | Multiple regions | Inside | Open | Non-commercial | Infection (bacterial) |
| Barakat, 2012 [36] | 0 | 15 | Country-level | Inside | Open | Non-commercial | Infection (viral, bloodborne) |
| Akeroyd, 2015 [34] | 0 | 4 | Asia | Outside | Open | Commercial | Neoplasm |
| Ashtari, 2015 [282] | 0 | 31 | Asia | Inside | Open | Commercial | Gastroenterology |
| Ashtari, 2015 [73] | 0 | 28 | Asia | Inside | Open | Commercial | Metabolic disease |
| [280] |  |  |  |  |  |  |  |

1 impact factor of the journal on the year when the systematic review was published

2 number of citations of the systematic review retrieved from Google Scholar on January 17, 2018

##

# Reference list of included studies

1. AlMarri TS, Oei TP. Alcohol and substance use in the Arabian Gulf region: a review. International journal of psychology : Journal international de psychologie. 2009;44(3):222-33. PubMed PMID: 22029498.

2. Popova S, Lange S, Probst C, Shield K, Kraicer-Melamed H, Ferreira-Borges C, et al. Actual and predicted prevalence of alcohol consumption during pregnancy in the WHO African Region. Tropical medicine & international health : TM & IH. 2016;21(10):1209-39. PubMed PMID: 27429168.

3. Ghandour L, Chalak A, El-Aily A, Yassin N, Nakkash R, Tauk M, et al. Alcohol consumption in the Arab region: What do we know, why does it matter, and what are the policy implications for youth harm reduction? The International journal on drug policy. 2016;28:10-33. PubMed PMID: 26547300.

4. Francis JM, Grosskurth H, Changalucha J, Kapiga SH, Weiss HA. Systematic review and meta-analysis: prevalence of alcohol use among young people in eastern Africa. Tropical medicine & international health : TM & IH. 2014;19(4):476-88. PubMed PMID: 24479379.

5. John LJ, Muttappallymyalil J. Dokha: an emerging public health issue as a form of tobacco smoking in the middle East. Asian Pacific journal of cancer prevention : APJCP. 2013;14(12):7065-7. PubMed PMID: 24460251.

6. Degenhardt L, Bucello C, Calabria B, Nelson P, Roberts A, Hall W, et al. What data are available on the extent of illicit drug use and dependence globally? Results of four systematic reviews. Drug Alcohol Depend. 2011;117(2):85-101. PubMed PMID: 21377813.

7. Degenhardt L, Charlson F, Mathers B, Hall WD, Flaxman AD, Johns N, et al. The global epidemiology and burden of opioid dependence: results from the global burden of disease 2010 study. Addiction. 2014;109(8):1320-33. PubMed PMID: 24661272.

8. Alsanosy RM. Smokeless tobacco (shammah) in Saudi Arabia: a review of its pattern of use, prevalence, and potential role in oral cancer. Asian Pacific journal of cancer prevention : APJCP. 2014;15(16):6477-83. PubMed PMID: 25169473.

9. Harbour C. Smoking and normative influence among Egyptian youth: a review of the literature. Eastern Mediterranean health journal = La revue de sante de la Mediterranee orientale = al-Majallah al-sihhiyah li-sharq al-mutawassit. 2011;17(4):349-55. PubMed PMID: 22259895.

10. Almutairi KM. Smoking among Saudi students: a review of risk factors and early intentions of smoking. J Community Health. 2014;39(5):901-7. PubMed PMID: 24984600.

11. Shah N, Siddiqui S. An overview of smoking practices in Pakistan. Pak J Med Sci. 2015;31(2):467-70. PubMed PMID: 26101513.

12. Abdullah AS, Stillman FA, Yang L, Luo H, Zhang Z, Samet JM. Tobacco use and smoking cessation practices among physicians in developing countries: a literature review (1987-2010). International journal of environmental research and public health. 2013;11(1):429-55. PubMed PMID: 24380976.

13. Kassim S, Jawad M, Croucher R, Akl EA. The Epidemiology of Tobacco Use among Khat Users: A Systematic Review. BioMed research international. 2015;2015:313692. PubMed PMID: 26273606.

14. Akl EA, Gunukula SK, Aleem S, Obeid R, Jaoude PA, Honeine R. The prevalence of waterpipe tobacco smoking among the general and specific populations: a systematic review. BMC Public Health. 2011;11. doi: 10.1186/1471-2458-11-244.

15. Akl EA, Jawad M, Lam WY, Co CN, Obeid R, Irani J. Motives, beliefs and attitudes towards waterpipe tobacco smoking: a systematic review. Harm reduction journal. 2013;10. doi: 10.1186/1477-7517-10-12.

16. Hassen K, Abdulahi M, Dejene T, Wolde M, Sudhakar M. Khat as a risk factor for hypertension: A systematic review. JBI library of systematic reviews. 2012;10(44):2882-905. PubMed PMID: 27820477.

17. El-Zaemey S, Schuz J, Leon ME. Qat Chewing and Risk of Potentially Malignant and Malignant Oral Disorders: A Systematic Review. The international journal of occupational and environmental medicine. 2015;6(3):129-43. PubMed PMID: 26174990.

18. Kakde S, Bhopal RS, Jones CM. A systematic review on the social context of smokeless tobacco use in the South Asian population: implications for public health. Public Health. 2012;126(8):635-45. PubMed PMID: 22809493.

19. Katelaris CH, Lee BW, Potter PC, Maspero JF, Cingi C, Lopatin A, et al. Prevalence and diversity of allergic rhinitis in regions of the world beyond Europe and North America. Clinical and experimental allergy : journal of the British Society for Allergy and Clinical Immunology. 2012;42(2):186-207. PubMed PMID: 22092947.

20. Aldrees AM. Lateral cephalometric norms for Saudi adults: A meta-analysis. The Saudi dental journal. 2011;23(1):3-7. PubMed PMID: 24151411.

21. Sabbagh HJ, Mossey PA, Innes NP. Prevalence of orofacial clefts in Saudi Arabia and neighboring countries: A systematic review. The Saudi dental journal. 2012;24(1):3-10. PubMed PMID: 23960521.

22. Yammine K. Clinical prevalence of palmaris longus agenesis: a systematic review and meta-analysis. Clinical anatomy (New York, NY). 2013;26(6):709-18. PubMed PMID: 23825029.

23. Ameh OI, Kengne AP, Jayne D, Bello AK, Hodkinson B, Gcelu A, et al. Standard of treatment and outcomes of adults with lupus nephritis in Africa: a systematic review. Lupus. 2016;25(11):1269-77. PubMed PMID: 27013662.

24. Lip GY, Brechin CM, Lane DA. The global burden of atrial fibrillation and stroke: a systematic review of the epidemiology of atrial fibrillation in regions outside North America and Europe. Chest. 2012;142(6):1489-98. PubMed PMID: 22459778.

25. Alshaikh MK, Filippidis FT, Baldove JP, Majeed A, Rawaf S. Women in Saudi Arabia and the Prevalence of Cardiovascular Risk Factors: A Systematic Review. J Environ Public Health. 2016;2016:7479357. PubMed PMID: 27777590.

26. Deek H, Newton P, Inglis S, Kabbani S, Noureddine S, Macdonald PS, et al. Heart health in Lebanon and considerations for addressing the burden of cardiovascular disease. Collegian. 2015;22(3):333-9. PubMed PMID: 26552205.

27. Benamer HT, Grosset D. Stroke in Arab countries: a systematic literature review. J Neurol Sci. 2009;284(1):18-23. PubMed PMID: 19428027.

28. Tran J, Mirzaei M, Anderson L, Leeder SR. The epidemiology of stroke in the Middle East and North Africa. J Neurol Sci. 2010;295(1):38-40. PubMed PMID: 20541222.

29. Behrouz R, Powers CJ. Epidemiology of classical risk factors in stroke patients in the Middle East. European journal of neurology : the official journal of the European Federation of Neurological Societies. 2016;23(2):262-9. PubMed PMID: 26041584.

30. Khan AA, Zafar SN. Venous thromboembolism in Pakistan: a neglected research agenda. Journal of thrombosis and thrombolysis. 2013;35(2):234-42. PubMed PMID: 22915351.

31. Yamamoto SS, Phalkey R, Malik AA. A systematic review of air pollution as a risk factor for cardiovascular disease in South Asia: limited evidence from India and Pakistan. International journal of hygiene and environmental health. 2014;217(2):133-44. PubMed PMID: 24064368.

32. Nasser Z, Salameh P, Nasser W, Abou Abbas L, Elias E, Leveque A. Outdoor particulate matter (PM) and associated cardiovascular diseases in the Middle East. International journal of occupational medicine and environmental health. 2015;28(4):641-61. PubMed PMID: 26216305.

33. Gonzalez JV, Barboza AG, Vazquez FJ, Gandara E. Prevalence and Geographical Variation of Prothrombin G20210A Mutation in Patients with Cerebral Vein Thrombosis: A Systematic Review and Meta-Analysis. PLoS One. 2016;11(3):e0151607. PubMed PMID: 27031503.

34. Akeroyd JM, Chan WJ, Kamal AK, Palaniappan L, Virani SS. Adherence to cardiovascular medications in the South Asian population: A systematic review of current evidence and future directions. World J Cardiol. 2015;7(12):938-47. PubMed PMID: 26730300.

35. Donnelly TT, Al Suwaidi JM, Al-Qahtani A, Asaad N, Qader NA, Singh R, et al. Depression in Cardiovascular Patients in Middle Eastern Populations: A Literature Review. Journal of immigrant and minority health / Center for Minority Public Health. 2015;17(4):1259-76. PubMed PMID: 24923859.

36. Barakat H, Barakat H, Baaj MK. CVD and obesity in transitional Syria: a perspective from the Middle East. Vascular health and risk management. 2012;8:145-50. PubMed PMID: 22454558.

37. Raza Q, Doak CM, Khan A, Nicolaou M, Seidell JC. Obesity and cardiovascular disease risk factors among the indigenous and immigrant Pakistani population: a systematic review. Obesity facts. 2013;6(6):523-35. PubMed PMID: 24296750.

38. Aljefree N, Ahmed F. Association between dietary pattern and risk of cardiovascular disease among adults in the Middle East and North Africa region: a systematic review. Food Nutr Res. 2015;59:27486. PubMed PMID: 26088003.

39. Mehio Sibai A, Nasreddine L, Mokdad AH, Adra N, Tabet M, Hwalla N. Nutrition transition and cardiovascular disease risk factors in Middle East and North Africa countries: reviewing the evidence. Ann Nutr Metab. 2010;57(3):193-203. PubMed PMID: 21088386.

40. Nasreddine L, Naja FA, Sibai AM, Helou K, Adra N, Hwalla N. Trends in nutritional intakes and nutrition-related cardiovascular disease risk factors in Lebanon: the need for immediate action. Le Journal medical libanais The Lebanese medical journal. 2014;62(2):83-91. PubMed PMID: 25011369.

41. Smythe T, Kuper H, Macleod D, Foster A, Lavy C. Birth prevalence of congenital talipes equinovarus in low- and middle-income countries: a systematic review and meta-analysis. Tropical medicine & international health : TM & IH. 2016;0. PubMed PMID: 28000394.

42. Vynnycky E, Adams EJ, Cutts FT, Reef SE, Navar AM, Simons E, et al. Using Seroprevalence and Immunisation Coverage Data to Estimate the Global Burden of Congenital Rubella Syndrome, 1996-2010: A Systematic Review. PLoS One. 2016;11(3):e0149160. PubMed PMID: 26962867.

43. Deckers IA, McLean S, Linssen S, Mommers M, van Schayck CP, Sheikh A. Investigating international time trends in the incidence and prevalence of atopic eczema 1990-2010: a systematic review of epidemiological studies. PLoS One. 2012;7(7):e39803. PubMed PMID: 22808063.

44. Jayawardena R, Ranasinghe P, Byrne NM, Soares MJ, Katulanda P, Hills AP. Prevalence and trends of the diabetes epidemic in South Asia: a systematic review and meta-analysis. BMC Public Health. 2012;12:380. PubMed PMID: 22630043.

45. Bos M, Agyemang C. Prevalence and complications of diabetes mellitus in Northern Africa, a systematic review. BMC Public Health. 2013;13:387. PubMed PMID: 23617762.

46. Zabetian A, Keli HM, Echouffo-Tcheugui JB, Narayan KM, Ali MK. Diabetes in the Middle East and North Africa. Diabetes Res Clin Pract. 2013;101(2):106-22. PubMed PMID: 23642969.

47. Lamri L, Gripiotis E, Ferrario A. Diabetes in Algeria and challenges for health policy: a literature review of prevalence, cost, management and outcomes of diabetes and its complications. Globalization and health. 2014;10:11. PubMed PMID: 24564974.

48. NCD Risk Factor Collaboration. Worldwide trends in diabetes since 1980: a pooled analysis of 751 population-based studies with 4.4 million participants. Lancet. 2016;387(10027):1513-30.

49. Mbanya V, Hussain A, Kengne AP. Application and applicability of non-invasive risk models for predicting undiagnosed prevalent diabetes in Africa: A systematic literature search. Primary care diabetes. 2015;9(5):317-29. PubMed PMID: 25975760.

50. Meo SA. Prevalence and future prediction of type 2 diabetes mellitus in the Kingdom of Saudi Arabia: A systematic review of published studies. JPMA The Journal of the Pakistan Medical Association. 2016;66(6):722-5. PubMed PMID: 27339576.

51. Zayed H. Epidemiology of diabetic ketoacidosis in Arab patients with type 1 diabetes: a systematic review. International journal of clinical practice. 2016;70(3):186-95. PubMed PMID: 26842462.

52. Usher-Smith JA, Thompson M, Ercole A, Walter FM. Variation between countries in the frequency of diabetic ketoacidosis at first presentation of type 1 diabetes in children: a systematic review. Diabetologia. 2012;55(11):2878-94. PubMed PMID: 22933123.

53. Danaei G, Finucane MM, Lu Y, Singh GM, Cowan MJ, Paciorek CJ, et al. National, regional, and global trends in fasting plasma glucose and diabetes prevalence since 1980: systematic analysis of health examination surveys and epidemiological studies with 370 country-years and 2.7 million participants. Lancet. 2011;378(9785):31-40. PubMed PMID: 21705069.

54. Alhyas L, McKay A, Majeed A. Prevalence of type 2 diabetes in the States of the co-operation council for the Arab States of the Gulf: a systematic review. PLoS One. 2012;7(8):e40948. Epub 2012/08/21. doi: 10.1371/journal.pone.0040948. PubMed PMID: 22905094; PubMed Central PMCID: PMCPMC3414510.

55. Meo SA, Zia I, Bukhari IA, Arain SA. Type 2 diabetes mellitus in Pakistan: Current prevalence and future forecast. JPMA The Journal of the Pakistan Medical Association. 2016;66(12):1637-42. PubMed PMID: 27924966.

56. Alkabab YM, Al-Abdely HM, Heysell SK. Diabetes-related tuberculosis in the Middle East: an urgent need for regional research. International journal of infectious diseases : IJID : official publication of the International Society for Infectious Diseases. 2015;40:64-70. PubMed PMID: 26409203.

57. Hassan A, Meo SA. Diabetes during Ramadan: underestimated, under-investigated, needs more attention. European review for medical and pharmacological sciences. 2014;18(22):3528-33. PubMed PMID: 25491633.

58. Yako YY, Guewo-Fokeng M, Balti EV, Bouatia-Naji N, Matsha TE, Sobngwi E, et al. Genetic risk of type 2 diabetes in populations of the African continent: A systematic review and meta-analyses. Diabetes Res Clin Pract. 2016;114:136-50. PubMed PMID: 26830076.

59. Hamzeh AR, Nair P, Al Ali MT. The profile of HLA-DRB1 alleles in Arabs with type 1 diabetes; meta-analyses. Hla. 2016;87(1):25-30. PubMed PMID: 26818122.

60. Hamzeh AR, Nair P, Al-Khaja N, Al Ali MT. Association of HLA-DQA1 and -DQB1 alleles with type I diabetes in Arabs: a meta-analyses. Tissue antigens. 2015;86(1):21-7. PubMed PMID: 26095634.

61. Alharbi NS, Almutari R, Jones S, Al-Daghri N, Khunti K, de Lusignan S. Trends in the prevalence of type 2 diabetes mellitus and obesity in the Arabian Gulf States: systematic review and meta-analysis. Diabetes Res Clin Pract. 2014;106(2):e30-3. PubMed PMID: 25241351.

62. Al-Khudairy L, Stranges S, Kumar S, Al-Daghri N, Rees K. Dietary factors and type 2 diabetes in the Middle East: what is the evidence for an association?--a systematic review. Nutrients. 2013;5(10):3871-97. PubMed PMID: 24077241.

63. Fleming E, Gillibrand W. An exploration of culture, diabetes, and nursing in the South Asian community: a metasynthesis of qualitative studies. Journal of transcultural nursing : official journal of the Transcultural Nursing Society / Transcultural Nursing Society. 2009;20(2):146-55.

64. Motlagh B, O'Donnell M, Yusuf S. Prevalence of cardiovascular risk factors in the Middle East: a systematic review. European journal of cardiovascular prevention and rehabilitation : official journal of the European Society of Cardiology, Working Groups on Epidemiology & Prevention and Cardiac Rehabilitation and Exercise Physiology. 2009;16(3):268-80. PubMed PMID: 19398913.

65. Khader YS, Abdelrahman M, Abdo N, Al-Sharif M, Elbetieha A, Bakir H, et al. Climate change and health in the Eastern Mediterranean countries: a systematic review. Reviews on environmental health. 2015;30(3):163-81. PubMed PMID: 26351799.

66. DeNicola E, Aburizaiza OS, Siddique A, Khwaja H, Carpenter DO. Climate Change and Water Scarcity: The Case of Saudi Arabia. Annals of global health. 2015;81(3):342-53.

67. Singh P, Arora S, Singh A, Strand TA, Makharia GK. Prevalence of celiac disease in Asia: A systematic review and meta-analysis. Journal of gastroenterology and hepatology. 2016;31(6):1095-101. PubMed PMID: 26678020.

68. Gautret P, Benkouiten S, Sridhar S, Al-Tawfiq JA, Memish ZA. Diarrhea at the Hajj and Umrah. Travel medicine and infectious disease. 2015;13(2):159-66. PubMed PMID: 25765485.

69. Olusanya BO, Osibanjo FB, Slusher TM. Risk factors for severe neonatal hyperbilirubinemia in low and middle-income countries: a systematic review and meta-analysis. PLoS One. 2015;10(2):e0117229. PubMed PMID: 25675342.

70. Molodecky NA, Soon IS, Rabi DM, Ghali WA, Ferris M, Chernoff G, et al. Increasing incidence and prevalence of the inflammatory bowel diseases with time, based on systematic review. Gastroenterology. 2012;142(1):46-54.e42; quiz e30. PubMed PMID: 22001864.

71. Devanarayana NM, Rajindrajith S, Pathmeswaran A, Abegunasekara C, Gunawardena NK, Benninga MA. Epidemiology of irritable bowel syndrome in children and adolescents in Asia. Journal of pediatric gastroenterology and nutrition. 2015;60(6):792-8. PubMed PMID: 26000888.

72. Sperber AD, Dumitrascu D, Fukudo S, Gerson C, Ghoshal UC, Gwee KA, et al. The global prevalence of IBS in adults remains elusive due to the heterogeneity of studies: a Rome Foundation working team literature review. Gut. 2016;0. PubMed PMID: 26818616.

73. Ashtari S, Pourhoseingholi MA, Zali MR. Non-alcohol fatty liver disease in Asia: Prevention and planning. World journal of hepatology. 2015;7(13):1788-96. PubMed PMID: 26167252.

74. Jiang J, Jiang B, Parashar U, Nguyen T, Bines J, Patel MM. Childhood intussusception: a literature review. PLoS One. 2013;8(7):e68482. PubMed PMID: 23894308.

75. de Menthon M, Lavalley MP, Maldini C, Guillevin L, Mahr A. HLA-B51/B5 and the risk of Behcet's disease: a systematic review and meta-analysis of case-control genetic association studies. Arthritis and rheumatism. 2009;61(10):1287-96. PubMed PMID: 19790126.

76. Romdhane L, Kefi R, Azaiez H, Ben Halim N, Dellagi K, Abdelhak S. Founder mutations in Tunisia: implications for diagnosis in North Africa and Middle East. Orphanet journal of rare diseases. 2012;7:52. PubMed PMID: 22908982.

77. Doss CG, Alasmar DR, Bux RI, Sneha P, Bakhsh FD, Al-Azwani I, et al. Genetic Epidemiology of Glucose-6-Dehydrogenase Deficiency in the Arab World. Scientific reports. 2016;6:37284. PubMed PMID: 27853304.

78. Romdhane L, Abdelhak S. Genetic diseases in the Tunisian population. American journal of medical genetics Part A. 2011;155(1):238-67. PubMed PMID: 21204241.

79. Sabahelzain MM, Hamamy H. The ethnic distribution of sickle cell disease in Sudan. The Pan African medical journal. 2014;18:13. PubMed PMID: 25360197.

80. Habeb AM. Frequency and spectrum of Wolcott-Rallison syndrome in Saudi Arabia: a systematic review. The Libyan journal of medicine. 2013;8:21137. PubMed PMID: 23759358.

81. Mahdieh N, Rabbani B. Beta thalassemia in 31,734 cases with HBB gene mutations: Pathogenic and structural analysis of the common mutations; Iran as the crossroads of the Middle East. Blood reviews. 2016;30(6):493-508. PubMed PMID: 27453201.

82. Bader LA, Elewa H. The Impact of Genetic and Non-Genetic Factors on Warfarin Dose Prediction in MENA Region: A Systematic Review. PLoS One. 2016;11(12):e0168732. PubMed PMID: 27992547.

83. Gaikwad T, Ghosh K, Shetty S. VKORC1 and CYP2C9 genotype distribution in Asian countries. Thrombosis research. 2014;134(3):537-44. PubMed PMID: 24908449.

84. Inhorn MC, Patrizio P. Infertility around the globe: new thinking on gender, reproductive technologies and global movements in the 21st century. Human reproduction update. 2015;21(4):411-26. PubMed PMID: 25801630.

85. Mandil A, Chaaya M, Saab D. Health status, epidemiological profile and prospects: Eastern Mediterranean Region. International journal of epidemiology. 2013;42(2):616-26. PubMed PMID: 23505252.

86. Tajvar M, Fletcher A, Grundy E, Arab M. Social support and health of older people in Middle Eastern countries: a systematic review. Australas J Ageing. 2013;32(2):71-8. PubMed PMID: 23773244.

87. Benova L, Campbell OM, Ploubidis GB. Socio-economic gradients in maternal and child health-seeking behaviours in egypt: systematic literature review and evidence synthesis. PLoS One. 2014;9(3):e93032. PubMed PMID: 24663341.

88. Salmon-Rousseau A, Piednoir E, Cattoir V, de La Blanchardiere A. Hajj-associated infections. Medecine et maladies infectieuses. 2016;46(7):346-54. PubMed PMID: 27230822.

89. Tosson AM, Speer CP. Microbial pathogens causative of neonatal sepsis in Arabic countries. The journal of maternal-fetal & neonatal medicine : the official journal of the European Association of Perinatal Medicine, the Federation of Asia and Oceania Perinatal Societies, the International Society of Perinatal Obstet. 2011;24(8):990-4. PubMed PMID: 21668322.

90. Gautret P, Steffen R. Communicable diseases as health risks at mass gatherings other than Hajj: what is the evidence? International journal of infectious diseases : IJID : official publication of the International Society for Infectious Diseases. 2016;47:46-52. PubMed PMID: 26987476.

91. Prasad N, Murdoch DR, Reyburn H, Crump JA. Etiology of Severe Febrile Illness in Low- and Middle-Income Countries: A Systematic Review. PLoS One. 2015;10(6):e0127962. PubMed PMID: 26126200.

92. DeAntonio R, Yarzabal JP, Cruz JP, Schmidt JE, Kleijnen J. Epidemiology of otitis media in children from developing countries: A systematic review. International journal of pediatric otorhinolaryngology. 2016;85:65-74. PubMed PMID: 27240499.

93. Musallam, II, Abo-Shehada MN, Hegazy YM, Holt HR, Guitian FJ. Systematic review of brucellosis in the Middle East: disease frequency in ruminants and humans and risk factors for human infection. Epidemiology and infection. 2016;144(4):671-85. PubMed PMID: 26508323.

94. Naseer M, Jamali T. Epidemiology, determinants and dynamics of cholera in Pakistan: gaps and prospects for future research. Journal of the College of Physicians and Surgeons--Pakistan : JCPSP. 2014;24(11):855-60.

95. Buckle GC, Walker CL, Black RE. Typhoid fever and paratyphoid fever: Systematic review to estimate global morbidity and mortality for 2010. Journal of global health. 2012;2(1):010401. PubMed PMID: 23198130.

96. Azmatullah A, Qamar FN, Thaver D, Zaidi AK, Bhutta ZA. Systematic review of the global epidemiology, clinical and laboratory profile of enteric fever. Journal of global health. 2015;5(2):020407. PubMed PMID: 26649174.

97. Coffin LS, Newberry A, Hagan H, Cleland CM, Des Jarlais DC, Perlman DC. Syphilis in drug users in low and middle income countries. The International journal on drug policy. 2010;21(1):20-7. PubMed PMID: 19361976.

98. Kenyon C, Colebunders R, Crucitti T. The global epidemiology of bacterial vaginosis: a systematic review. Am J Obstet Gynecol. 2013;209(6):505-23. PubMed PMID: 23659989.

99. Manenzhe RI, Zar HJ, Nicol MP, Kaba M. The spread of carbapenemase-producing bacteria in Africa: a systematic review. The Journal of antimicrobial chemotherapy. 2015;70(1):23-40. PubMed PMID: 25261423.

100. Vanderburg S, Rubach MP, Halliday JE, Cleaveland S, Reddy EA, Crump JA. Epidemiology of Coxiella burnetii infection in Africa: a OneHealth systematic review. PLoS neglected tropical diseases. 2014;8(4):e2787. PubMed PMID: 24722554.

101. Steer AC, Law I, Matatolu L, Beall BW, Carapetis JR. Global emm type distribution of group A streptococci: systematic review and implications for vaccine development. The Lancet Infectious diseases. 2009;9(10):611-6. PubMed PMID: 19778763.

102. Khedmat H, Karbasi-Afshar R, Agah S, Taheri S. Helicobacter pylori Infection in the general population: A Middle Eastern perspective. Caspian J Intern Med. 2013;4(4):745-53. PubMed PMID: 24294467.

103. Eshraghian A. Epidemiology of Helicobacter pylori infection among the healthy population in Iran and countries of the Eastern Mediterranean Region: a systematic review of prevalence and risk factors. World journal of gastroenterology : WJG. 2014;20(46):17618-25. PubMed PMID: 25516677.

104. Dean AS, Crump L, Greter H, Schelling E, Zinsstag J. Global burden of human brucellosis: a systematic review of disease frequency. PLoS neglected tropical diseases. 2012;6(10):e1865. PubMed PMID: 23145195.

105. Shibl A, Memish Z, Pelton S. Epidemiology of invasive pneumococcal disease in the Arabian Peninsula and Egypt. International journal of antimicrobial agents. 2009;33(5):410.e1-9. PubMed PMID: 18976887.

106. Hung IF, Tantawichien T, Tsai YH, Patil S, Zotomayor R. Regional epidemiology of invasive pneumococcal disease in Asian adults: epidemiology, disease burden, serotype distribution, and antimicrobial resistance patterns and prevention. International journal of infectious diseases : IJID : official publication of the International Society for Infectious Diseases. 2013;17(6):e364-73. PubMed PMID: 23416209.

107. Fares A. Seasonality of tuberculosis. Journal of global infectious diseases. 2011;3(1):46-55. PubMed PMID: 21572609.

108. Horton KC, MacPherson P, Houben RM, White RG, Corbett EL. Sex Differences in Tuberculosis Burden and Notifications in Low- and Middle-Income Countries: A Systematic Review and Meta-analysis. PLoS medicine. 2016;13(9):e1002119. PubMed PMID: 27598345.

109. Ghenghesh KS, Rahouma A, Zorgani A, Tawil K, Al Tomi A, Franka E. Aeromonas in Arab countries: 1995-2014. Comp Immunol Microbiol Infect Dis. 2015;42:8-14. PubMed PMID: 26577192.

110. Tansarli GS, Poulikakos P, Kapaskelis A, Falagas ME. Proportion of extended-spectrum beta-lactamase (ESBL)-producing isolates among Enterobacteriaceae in Africa: evaluation of the evidence--systematic review. The Journal of antimicrobial chemotherapy. 2014;69(5):1177-84. PubMed PMID: 24398340.

111. Areeshi MY, Bisht SC, Mandal RK, Haque S. Prevalence of drug resistance in clinical isolates of tuberculosis from GCC: a literature review from January 2002 to March 2013. Journal of infection in developing countries. 2014;8(9):1137-47. PubMed PMID: 25212078.

112. Sangare SA, Maiga AI, Guindo I, Maiga A, Camara N, Savadogo S, et al. Prevalence of extended-spectrum beta-lactamase-producing Enterobacteriaceae isolated from blood cultures in Africa. Medecine et maladies infectieuses. 2015;45(9):374-82. PubMed PMID: 26433872.

113. Abdulgader SM, Shittu AO, Nicol MP, Kaba M. Molecular epidemiology of Methicillin-resistant Staphylococcus aureus in Africa: a systematic review. Frontiers in microbiology. 2015;6:348. PubMed PMID: 25983721.

114. Mamishi S, Moradkhani S, Mahmoudi S, Hosseinpour-Sadeghi R, Pourakbari B. Penicillin-Resistant trend of Streptococcus pneumoniae in Asia: A systematic review. Iran J Microbiol. 2014;6(4):198-210. PubMed PMID: 25802701.

115. Jaiswal N, Singh M, Das RR, Jindal I, Agarwal A, Thumburu KK, et al. Distribution of serotypes, vaccine coverage, and antimicrobial susceptibility pattern of Streptococcus pneumoniae in children living in SAARC countries: a systematic review. PLoS One. 2014;9(9):e108617. PubMed PMID: 25268974.

116. Gu B, Zhou M, Ke X, Pan S, Cao Y, Huang Y, et al. Comparison of resistance to third-generation cephalosporins in Shigella between Europe-America and Asia-Africa from 1998 to 2012. Epidemiology and infection. 2015;143(13):2687-99. PubMed PMID: 25553947.

117. Kahsay AG, Muthupandian S. A review on Sero diversity and antimicrobial resistance patterns of Shigella species in Africa, Asia and South America, 2001-2014. BMC research notes. 2016;9(1):422. PubMed PMID: 27576729.

118. Berrazeg M, Diene S, Medjahed L, Parola P, Drissi M, Raoult D, et al. New Delhi Metallo-beta-lactamase around the world: an eReview using Google Maps. Euro Surveill. 2014;19(20). PubMed PMID: 24871756.

119. Velayati AA, Rahideh S, Nezhad ZD, Farnia P, Mirsaeidi M. Nontuberculous mycobacteria in Middle East: Current situation and future challenges. Int J Mycobacteriol. 2015;4(1):7-17. PubMed PMID: 26655192.

120. Al-Tawfiq JA, Hinedi K, Memish ZA. Systematic review of the prevalence of Mycobacterium tuberculosis resistance in Saudi Arabia. Journal of chemotherapy (Florence, Italy). 2015;27(6):378-82. PubMed PMID: 26179987.

121. Chakrabarti A, Chatterjee SS, Das A, Shivaprakash MR. Invasive aspergillosis in developing countries. Medical mycology. 2011;49:S35-47. PubMed PMID: 20718613.

122. van de Sande WW. Global burden of human mycetoma: a systematic review and meta-analysis. PLoS neglected tropical diseases. 2013;7(11):e2550. PubMed PMID: 24244780.

123. Romani L, Steer AC, Whitfeld MJ, Kaldor JM. Prevalence of scabies and impetigo worldwide: a systematic review. The Lancet Infectious diseases. 2015;15(8):960-7. PubMed PMID: 26088526.

124. Araj GF, Mourad Y. Hydatid disease: the Lebanese contribution. Le Journal medical libanais The Lebanese medical journal. 2014;62(4):217-26. PubMed PMID: 25807720.

125. D'Acremont V, Lengeler C, Genton B. Reduction in the proportion of fevers associated with Plasmodium falciparum parasitaemia in Africa: a systematic review. Malaria journal. 2010;9:240. PubMed PMID: 20727214.

126. Rahimi BA, Thakkinstian A, White NJ, Sirivichayakul C, Dondorp AM, Chokejindachai W. Severe vivax malaria: a systematic review and meta-analysis of clinical studies since 1900. Malaria journal. 2014;13:481. PubMed PMID: 25486908.

127. Coleman M, Al-Zahrani MH, Coleman M, Hemingway J, Omar A, Stanton MC, et al. A country on the verge of malaria elimination--the Kingdom of Saudi Arabia. PLoS One. 2014;9(9):e105980. PubMed PMID: 25250619.

128. Pappas G, Roussos N, Falagas ME. Toxoplasmosis snapshots: global status of Toxoplasma gondii seroprevalence and implications for pregnancy and congenital toxoplasmosis. Int J Parasitol. 2009;39(12):1385-94. PubMed PMID: 19433092.

129. Alsammani MA. Sero-epidemiology and risk factors for Toxoplasma gondii among pregnant women in Arab and African countries. Journal of parasitic diseases : official organ of the Indian Society for Parasitology. 2016;40(3):569-79. PubMed PMID: 27605750.

130. Al-Salem W, Herricks JR, Hotez PJ. A review of visceral leishmaniasis during the conflict in South Sudan and the consequences for East African countries. Parasites & vectors. 2016;9:460. PubMed PMID: 27549162.

131. Youssef FG, Adib I, Riddle MS, Schlett CD. A review of cryptosporidiosis in Egypt. Journal of the Egyptian Society of Parasitology. 2008;38(1):9-28. PubMed PMID: 19143117.

132. Roberts T, Gravett CA, Velu PP, Theodoratou E, Wagner TA, Zhang JS, et al. Epidemiology and aetiology of maternal parasitic infections in low- and middle-income countries. Journal of global health. 2011;1(2):189-200. PubMed PMID: 23198118.

133. Chanda E, Govere JM, Macdonald MB, Lako RL, Haque U, Baba SP, et al. Integrated vector management: a critical strategy for combating vector-borne diseases in South Sudan. Malaria journal. 2013;12:369. PubMed PMID: 24156749.

134. Ansarie M, Kasmani A. Community acquired pneumonia in Pakistan: an analysis on the literature published between 2003 and 2013. JPMA The Journal of the Pakistan Medical Association. 2014;64(12):1405-9. PubMed PMID: 25842587.

135. DeAntonio R, Yarzabal JP, Cruz JP, Schmidt JE, Kleijnen J. Epidemiology of community-acquired pneumonia and implications for vaccination of children living in developing and newly industrialized countries: A systematic literature review. Hum Vaccin Immunother. 2016;12(9):2422-40. PubMed PMID: 27269963.

136. Alqahtani AS, Rashid H, Heywood AE. Vaccinations against respiratory tract infections at Hajj. Clinical microbiology and infection : the official publication of the European Society of Clinical Microbiology and Infectious Diseases. 2015;21(2):115-27. PubMed PMID: 25682277.

137. Gasim GI, Bella A, Adam I. Schistosomiasis, hepatitis B and hepatitis C co-infection. Virology journal. 2015;12(1):19.

138. Van-Lume DSdM, de Albuquerque MdFP, de Souza AI, Domingues ALC, Lopes EPdA, de Morais CNL, et al. Association between Schistosomiasis mansoni and hepatitis C: systematic review. Revista de Saúde Pública. 2013;47(2):414-24.

139. Bosan A, Qureshi H, Bile KM, Ahmad I, Hafiz R. A review of hepatitis viral infections in Pakistan. JPMA-Journal of the Pakistan Medical Association. 2010;60(12):1045.

140. Azevedo TCL, Zwahlen M, Rauch A, Egger M, Wandeler G. Hepatitis C in HIV-infected individuals: a systematic review and meta-analysis of estimated prevalence in Africa. Journal of the International AIDS Society. 2016;19(1).

141. Schweitzer A, Horn J, Mikolajczyk RT, Krause G, Ott JJ. Estimations of worldwide prevalence of chronic hepatitis B virus infection: a systematic review of data published between 1965 and 2013. Lancet. 2015;386(10003):1546-55. PubMed PMID: 26231459.

142. Ali M, Idrees M, Ali L, Hussain A, Rehman IU, Saleem S, et al. Hepatitis B virus in Pakistan: a systematic review of prevalence, risk factors, awareness status and genotypes. Virology journal. 2011;8(1):102.

143. Ott JJ, Stevens GA, Wiersma ST. The risk of perinatal hepatitis B virus transmission: hepatitis B e antigen (HBeAg) prevalence estimates for all world regions. BMC infectious diseases. 2012;12(1):131.

144. Ezzikouri S, Pineau P, Benjelloun S. Hepatitis B virus in the Maghreb region: from epidemiology to prospective research. Liver international : official journal of the International Association for the Study of the Liver. 2013;33(6):811-9. PubMed PMID: 23530901.

145. Gasim GI. Hepatitis B virus in the Arab world: where do we stand? Arab Journal of Gastroenterology. 2013;14(2):35-43.

146. Babanejad M, Izadi N, Najafi F, Alavian SM. The HBsAg Prevalence Among Blood Donors From Eastern Mediterranean and Middle Eastern Countries: A Systematic Review and Meta-Analysis. Hepatitis monthly. 2016;16(3):e35664. PubMed PMID: 27226804.

147. Bajubair MA, Elrub AA, Bather G. Hepatic viral infections in Yemen between 2000-2005. Saudi Medical Journal. 2008;29(6):871.

148. Ali SA, Donahue RM, Qureshi H, Vermund SH. Hepatitis B and hepatitis C in Pakistan: prevalence and risk factors. International journal of infectious diseases : IJID : official publication of the International Society for Infectious Diseases. 2009;13(1):9-19. PubMed PMID: 18835208.

149. Lehman EM, Wilson ML. Epidemiology of hepatitis viruses among hepatocellular carcinoma cases and healthy people in Egypt: A systematic review and meta‐analysis. International journal of cancer. 2009;124(3):690-7.

150. Nelson PK, Mathers BM, Cowie B, Hagan H, Des Jarlais D, Horyniak D, et al. Global epidemiology of hepatitis B and hepatitis C in people who inject drugs: results of systematic reviews. Lancet. 2011;378(9791):571-83. PubMed PMID: 21802134.

151. Gasim GI, Murad IA, Adam I. Hepatitis B and C virus infections among pregnant women in Arab and African countries. Journal of infection in developing countries. 2013;7(8):566-78. PubMed PMID: 23949291.

152. Bashour H, Muhjazi G. Hepatitis B and C in the Syrian Arab Republic: a review/Hepatites B et C en Republique arabe syrienne: analyse. Eastern Mediterranean Health Journal. 2016;22(4):267.

153. Riaz M, Idrees M, Kanwal H, Kabir F. An overview of triple infection with hepatitis B, C and D viruses. Virology journal. 2011;8:368. PubMed PMID: 21791115.

154. Raja NS, Janjua KA. Epidemiology of hepatitis C virus infection in Pakistan. Journal of Microbiology Immunology and Infection. 2008;41(1):4.

155. Stern RK, Hagan H, Lelutiu-Weinberger C, Des Jarlais D, Scheinmann R, Strauss S, et al. The HCV Synthesis Project: scope, methodology, and preliminary results. BMC Med Res Methodol. 2008;8:62. PubMed PMID: 18789163.

156. Umar M, Khaar H, Khurram M, Hasan Z. Anti-HCV antibody positivity of various sections of Pakistani patients. Journal of the College of Physicians and Surgeons--Pakistan : JCPSP. 2009;19(11):737-41.

157. Waheed Y, Shafi T, Safi SZ, Qadri I. Hepatitis C virus in Pakistan: a systematic review of prevalence, genotypes and risk factors. World journal of gastroenterology : WJG. 2009;15(45):5647-53. PubMed PMID: 19960560.

158. Attaullah S, Khan S, Ali I. Hepatitis C virus genotypes in Pakistan: a systemic review. Virology journal. 2011;8:433. PubMed PMID: 21902822.

159. Sievert W, Altraif I, Razavi HA, Abdo A, Ahmed EA, AlOmair A, et al. A systematic review of hepatitis C virus epidemiology in Asia, Australia and Egypt. Liver International. 2011;31(s2):61-80.

160. Alavian SM, Aalaei-Andabili SH. Lack of Knowledge About Hepatitis C Infection Rates Among Patients With Inherited Coagulation Disorders in Countries Under the Eastern Mediterranean Region Office of WHO (EMRO): A Meta-Analysis. Hepatitis monthly. 2012;12(4):244-52. PubMed PMID: 22690231.

161. Ramia S, Melhem NM, Kreidieh K. Hepatitis C virus infection in the Middle East and North Africa "MENA" region: injecting drug users (IDUs) is an under-investigated population. Infection. 2012;40(1):1-10. PubMed PMID: 22237470.

162. Karoney MJ, Siika AM. Hepatitis C virus (HCV) infection in Africa: a review. The Pan African medical journal. 2013;14:44. PubMed PMID: 23560127.

163. Abozaid SM, Shoukri M, Al-Qahtani A, Al-Ahdal MN. Prevailing genotypes of hepatitis C virus in Saudi Arabia: a systematic analysis of evidence. Ann Saudi Med. 2013;33(1):1-5. PubMed PMID: 23458931.

164. Ezzikouri S, Pineau P, Benjelloun S. Hepatitis C virus infection in the Maghreb region. Journal of medical virology. 2013;85(9):1542-9. PubMed PMID: 23780703.

165. Mohd Hanafiah K, Groeger J, Flaxman AD, Wiersma ST. Global epidemiology of hepatitis C virus infection: new estimates of age-specific antibody to HCV seroprevalence. Hepatology. 2013;57(4):1333-42. PubMed PMID: 23172780.

166. Mohamoud YA, Mumtaz GR, Riome S, Miller D, Abu-Raddad LJ. The epidemiology of hepatitis C virus in Egypt: a systematic review and data synthesis. BMC infectious diseases. 2013;13:288. PubMed PMID: 23799878.

167. Reker C, Islam KM. Risk factors associated with high prevalence rates of hepatitis C infection in Egypt. International journal of infectious diseases : IJID : official publication of the International Society for Infectious Diseases. 2014;25:104-6. PubMed PMID: 24865321.

168. Bruggmann P, Berg T, Ovrehus AL, Moreno C, Brandao Mello CE, Roudot-Thoraval F, et al. Historical epidemiology of hepatitis C virus (HCV) in selected countries. Journal of viral hepatitis. 2014;21:5-33. PubMed PMID: 24713004.

169. Gower E, Estes C, Blach S, Razavi-Shearer K, Razavi H. Global epidemiology and genotype distribution of the hepatitis C virus infection. Journal of hepatology. 2014;61(1):S45-57. PubMed PMID: 25086286.

170. El-Ghitany EM, Abdel Wahab MM, Abd El-Wahab EW, Hassouna S, Farghaly AG. A comprehensive hepatitis C virus risk factors meta-analysis (1989-2013): do they differ in Egypt? Liver international : official journal of the International Association for the Study of the Liver. 2015;35(2):489-501. PubMed PMID: 24923487.

171. Chemaitelly H, Chaabna K, Abu-Raddad LJ. The Epidemiology of Hepatitis C Virus in the Fertile Crescent: Systematic Review and Meta-Analysis. PLoS One. 2015;10(8):e0135281. PubMed PMID: 26296200.

172. Fadlalla FA, Mohamoud YA, Mumtaz GR, Abu-Raddad LJ. The epidemiology of hepatitis C virus in the Maghreb region: systematic review and meta-analyses. PLoS One. 2015;10(3):e0121873. PubMed PMID: 25803848.

173. Riou J, Ait Ahmed M, Blake A, Vozlinsky S, Brichler S, Eholie S, et al. Hepatitis C virus seroprevalence in adults in Africa: a systematic review and meta-analysis. Journal of viral hepatitis. 2016;23(4):244-55. PubMed PMID: 26477881.

174. Ghaderi-Zefrehi H, Gholami-Fesharaki M, Sharafi H, Sadeghi F, Alavian SM. The Distribution of Hepatitis C Virus Genotypes in Middle Eastern Countries: A Systematic Review and Meta-Analysis. Hepatitis monthly. 2016;16(9):e40357. PubMed PMID: 27826320.

175. Sadeghi F, Salehi-Vaziri M, Almasi-Hashiani A, Gholami-Fesharaki M, Pakzad R, Alavian SM. Prevalence of Hepatitis C Virus Genotypes Among Patients in Countries of the Eastern Mediterranean Regional Office of WHO (EMRO): A Systematic Review and Meta-Analysis. Hepatitis monthly. 2016;16(4):e35558. PubMed PMID: 27274353.

176. Chaabna K, Kouyoumjian SP, Abu-Raddad LJ. Hepatitis C Virus Epidemiology in Djibouti, Somalia, Sudan, and Yemen: Systematic Review and Meta-Analysis. PLoS One. 2016;11(2):e0149966. PubMed PMID: 26900839.

177. Mohamoud YA, Riome S, Abu-Raddad LJ. Epidemiology of hepatitis C virus in the Arabian Gulf countries: Systematic review and meta-analysis of prevalence. International journal of infectious diseases : IJID : official publication of the International Society for Infectious Diseases. 2016;46:116-25. PubMed PMID: 26996460.

178. Amini N, Alavian SM, Kabir A, Aalaei-Andabili SH, Saiedi Hosseini SY, Rizzetto M. Prevalence of hepatitis d in the eastern mediterranean region: systematic review and meta analysis. Hepatitis monthly. 2013;13(1):e8210. PubMed PMID: 23554822.

179. Alavian SM, Haghbin H. Relative Importance of Hepatitis B and C Viruses in Hepatocellular Carcinoma in EMRO Countries and the Middle East: A Systematic Review. Hepatitis monthly. 2016;16(3):e35106. PubMed PMID: 27226803.

180. de Martel C, Maucort-Boulch D, Plummer M, Franceschi S. World-wide relative contribution of hepatitis B and C viruses in hepatocellular carcinoma. Hepatology. 2015;62(4):1190-200. PubMed PMID: 26146815.

181. Jacobsen KH, Wiersma ST. Hepatitis A virus seroprevalence by age and world region, 1990 and 2005. Vaccine. 2010;28(41):6653-7. PubMed PMID: 20723630.

182. Itani T, Jacobsen KH, Nguyen T, Wiktor SZ. A new method for imputing country-level estimates of hepatitis A virus endemicity levels in the Eastern Mediterranean region. Vaccine. 2014;32(46):6067-74. PubMed PMID: 25236586.

183. Yazbek S, Kreidieh K, Ramia S. Hepatitis E virus in the countries of the Middle East and North Africa region: an awareness of an infectious threat to blood safety. Infection. 2016;44(1):11-22. PubMed PMID: 26112744.

184. Parashar UD, Burton A, Lanata C, Boschi-Pinto C, Shibuya K, Steele D, et al. Global mortality associated with rotavirus disease among children in 2004. The Journal of infectious diseases. 2009;200:S9-s15. PubMed PMID: 19817620.

185. Khoury H, Ogilvie I, El Khoury AC, Duan Y, Goetghebeur MM. Burden of rotavirus gastroenteritis in the Middle Eastern and North African pediatric population. BMC infectious diseases. 2011;11:9. PubMed PMID: 21214934.

186. Miles MG, Lewis KD, Kang G, Parashar UD, Steele AD. A systematic review of rotavirus strain diversity in India, Bangladesh, and Pakistan. Vaccine. 2012;30:A131-9. PubMed PMID: 22520122.

187. Kawai K, O'Brien MA, Goveia MG, Mast TC, El Khoury AC. Burden of rotavirus gastroenteritis and distribution of rotavirus strains in Asia: a systematic review. Vaccine. 2012;30(7):1244-54. PubMed PMID: 22212128.

188. Tate JE, Burton AH, Boschi-Pinto C, Steele AD, Duque J, Parashar UD. 2008 estimate of worldwide rotavirus-associated mortality in children younger than 5 years before the introduction of universal rotavirus vaccination programmes: a systematic review and meta-analysis. The Lancet Infectious diseases. 2012;12(2):136-41. PubMed PMID: 22030330.

189. Jroundi I, Mahraoui C, Benmessaoud R, Moraleda C, Benjelloun B, Bassat Q. Knowledge gaps on paediatric respiratory infections in Morocco, Northern Africa. Arch Public Health. 2015;73(1):28. PubMed PMID: 26078866.

190. Abubakar A, Malik M, Pebody RG, Elkholy AA, Khan W, Bellos A, et al. Burden of acute respiratory disease of epidemic and pandemic potential in the WHO Eastern Mediterranean Region: A literature review. Eastern Mediterranean health journal = La revue de sante de la Mediterranee orientale = al-Majallah al-sihhiyah li-sharq al-mutawassit. 2016;22(7):513-26. PubMed PMID: 27714746.

191. Mathur MB, Patel RB, Gould M, Uyeki TM, Bhattacharya J, Xiao Y, et al. Seasonal patterns in human A (H5N1) virus infection: analysis of global cases. PLoS One. 2014;9(9):e106171. PubMed PMID: 25215608.

192. Lai S, Qin Y, Cowling BJ, Ren X, Wardrop NA, Gilbert M, et al. Global epidemiology of avian influenza A H5N1 virus infection in humans, 1997-2015: a systematic review of individual case data. The Lancet Infectious diseases. 2016;16(7):e108-18. PubMed PMID: 27211899.

193. Tricco AC, Lillie E, Soobiah C, Perrier L, Straus SE. Impact of H1N1 on socially disadvantaged populations: summary of a systematic review. Influenza and other respiratory viruses. 2013;7:54-8. PubMed PMID: 24034485.

194. Alsolamy S. Middle East respiratory syndrome: knowledge to date. Critical care medicine. 2015;43(6):1283-90. PubMed PMID: 25785521.

195. Gautret P, Benkouiten S, Al-Tawfiq JA, Memish ZA. Hajj-associated viral respiratory infections: A systematic review. Travel medicine and infectious disease. 2016;14(2):92-109. PubMed PMID: 26781223.

196. Mathers BM, Degenhardt L, Phillips B, Wiessing L, Hickman M, Strathdee SA, et al. Global epidemiology of injecting drug use and HIV among people who inject drugs: a systematic review. Lancet. 2008;372(9651):1733-45. PubMed PMID: 18817968.

197. Mathers BM, Degenhardt L, Ali H, Wiessing L, Hickman M, Mattick RP, et al. HIV prevention, treatment, and care services for people who inject drugs: a systematic review of global, regional, and national coverage. Lancet. 2010;375(9719):1014-28. PubMed PMID: 20189638.

198. Abu-Raddad LJ, Hilmi N, Mumtaz G, Benkirane M, Akala FA, Riedner G, et al. Epidemiology of HIV infection in the Middle East and North Africa. Aids. 2010;24:S5-23. PubMed PMID: 20610949.

199. Mumtaz G, Hilmi N, McFarland W, Kaplan RL, Akala FA, Semini I, et al. Are HIV epidemics among men who have sex with men emerging in the Middle East and North Africa?: a systematic review and data synthesis. PLoS medicine. 2010;8(8):e1000444. PubMed PMID: 21829329.

200. Mumtaz G, Hilmi N, Akala FA, Semini I, Riedner G, Wilson D, et al. HIV-1 molecular epidemiology evidence and transmission patterns in the Middle East and North Africa. Sexually transmitted infections. 2011;87(2):101-6.

201. Kouyoumjian SP, Mumtaz GR, Hilmi N, Zidouh A, El Rhilani H, Alami K, et al. The epidemiology of HIV infection in Morocco: systematic review and data synthesis. International journal of STD & AIDS. 2013;24(7):507-16. PubMed PMID: 23970764.

202. Mumtaz GR, Kouyoumjian SP, Hilmi N, Zidouh A, El Rhilani H, Alami K, et al. The distribution of new HIV infections by mode of exposure in Morocco. Sexually transmitted infections. 2013;89:iii49-56. PubMed PMID: 23413401.

203. Singh S, Ambrosio M, Semini I, Tawil O, Saleem M, Imran M, et al. Revitalizing the HIV response in Pakistan: a systematic review and policy implications. The International journal on drug policy. 2014;25(1):26-33. PubMed PMID: 23810289.

204. Mumtaz GR, Weiss HA, Thomas SL, Riome S, Setayesh H, Riedner G, et al. HIV among people who inject drugs in the Middle East and North Africa: systematic review and data synthesis. PLoS medicine. 2014;11(6):e1001663. PubMed PMID: 24937136.

205. Poteat T, Scheim A, Xavier J, Reisner S, Baral S. Global Epidemiology of HIV Infection and Related Syndemics Affecting Transgender People. Journal of acquired immune deficiency syndromes (1999). 2016;72:S210-9. PubMed PMID: 27429185.

206. Moradi A, Alammehrjerdi Z, Daneshmand R, Amini-Lari M, Zarghami M, Dolan K. HIV Responses in Arab States on the Southern Persian Gulf Border: The First Review. Iran J Psychiatry Behav Sci. 2016;10(3):e5392. PubMed PMID: 27822284.

207. Lihana RW, Ssemwanga D, Abimiku A, Ndembi N. Update on HIV-1 diversity in Africa: a decade in review. AIDS reviews. 2012;14(2):83-100. PubMed PMID: 22627605.

208. Abu-Raddad LJ, Schiffer JT, Ashley R, Mumtaz G, Alsallaq RA, Akala FA, et al. HSV-2 serology can be predictive of HIV epidemic potential and hidden sexual risk behavior in the Middle East and North Africa. Epidemics. 2010;2(4):173-82. PubMed PMID: 21352788.

209. Caceres CF, Konda K, Segura ER, Lyerla R. Epidemiology of male same-sex behaviour and associated sexual health indicators in low- and middle-income countries: 2003-2007 estimates. Sexually transmitted infections. 2008;84:i49-i56. PubMed PMID: 18647866.

210. Serbessa MK, Mariam DH, Kassa A, Alwan F, Kloos H. HIV/AIDS among pastoralists and refugees in north-east Africa: a neglected problem. African journal of AIDS research : AJAR. 2016;15(1):45-54. PubMed PMID: 27002357.

211. Bruni L, Diaz M, Castellsague X, Ferrer E, Bosch FX, de Sanjose S. Cervical human papillomavirus prevalence in 5 continents: meta-analysis of 1 million women with normal cytological findings. The Journal of infectious diseases. 2010;202(12):1789-99. PubMed PMID: 21067372.

212. Smith JS, Melendy A, Rana RK, Pimenta JM. Age-specific prevalence of infection with human papillomavirus in females: a global review. The Journal of adolescent health : official publication of the Society for Adolescent Medicine. 2008;43(4):S5-25, S.e1-41. PubMed PMID: 18809145.

213. Ogembo RK, Gona PN, Seymour AJ, Park HS, Bain PA, Maranda L, et al. Prevalence of human papillomavirus genotypes among African women with normal cervical cytology and neoplasia: a systematic review and meta-analysis. PLoS One. 2015;10(4):e0122488. PubMed PMID: 25875167.

214. Seoud M. Burden of human papillomavirus-related cervical disease in the extended middle East and north Africa-a comprehensive literature review. Journal of lower genital tract disease. 2012;16(2):106-20. PubMed PMID: 22371041.

215. Alkaiyat A, Weiss MG. HIV in the Middle East and North Africa: priority, culture, and control. International journal of public health. 2013;58(6):927-37. PubMed PMID: 23824483.

216. Furuya-Kanamori L, Liang S, Milinovich G, Soares Magalhaes RJ, Clements AC, Hu W, et al. Co-distribution and co-infection of chikungunya and dengue viruses. BMC infectious diseases. 2016;16:84. PubMed PMID: 26936191.

217. Alhaeli A, Bahkali S, Ali A, Househ MS, El-Metwally AA. The epidemiology of Dengue fever in Saudi Arabia: A systematic review. J Infect Public Health. 2016;9(2):117-24. PubMed PMID: 26106040.

218. Humphrey JM, Cleton NB, Reusken CB, Glesby MJ, Koopmans MP, Abu-Raddad LJ. Dengue in the Middle East and North Africa: A Systematic Review. PLoS neglected tropical diseases. 2016;10(12):e0005194. PubMed PMID: 27926925.

219. Razavi SM, Saeednejad M, Salamati P. Vaccination in Hajj: An Overview of the Recent Findings. International journal of preventive medicine. 2016;7:129. PubMed PMID: 28105294.

220. Ghenghesh KS, Rahouma A, Tawil K, Zorgani A, Franka E. Antimicrobial resistance in Libya: 1970-2011. The Libyan journal of medicine. 2013;8:1-8. PubMed PMID: 23537612.

221. Tanios CY, Abou-Saleh MT, Karam AN, Salamoun MM, Mneimneh ZN, Karam EG. The epidemiology of anxiety disorders in the Arab world: a review. Journal of anxiety disorders. 2009;23(4):409-19. PubMed PMID: 19091509.

222. Elzubeir MA, Elzubeir KE, Magzoub ME. Stress and coping strategies among Arab medical students: towards a research agenda. Education for health (Abingdon, England). 2010;23(1):355. PubMed PMID: 20589606.

223. Farah LG, Fayyad JA, Eapen V, Cassir Y, Salamoun MM, Tabet CC, et al. ADHD in the Arab world: a review of epidemiologic studies. Journal of attention disorders. 2009;13(3):211-22. PubMed PMID: 19372498.

224. Alhraiwil NJ, Ali A, Househ MS, Al-Shehri AM, El-Metwally AA. Systematic review of the epidemiology of attention deficit hyperactivity disorder in Arab countries. Neurosciences (Riyadh, Saudi Arabia). 2015;20(2):137-44. PubMed PMID: 25864066.

225. Alkhateeb JM, Alhadidi MS. ADHD Research in Arab Countries: A Systematic Review of Literature. Journal of attention disorders. 2016;0. PubMed PMID: 26794672.

226. Bakare MO, Munir KM. Autism spectrum disorders (ASD) in Africa: a perspective. African journal of psychiatry. 2011;14(3):208-10. PubMed PMID: 21863205.

227. Salhia HO, Al-Nasser LA, Taher LS, Al-Khathaami AM, El-Metwally AA. Systemic review of the epidemiology of autism in Arab Gulf countries. Neurosciences (Riyadh, Saudi Arabia). 2014;19(4):291-6. PubMed PMID: 25274588.

228. Esan O, Esan A. Epidemiology and burden of bipolar disorder in Africa: a systematic review of data from Africa. Soc Psychiatry Psychiatr Epidemiol. 2016;51(1):93-100. PubMed PMID: 26155900.

229. Zahidie A, Jamali T. An overview of the predictors of depression among adult Pakistani women. Journal of the College of Physicians and Surgeons--Pakistan : JCPSP. 2013;23(8):574-80. PubMed PMID: 23930875.

230. Travers KU, Pokora TD, Cadarette SM, Mould JF. Major depressive disorder in Africa and the Middle East: a systematic literature review. Expert review of pharmacoeconomics & outcomes research. 2013;13(5):613-30. PubMed PMID: 24138647.

231. Dardas LA, Bailey DE, Jr., Simmons LA. Adolescent Depression in the Arab Region: A Systematic Literature Review. Issues Ment Health Nurs. 2016;37(8):569-85. PubMed PMID: 27168124.

232. Klainin P, Arthur DG. Postpartum depression in Asian cultures: a literature review. Int J Nurs Stud. 2009;46(10):1355-73. PubMed PMID: 19327773.

233. Cheng C, Li AY. Internet addiction prevalence and quality of (real) life: a meta-analysis of 31 nations across seven world regions. Cyberpsychology, behavior and social networking. 2014;17(12):755-60.

234. Nasser SC, Salamoun MM. Treatment of mental disorders and pathways to care in Arab countries. International journal of psychiatry in clinical practice. 2011;15(1):12-8. PubMed PMID: 22122684.

235. Dimitry L. A systematic review on the mental health of children and adolescents in areas of armed conflict in the Middle East. Child Care Health Dev. 2012;38(2):153-61. PubMed PMID: 21615769.

236. Kronfol Z, Saleh M, Al-Ghafry M. Mental health issues among migrant workers in Gulf Cooperation Council countries: literature review and case illustrations. Asian journal of psychiatry. 2014;10:109-13. PubMed PMID: 25042963.

237. Marie M, Hannigan B, Jones A. Mental health needs and services in the West Bank, Palestine. International journal of mental health systems. 2016;10:23. PubMed PMID: 26981151.

238. Hassan G, Ventevogel P, Jefee-Bahloul H, Barkil-Oteo A, Kirmayer LJ. Mental health and psychosocial wellbeing of Syrians affected by armed conflict. Epidemiology and psychiatric sciences. 2016;25(2):129-41. PubMed PMID: 26829998.

239. Ayer L, Venkatesh B, Stewart R, Mandel D, Stein B, Schoenbaum M. Psychological Aspects of the Israeli-Palestinian Conflict: A Systematic Review. Trauma, violence & abuse. 2015;0. PubMed PMID: 26511933.

240. Wells R, Steel Z, Abo-Hilal M, Hassan AH, Lawsin C. Psychosocial concerns reported by Syrian refugees living in Jordan: systematic review of unpublished needs assessments. The British journal of psychiatry : the journal of mental science. 2016;209(2):99-106. PubMed PMID: 27103679.

241. Rezaeian M. Suicide among young Middle Eastern Muslim females. Crisis. 2010;31(1):36-42. PubMed PMID: 20197256.

242. Morovatdar N, Moradi-Lakeh M, Malakouti SK, Nojomi M. Most common methods of suicide in Eastern Mediterranean Region of WHO: a systematic review and meta-analysis. Archives of suicide research : official journal of the International Academy for Suicide Research. 2013;17(4):335-44. PubMed PMID: 24224668.

243. Jordans MJ, Kaufman A, Brenman NF, Adhikari RP, Luitel NP, Tol WA, et al. Suicide in South Asia: a scoping review. BMC Psychiatry. 2014;14:358. PubMed PMID: 25539951.

244. Mars B, Burrows S, Hjelmeland H, Gunnell D. Suicidal behaviour across the African continent: a review of the literature. BMC Public Health. 2014;14:606. PubMed PMID: 24927746.

245. Fuhr DC, Calvert C, Ronsmans C, Chandra PS, Sikander S, De Silva MJ, et al. Contribution of suicide and injuries to pregnancy-related mortality in low-income and middle-income countries: a systematic review and meta-analysis. Lancet Psychiatry. 2014;1(3):213-25. PubMed PMID: 26360733.

246. Shahid M, Hyder AA. Deliberate self-harm and suicide: a review from Pakistan. Int J Inj Contr Saf Promot. 2008;15(4):233-41. PubMed PMID: 19051086.

247. Al-Khateeb JM, Al-Khateeb AJ. Research on psychosocial aspects of epilepsy in Arab countries: a review of literature. Epilepsy & behavior : E&B. 2014;31:256-62. PubMed PMID: 24210464.

248. Amawi N, Mollica RF, Lavelle J, Osman O, Nasir L. Overview of research on the mental health impact of violence in the Middle East in light of the Arab Spring. J Nerv Ment Dis. 2014;202(9):625-9. PubMed PMID: 25126755.

249. van der Meer IM, Middelkoop BJ, Boeke AJ, Lips P. Prevalence of vitamin D deficiency among Turkish, Moroccan, Indian and sub-Sahara African populations in Europe and their countries of origin: an overview. Osteoporos Int. 2011;22(4):1009-21. PubMed PMID: 20461360.

250. Badawi A, Arora P, Sadoun E, Al-Thani AA, Thani MH. Prevalence of vitamin d insufficiency in qatar: a systematic review. Journal of public health research. 2012;1(3):229-35. PubMed PMID: 25170469.

251. Bassil D, Rahme M, Hoteit M, Fuleihan Gel H. Hypovitaminosis D in the Middle East and North Africa: Prevalence, risk factors and impact on outcomes. Dermatoendocrinol. 2013;5(2):274-98. PubMed PMID: 24194968.

252. Hilger J, Friedel A, Herr R, Rausch T, Roos F, Wahl DA, et al. A systematic review of vitamin D status in populations worldwide. The British journal of nutrition. 2014;111(1):23-45. PubMed PMID: 23930771.

253. Palacios C, Gonzalez L. Is vitamin D deficiency a major global public health problem? The Journal of steroid biochemistry and molecular biology. 2014;144:138-45. PubMed PMID: 24239505.

254. Farrokhyar F, Tabasinejad R, Dao D, Peterson D, Ayeni OR, Hadioonzadeh R, et al. Prevalence of vitamin D inadequacy in athletes: a systematic-review and meta-analysis. Sports Med. 2015;45(3):365-78. PubMed PMID: 25277808.

255. Al-Daghri NM. Vitamin D in Saudi Arabia: Prevalence,distribution and disease associations. The Journal of steroid biochemistry and molecular biology. 2016;0. PubMed PMID: 28027916.

256. Agarwal A, Gupta SK, Sukumar R. Hyperparathyroidism and malnutrition with severe vitamin D deficiency. World journal of surgery. 2009;33(11):2303-13. PubMed PMID: 19404706.

257. Mehboob B, Safdar NF, Zaheer S. Socio-economic, environmental and demographic determinants of rise in obesity among Pakistani women: A Systematic Review. JPMA The Journal of the Pakistan Medical Association. 2016;66(9):1165-72. PubMed PMID: 27654739.

258. Hammad SS, Berry DC. The Child Obesity Epidemic in Saudi Arabia: A Review of the Literature. Journal of transcultural nursing : official journal of the Transcultural Nursing Society / Transcultural Nursing Society. 2016;0. PubMed PMID: 27655934.

259. Danaei G, Finucane MM, Lin JK, Singh GM, Paciorek CJ, Cowan MJ, et al. National, regional, and global trends in systolic blood pressure since 1980: systematic analysis of health examination surveys and epidemiological studies with 786 country-years and 5.4 million participants. Lancet. 2011;377(9765):568-77. PubMed PMID: 21295844.

260. Hasan DM, Emeash AH, Mustafa SB, Abdelazim GE, El-din AA. Hypertension in Egypt: a systematic review. Current hypertension reviews. 2014;10(3):134-41. PubMed PMID: 25544289.

261. Neupane D, McLachlan CS, Sharma R, Gyawali B, Khanal V, Mishra SR, et al. Prevalence of hypertension in member countries of South Asian Association for Regional Cooperation (SAARC): systematic review and meta-analysis. Medicine (Baltimore). 2014;93(13):e74. PubMed PMID: 25233326.

262. Tailakh A, Evangelista LS, Mentes JC, Pike NA, Phillips LR, Morisky DE. Hypertension prevalence, awareness, and control in Arab countries: a systematic review. Nursing & health sciences. 2014;16(1):126-30. PubMed PMID: 24118852.

263. Nansseu JR, Noubiap JJ, Mengnjo MK, Aminde LN, Essouma M, Jingi AM, et al. The highly neglected burden of resistant hypertension in Africa: a systematic review and meta-analysis. BMJ Open. 2016;6(9):e011452. PubMed PMID: 27650760.

264. Mabry RM, Reeves MM, Eakin EG, Owen N. Gender differences in prevalence of the metabolic syndrome in Gulf Cooperation Council Countries: a systematic review. Diabetic medicine : a journal of the British Diabetic Association. 2010;27(5):593-7. PubMed PMID: 20536958.

265. Mirmiran P, Sherafat-Kazemzadeh R, Jalali-Farahani S, Azizi F. Childhood obesity in the Middle East: a review. Eastern Mediterranean health journal = La revue de sante de la Mediterranee orientale = al-Majallah al-sihhiyah li-sharq al-mutawassit. 2010;16(9):1009-17. PubMed PMID: 21218730.

266. Karageorgi S, Alsmadi O, Behbehani K. A review of adult obesity prevalence, trends, risk factors, and epidemiologic methods in Kuwait. J Obes. 2013;2013:378650. PubMed PMID: 24455212.

267. Toselli S, Gualdi-Russo E, Boulos DN, Anwar WA, Lakhoua C, Jaouadi I, et al. Prevalence of overweight and obesity in adults from North Africa. Eur J Public Health. 2014;24:31-9. PubMed PMID: 25107996.

268. Mistry SK, Puthussery S. Risk factors of overweight and obesity in childhood and adolescence in South Asian countries: a systematic review of the evidence. Public Health. 2015;129(3):200-9. PubMed PMID: 25746156.

269. Alhyas L, McKay A, Balasanthiran A, Majeed A. Prevalences of overweight, obesity, hyperglycaemia, hypertension and dyslipidaemia in the Gulf: systematic review. JRSM short reports. 2011;2(7):55. PubMed PMID: 21847437.

270. Farzadfar F, Finucane MM, Danaei G, Pelizzari PM, Cowan MJ, Paciorek CJ, et al. National, regional, and global trends in serum total cholesterol since 1980: systematic analysis of health examination surveys and epidemiological studies with 321 country-years and 3.0 million participants. Lancet. 2011;377(9765):578-86. PubMed PMID: 21295847.

271. Bamimore MA, Zaid A, Banerjee Y, Al-Sarraf A, Abifadel M, Seidah NG, et al. Familial hypercholesterolemia mutations in the Middle Eastern and North African region: a need for a national registry. Journal of clinical lipidology. 2015;9(2):187-94. PubMed PMID: 25911074.

272. Abou Abbas L, Salameh P, Nasser W, Nasser Z, Godin I. Obesity and symptoms of depression among adults in selected countries of the Middle East: a systematic review and meta-analysis. Clinical obesity. 2015;5(1):2-11. PubMed PMID: 25504829.

273. Sadat-Ali M, Al-Habdan IM, Al-Turki HA, Azam MQ. An epidemiological analysis of the incidence of osteoporosis and osteoporosis-related fractures among the Saudi Arabian population. Ann Saudi Med. 2012;32(6):637-41. PubMed PMID: 23396029.

274. Baddoura R, Hoteit M, El-Hajj Fuleihan G. Osteoporotic fractures, DXA, and fracture risk assessment: meeting future challenges in the Eastern Mediterranean Region. Journal of clinical densitometry : the official journal of the International Society for Clinical Densitometry. 2011;14(4):384-94. PubMed PMID: 21839659.

275. Najjar H, Easson A. Age at diagnosis of breast cancer in Arab nations. International journal of surgery (London, England). 2010;8(6):448-52. PubMed PMID: 20601253.

276. Bhikoo R, Srinivasa S, Yu TC, Moss D, Hill AG. Systematic review of breast cancer biology in developing countries (part 1): Africa, the middle East, eastern europe, Mexico, the Caribbean and South america. Cancers. 2011;3(2):2358-81. PubMed PMID: 24212814.

277. Alhurishi S, Lim JN, Potrata B, West R. Factors influencing late presentation for breast cancer in the middle East: a systematic review. Asian Pacific journal of cancer prevention : APJCP. 2011;12(6):1597-600. PubMed PMID: 22126505.

278. Corbex M, Bouzbid S, Boffetta P. Features of breast cancer in developing countries, examples from North-Africa. European journal of cancer (Oxford, England : 1990). 2014;50(10):1808-18. PubMed PMID: 24767469.

279. Dubey AK, Gupta U, Jain S. Breast cancer statistics and prediction methodology: a systematic review and analysis. Asian Pacific journal of cancer prevention : APJCP. 2015;16(10):4237-45. PubMed PMID: 26028079.

280. Ting J, Kruzikas DT, Smith JS. A global review of age-specific and overall prevalence of cervical lesions. International journal of gynecological cancer : official journal of the International Gynecological Cancer Society. 2010;20(7):1244-9. PubMed PMID: 21495248.

281. Alhazzazi TY, Alghamdi FT. Head and Neck Cancer in Saudi Arabia: a Systematic Review. Asian Pacific journal of cancer prevention : APJCP. 2016;17(8):4043-8. PubMed PMID: 27644659.

282. Ashtari S, Pourhoseingholi MA, Sharifian A, Zali MR. Hepatocellular carcinoma in Asia: Prevention strategy and planning. World journal of hepatology. 2015;7(12):1708-17. PubMed PMID: 26140091.

283. Dubey AK, Gupta U, Jain S. Epidemiology of lung cancer and approaches for its prediction: a systematic review and analysis. Chinese journal of cancer. 2016;35(1):71. PubMed PMID: 27473753.

284. Ahmed HG. Aetiology of oral cancer in the Sudan. J Oral Maxillofac Res. 2013;4(2):e3. PubMed PMID: 24422031.

285. Krishna Rao SV, Mejia G, Roberts-Thomson K, Logan R. Epidemiology of oral cancer in Asia in the past decade--an update (2000-2012). Asian Pacific journal of cancer prevention : APJCP. 2013;14(10):5567-77. PubMed PMID: 24289546.

286. BenNasir E, El Mistiri M, McGowan R, Katz RV. Oral cancer in Libya and development of regional oral cancer registries: A review. The Saudi dental journal. 2015;27(4):171-9. PubMed PMID: 26644751.

287. Al-Jaber A, Al-Nasser L, El-Metwally A. Epidemiology of oral cancer in Arab countries. Saudi Med J. 2016;37(3):249-55. PubMed PMID: 26905345.

288. Sung PL, Chang YH, Chao KC, Chuang CM. Global distribution pattern of histological subtypes of epithelial ovarian cancer: a database analysis and systematic review. Gynecologic oncology. 2014;133(2):147-54. PubMed PMID: 24556058.

289. Cherbal F, Bakour R, Adane S, Boualga K. BRCA1 and BRCA2 germline mutation spectrum in hereditary breast/ovarian cancer families from Maghrebian countries. Breast Dis. 2012;34(1):1-8. PubMed PMID: 23697973.

290. Laraqui A, Uhrhammer N, Rhaffouli HE, Sekhsokh Y, Lahlou-Amine I, Bajjou T, et al. BRCA genetic screening in Middle Eastern and North African: mutational spectrum and founder BRCA1 mutation (c.798_799delTT) in North African. Dis Markers. 2015;2015:194293. PubMed PMID: 25814778.

291. Oluwagbemiga LA, Oluwole A, Kayode AA. Seventeen years after BRCA1: what is the BRCA mutation status of the breast cancer patients in Africa? - a systematic review. SpringerPlus. 2012;1(1):83. PubMed PMID: 23519070.

292. Eng A, McCormack V, dos-Santos-Silva I. Receptor-defined subtypes of breast cancer in indigenous populations in Africa: a systematic review and meta-analysis. PLoS medicine. 2014;11(9):e1001720. PubMed PMID: 25202974.

293. Midha A, Dearden S, McCormack R. EGFR mutation incidence in non-small-cell lung cancer of adenocarcinoma histology: a systematic review and global map by ethnicity (mutMapII). American journal of cancer research. 2015;5(9):2892-911. PubMed PMID: 26609494.

294. Irshad M, Mandal RK, Al-Drees A, Khalil MS, Abdulghani HM. No Evidence of Association of the Arg72Pro p53 Gene Polymorphism with Cancer Risk in the Saudi Population: a Meta-Analysis. Asian Pacific journal of cancer prevention : APJCP. 2015;16(14):5663-7. PubMed PMID: 26320432.

295. Shaik AP, Shaik AS, Al-Sheikh YA. Colorectal cancer: A review of the genome-wide association studies in the kingdom of Saudi Arabia. Saudi J Gastroenterol. 2015;21(3):123-8. PubMed PMID: 26021770.

296. Huang H, Hu XF, Zhao FH, Garland SM, Bhatla N, Qiao YL. Estimation of Cancer Burden Attributable to Infection in Asia. Journal of epidemiology / Japan Epidemiological Association. 2015;25(10):626-38. PubMed PMID: 26399446.

297. Hussein WM, Anwar WA, Attaleb M, Mazini L, Försti A, Trimbitas R-D, et al. A review of the infection-associated cancers in North African countries. Infectious agents and cancer. 2016;11(1):35.

298. Baandrup L, Thomsen LT, Olesen TB, Andersen KK, Norrild B, Kjaer SK. The prevalence of human papillomavirus in colorectal adenomas and adenocarcinomas: a systematic review and meta-analysis. European journal of cancer (Oxford, England : 1990). 2014;50(8):1446-61. PubMed PMID: 24560489.

299. Haddou Rahou B, El Rhazi K, Ouasmani F, Nejjari C, Bekkali R, Montazeri A, et al. Quality of life in Arab women with breast cancer: a review of the literature. Health and quality of life outcomes. 2016;14:64. PubMed PMID: 27117705.

300. Donnelly TT, Khater AH, Al-Bader SB, Al Kuwari MG, Al-Meer N, Malik M, et al. Arab women's breast cancer screening practices: a literature review. Asian Pacific journal of cancer prevention : APJCP. 2013;14(8):4519-28. PubMed PMID: 24083695.

301. Alananzeh I, Levesque J, Kwok C, Everett B. Integrative Review of the Supportive Care Needs of Arab People Affected by Cancer. Asia-Pacific journal of oncology nursing. 2016;3(2):148-56. PubMed PMID: 27981153.

302. Peleteiro B, Bastos J, Barros H, Lunet N. Systematic review of the prevalence of gastric intestinal metaplasia and its area-level association with smoking. Gaceta sanitaria / SESPAS. 2008;22(3):236-47; discussion 46-7. PubMed PMID: 18579050.

303. Almaguer M, Herrera R, Orantes CM. Chronic kidney disease of unknown etiology in agricultural communities. MEDICC review. 2014;16(2):9-15. PubMed PMID: 24878644.

304. Hassanien AA, Al-Shaikh F, Vamos EP, Yadegarfar G, Majeed A. Epidemiology of end-stage renal disease in the countries of the Gulf Cooperation Council: a systematic review. JRSM short reports. 2012;3(6):38. PubMed PMID: 22768372.

305. Goleg FA, Kong NC, Sahathevan R. Dialysis-treated end-stage kidney disease in Libya: epidemiology and risk factors. International urology and nephrology. 2014;46(8):1581-7. PubMed PMID: 24671275.

306. Okpechi IG, Ameh OI, Bello AK, Ronco P, Swanepoel CR, Kengne AP. Epidemiology of Histologically Proven Glomerulonephritis in Africa: A Systematic Review and Meta-Analysis. PLoS One. 2016;11(3):e0152203. PubMed PMID: 27011216.

307. McGrogan A, Franssen CF, de Vries CS. The incidence of primary glomerulonephritis worldwide: a systematic review of the literature. Nephrology, dialysis, transplantation : official publication of the European Dialysis and Transplant Association - European Renal Association. 2011;26(2):414-30. PubMed PMID: 21068142.

308. Faust WC, Diaz M, Pohl HG. Incidence of post-pyelonephritic renal scarring: a meta-analysis of the dimercapto-succinic acid literature. The Journal of urology. 2009;181(1):290-7; discussion 7-8. PubMed PMID: 19013606.

309. Gwer S, Chacha C, Newton CR, Idro R. Childhood acute non-traumatic coma: aetiology and challenges in management in resource-poor countries of Africa and Asia. Paediatrics and international child health. 2013;33(3):129-38. PubMed PMID: 23930724.

310. Marin B, Boumediene F, Logroscino G, Couratier P, Babron MC, Leutenegger AL, et al. Variation in worldwide incidence of amyotrophic lateral sclerosis: a meta-analysis. International journal of epidemiology. 2016;0. PubMed PMID: 27185810.

311. Benamer HT, Grosset DG. A systematic review of the epidemiology of epilepsy in Arab countries. Epilepsia. 2009;50(10):2301-4. PubMed PMID: 19389149.

312. Angalakuditi M, Angalakuditi N. A comprehensive review of the literature on epilepsy in selected countries in emerging markets. Neuropsychiatric disease and treatment. 2011;7:585-97. PubMed PMID: 22003298.

313. Bhalla D, Lotfalinezhad E, Timalsina U, Kapoor S, Kumar KS, Abdelrahman A, et al. A comprehensive review of epilepsy in the Arab world. Seizure. 2016;34:54-9. PubMed PMID: 26724591.

314. Benamer HT, Bredan A. Guillain-Barre syndrome in Arab countries: a systematic review. J Neurol Sci. 2014;343(1):221-3. PubMed PMID: 24950899.

315. Webb AJ, Brain SA, Wood R, Rinaldi S, Turner MR. Seasonal variation in Guillain-Barre syndrome: a systematic review, meta-analysis and Oxfordshire cohort study. Journal of neurology, neurosurgery, and psychiatry. 2015;86(11):1196-201. PubMed PMID: 25540247.

316. Benamer HT, Deleu D, Grosset D. Epidemiology of headache in Arab countries. J Headache Pain. 2010;11(1):1-3. PubMed PMID: 19949829.

317. Al-Hashel J, Besterman AD, Wolfson C. The prevalence of multiple sclerosis in the Middle East. Neuroepidemiology. 2008;31(2):129-37. PubMed PMID: 18716409.

318. Heydarpour P, Khoshkish S, Abtahi S, Moradi-Lakeh M, Sahraian MA. Multiple Sclerosis Epidemiology in Middle East and North Africa: A Systematic Review and Meta-Analysis. Neuroepidemiology. 2015;44(4):232-44. PubMed PMID: 26088327.

319. Eskandarieh S, Heydarpour P, Minagar A, Pourmand S, Sahraian MA. Multiple Sclerosis Epidemiology in East Asia, South East Asia and South Asia: A Systematic Review. Neuroepidemiology. 2016;46(3):209-21. PubMed PMID: 26901651.

320. Benamer HT, Ahmed ES, Al-Din AS, Grosset DG. Frequency and clinical patterns of multiple sclerosis in Arab countries: a systematic review. J Neurol Sci. 2009;278(1):1-4. PubMed PMID: 19135686.

321. Benamer HT, de Silva R, Siddiqui KA, Grosset DG. Parkinson's disease in Arabs: a systematic review. Movement disorders : official journal of the Movement Disorder Society. 2008;23(9):1205-10. PubMed PMID: 18442138.

322. Benamer HT, Bredan A. The epidemiology of myasthenia gravis in Arab countries: a systematic review. Muscle & nerve. 2015;51(1):144-5. PubMed PMID: 25088247.

323. Correia Guedes L, Ferreira JJ, Rosa MM, Coelho M, Bonifati V, Sampaio C. Worldwide frequency of G2019S LRRK2 mutation in Parkinson's disease: a systematic review. Parkinsonism Relat Disord. 2010;16(4):237-42. PubMed PMID: 19945904.

324. Amara AH, Aljunid SM. Noncommunicable diseases among urban refugees and asylum-seekers in developing countries: a neglected health care need. Globalization and health. 2014;10:24. PubMed PMID: 24708876.

325. Al-Qasem A, Smith F, Clifford S. Adherence to medication among chronic patients in Middle Eastern countries: review of studies. Eastern Mediterranean health journal = La revue de sante de la Mediterranee orientale = al-Majallah al-sihhiyah li-sharq al-mutawassit. 2011;17(4):356-63. PubMed PMID: 22259896.

326. Ng SW, Zaghloul S, Ali HI, Harrison G, Popkin BM. The prevalence and trends of overweight, obesity and nutrition-related non-communicable diseases in the Arabian Gulf States. Obes Rev. 2011;12(1):1-13. PubMed PMID: 20546144.

327. Boutayeb A, Boutayeb S, Boutayeb W. Multi-morbidity of non communicable diseases and equity in WHO Eastern Mediterranean countries. Int J Equity Health. 2013;12:60. PubMed PMID: 23961989.

328. John LJ, Shantakumari N. Herbal Medicines Use During Pregnancy: A Review from the Middle East. Oman medical journal. 2015;30(4):229-36. PubMed PMID: 26366255.

329. Nazeri P, Mirmiran P, Shiva N, Mehrabi Y, Mojarrad M, Azizi F. Iodine nutrition status in lactating mothers residing in countries with mandatory and voluntary iodine fortification programs: an updated systematic review. Thyroid. 2015;25(6):611-20. PubMed PMID: 25811835.

330. Mirmiran P, Golzarand M, Serra-Majem L, Azizi F. Iron, iodine and vitamin a in the middle East; a systematic review of deficiency and food fortification. Iranian journal of public health. 2012;41(8):8-19. PubMed PMID: 23113219.

331. Nielsen J, Prudhon C, de Radigues X. Trends in malnutrition and mortality in Darfur, Sudan, between 2004 and 2008: a meta-analysis of publicly available surveys. International journal of epidemiology. 2011;40(4):971-84. PubMed PMID: 21296853.

332. Tsigga M, Grammatikopoulou MG. Assessing the silent epidemic of malnutrition in Palestinian preschool children. Journal of epidemiology and global health. 2012;2(4):181-91. PubMed PMID: 23856499.

333. Akhtar S. Malnutrition in South Asia-A Critical Reappraisal. Critical reviews in food science and nutrition. 2016;56(14):2320-30. PubMed PMID: 25830938.

334. Musaiger AO, Hassan AS, Obeid O. The paradox of nutrition-related diseases in the Arab countries: the need for action. International journal of environmental research and public health. 2011;8(9):3637-71. PubMed PMID: 22016708.

335. Hirani SA. Malnutrition in young Pakistani children. Journal of Ayub Medical College, Abbottabad : JAMC. 2012;24(2):150-3. PubMed PMID: 24397079.

336. Best C, Neufingerl N, van Geel L, van den Briel T, Osendarp S. The nutritional status of school-aged children: why should we care? Food and nutrition bulletin. 2010;31(3):400-17. PubMed PMID: 20973461.

337. Stark KD, Van Elswyk ME, Higgins MR, Weatherford CA, Salem N, Jr. Global survey of the omega-3 fatty acids, docosahexaenoic acid and eicosapentaenoic acid in the blood stream of healthy adults. Progress in lipid research. 2016;63:132-52. PubMed PMID: 27216485.

338. Creo AL, Thacher TD, Pettifor JM, Strand MA, Fischer PR. Nutritional rickets around the world: an update. Paediatrics and international child health. 2016;0:1-15. PubMed PMID: 27922335.

339. Stoffaneller R, Morse NL. A review of dietary selenium intake and selenium status in Europe and the Middle East. Nutrients. 2015;7(3):1494-537. PubMed PMID: 25734564.

340. Powles J, Fahimi S, Micha R, Khatibzadeh S, Shi P, Ezzati M, et al. Global, regional and national sodium intakes in 1990 and 2010: a systematic analysis of 24 h urinary sodium excretion and dietary surveys worldwide. BMJ Open. 2013;3(12):e003733. PubMed PMID: 24366578.

341. Sharma IK, Byrne A. Early initiation of breastfeeding: a systematic literature review of factors and barriers in South Asia. International breastfeeding journal. 2016;11:17. PubMed PMID: 27330542.

342. Ahmad K, A BZ, D JMT, Chand B. A systematic review of epidemiological literature on the eye health of marginalized fishing populations. JPMA The Journal of the Pakistan Medical Association. 2016;66(10):S81-s3. PubMed PMID: 27895363.

343. Stevens GA, White RA, Flaxman SR, Price H, Jonas JB, Keeffe J, et al. Global prevalence of vision impairment and blindness: magnitude and temporal trends, 1990-2010. Ophthalmology. 2013;120(12):2377-84. PubMed PMID: 23850093.

344. Bourne R, Price H, Taylor H, Leasher J, Keeffe J, Glanville J, et al. New systematic review methodology for visual impairment and blindness for the 2010 Global Burden of Disease study. Ophthalmic epidemiology. 2013;20(1):33-9. PubMed PMID: 23350553.

345. Cheng JW, Zong Y, Zeng YY, Wei RL. The prevalence of primary angle closure glaucoma in adult Asians: a systematic review and meta-analysis. PLoS One. 2014;9(7):e103222. PubMed PMID: 25057993.

346. Khairallah M, Kahloun R, Flaxman SR, Jonas JB, Keeffe J, Leasher J, et al. Prevalence and causes of vision loss in North Africa and the Middle East: 1990-2010. The British journal of ophthalmology. 2014;98(5):605-11. PubMed PMID: 24590555.

347. Gilbert CE, Lepvrier-Chomette N. Gender Inequalities in Surgery for Bilateral Cataract among Children in Low-Income Countries: A Systematic Review. Ophthalmology. 2016;123(6):1245-51. PubMed PMID: 26992842.

348. Khan SQ, Khan NB, Arrejaie AS. Dental caries. A meta analysis on a Saudi population. Saudi Med J. 2013;34(7):744-9. PubMed PMID: 23860895.

349. Al-Bluwi GS. Epidemiology of dental caries in children in the United Arab Emirates. International dental journal. 2014;64(4):219-28. PubMed PMID: 24860920.

350. Khan SQ. Dental caries in Arab League countries: a systematic review and meta-analysis. International dental journal. 2014;64(4):173-80. PubMed PMID: 24506766.

351. Al Agili DE. A systematic review of population-based dental caries studies among children in Saudi Arabia. The Saudi dental journal. 2013;25(1):3-11. PubMed PMID: 23960549.

352. Barzangi J, Unell L, Soderfeldt B, Arnrup K. Infant dental enucleation: A literature review on a traditional remedial practice in East Africa. Acta odontologica Scandinavica. 2014;72(3):168-78. PubMed PMID: 23865550.

353. Al-Nasser L, El-Metwally A. Oral lichen planus in Arab countries : a review. Journal of oral pathology & medicine : official publication of the International Association of Oral Pathologists and the American Academy of Oral Pathology. 2014;43(10):723-7. PubMed PMID: 24245546.

354. Al-Harthi LS, Cullinan MP, Leichter JW, Thomson WM. Periodontitis among adult populations in the Arab World. International dental journal. 2013;63(1):7-11. PubMed PMID: 23410016.

355. Mohamed Zaki LR, Hairi NN. A Systematic Review of the Prevalence and Measurement of Chronic Pain in Asian Adults. Pain management nursing : official journal of the American Society of Pain Management Nurses. 2015;16(3):440-52. PubMed PMID: 25439125.

356. Sisson SB, Katzmarzyk PT. International prevalence of physical activity in youth and adults. Obes Rev. 2008;9(6):606-14. PubMed PMID: 18647243.

357. Mabry RM, Reeves MM, Eakin EG, Owen N. Evidence of physical activity participation among men and women in the countries of the Gulf cooperation council: a review. Obes Rev. 2010;11(6):457-64. PubMed PMID: 19793376.

358. Ranasinghe CD, Ranasinghe P, Jayawardena R, Misra A. Physical activity patterns among South-Asian adults: a systematic review. Int J Behav Nutr Phys Act. 2013;10:116. PubMed PMID: 24119682.

359. Yammine K. The prevalence of physical activity among the young population of UAE: a meta-analysis. Perspectives in public health. 2016;0. PubMed PMID: 27810999.

360. Mabry R, Koohsari MJ, Bull F, Owen N. A systematic review of physical activity and sedentary behaviour research in the oil-producing countries of the Arabian Peninsula. BMC Public Health. 2016;16(1):1003. PubMed PMID: 27655373.

361. Loney T, Aw TC, Handysides DG, Ali R, Blair I, Grivna M, et al. An analysis of the health status of the United Arab Emirates: the 'Big 4' public health issues. Glob Health Action. 2013;6:20100. PubMed PMID: 23394856.

362. Adeloye D, Chua S, Lee C, Basquill C, Papana A, Theodoratou E, et al. Global and regional estimates of COPD prevalence: Systematic review and meta-analysis. Journal of global health. 2015;5(2):020415. PubMed PMID: 26755942.

363. Akhter E, Bilal S, Kiani A, Haque U. Prevalence of arthritis in India and Pakistan: a review. Rheumatology international. 2011;31(7):849-55. PubMed PMID: 21331574.

364. Usenbo A, Kramer V, Young T, Musekiwa A. Prevalence of Arthritis in Africa: A Systematic Review and Meta-Analysis. PLoS One. 2015;10(8):e0133858. PubMed PMID: 26241756.

365. Almoallim HM, Alharbi LA. Rheumatoid arthritis in Saudi Arabia. Saudi Med J. 2014;35(12):1442-54.

366. Stolwijk C, van Onna M, Boonen A, van Tubergen A. Global Prevalence of Spondyloarthritis: A Systematic Review and Meta-Regression Analysis. Arthritis Care Res (Hoboken). 2016;68(9):1320-31. PubMed PMID: 26713432.

367. Mustafa KN. Takayasu's arteritis in Arabs. Clinical rheumatology. 2014;33(12):1777-83. PubMed PMID: 24803229.

368. Osio-Salido E, Manapat-Reyes H. Epidemiology of systemic lupus erythematosus in Asia. Lupus. 2010;19(12):1365-73. PubMed PMID: 20947544.

369. Rafeey M, Ghojazadeh M, Mehdizadeh A, Hazrati H, Vahedi L. Intercontinental comparison of caustic ingestion in children. Korean J Pediatr. 2015;58(12):491-500. PubMed PMID: 26770225.

370. Kadir MM, Janjua NZ, Kristensen S, Fatmi Z, Sathiakumar N. Status of children's blood lead levels in Pakistan: implications for research and policy. Public Health. 2008;122(7):708-15. PubMed PMID: 18359052.

371. Chippaux JP, Goyffon M. Epidemiology of scorpionism: a global appraisal. Acta tropica. 2008;107(2):71-9. PubMed PMID: 18579104.

372. Othman N, Kendrick D. Epidemiology of burn injuries in the East Mediterranean Region: a systematic review. BMC Public Health. 2010;10:83. PubMed PMID: 20170527.

373. Golshan A, Patel C, Hyder AA. A systematic review of the epidemiology of unintentional burn injuries in South Asia. J Public Health (Oxf). 2013;35(3):384-96. PubMed PMID: 23321681.

374. Tapp C, Burkle FM, Jr., Wilson K, Takaro T, Guyatt GH, Amad H, et al. Iraq War mortality estimates: a systematic review. Conflict and health. 2008;2:1. PubMed PMID: 18328100.

375. Fehling M, Jarrah ZM, Tiernan ME, Albezreh S, VanRooyen MJ, Alhokair A, et al. Youth in crisis in the Middle East and North Africa: a systematic literature review and focused landscape analysis. Eastern Mediterranean health journal = La revue de sante de la Mediterranee orientale = al-Majallah al-sihhiyah li-sharq al-mutawassit. 2016;21(12):916-30. PubMed PMID: 26996365.

376. Cheng SY, Levy AR, Lefaivre KA, Guy P, Kuramoto L, Sobolev B. Geographic trends in incidence of hip fractures: a comprehensive literature review. Osteoporos Int. 2011;22(10):2575-86. PubMed PMID: 21484361.

377. Kulczycki A, Windle S. Honor killings in the Middle East and North Africa: a systematic review of the literature. Violence Against Women. 2011;17(11):1442-64. PubMed PMID: 22312039.

378. Ali PA, Naylor PB, Croot E, O'Cathain A. Intimate Partner Violence in Pakistan: A Systematic Review. Trauma, violence & abuse. 2015;16(3):299-315. PubMed PMID: 24626459.

379. Roman NV, Frantz JM. The prevalence of intimate partner violence in the family: a systematic review of the implications for adolescents in Africa. Fam Pract. 2013;30(3):256-65. PubMed PMID: 23363539.

380. Boy A, Kulczycki A. What we know about intimate partner violence in the Middle East and North Africa. Violence Against Women. 2008;14(1):53-70. PubMed PMID: 18096859.

381. Majzoub AA, Canguven O, Raidh TA. Alteration in the etiology of penile fracture in the Middle East and Central Asia regions in the last decade; a literature review. Urol Ann. 2015;7(3):284-8. PubMed PMID: 26229311.

382. McAlpine A, Hossain M, Zimmerman C. Sex trafficking and sexual exploitation in settings affected by armed conflicts in Africa, Asia and the Middle East: systematic review. BMC Int Health Hum Rights. 2016;16(1):34. PubMed PMID: 28031024.

383. Mansuri FA, Al-Zalabani AH, Zalat MM, Qabshawi RI. Road safety and road traffic accidents in Saudi Arabia. A systematic review of existing evidence. Saudi Med J. 2015;36(4):418-24. PubMed PMID: 25828277.

384. Puvanachandra P, Hoe C, El-Sayed HF, Saad R, Al-Gasseer N, Bakr M, et al. Road traffic injuries and data systems in Egypt: addressing the challenges. Traffic Inj Prev. 2012;13:44-56. PubMed PMID: 22414128.

385. Abou-Abbass H, Bahmad H, Ghandour H, Fares J, Wazzi-Mkahal R, Yacoub B, et al. Epidemiology and clinical characteristics of traumatic brain injury in Lebanon: A systematic review. Medicine (Baltimore). 2016;95(47):e5342. PubMed PMID: 27893670.

386. Cripps RA, Lee BB, Wing P, Weerts E, Mackay J, Brown D. A global map for traumatic spinal cord injury epidemiology: towards a living data repository for injury prevention. SPINAL CORD. 2011;49(4):493-501. PubMed PMID: 21102572.

387. Hillis S, Mercy J, Amobi A, Kress H. Global Prevalence of Past-year Violence Against Children: A Systematic Review and Minimum Estimates. Pediatrics. 2016;137(3):e20154079. PubMed PMID: 26810785.
